# CpG-Methylation Regulates a Class of Epstein-Barr Virus Promoters

**DOI:** 10.1371/journal.ppat.1001114

**Published:** 2010-09-23

**Authors:** Martin Bergbauer, Markus Kalla, Anne Schmeinck, Christine Göbel, Ulrich Rothbauer, Sebastian Eck, Anna Benet-Pagès, Tim M. Strom, Wolfgang Hammerschmidt

**Affiliations:** 1 Department of Gene Vectors, Helmholtz Zentrum München, German Research Center for Environmental Health, Munich, Germany; 2 Biocenter at the Department of Biology II, Ludwig-Maximilians University Munich, Martinsried, Germany; 3 Institute of Human Genetics, Helmholtz Zentrum München, German Research Center for Environmental Health, Neuherberg, Germany; Emory University, United States of America

## Abstract

DNA methylation is the major modification of eukaryotic genomes and plays an essential role in mammalian gene regulation. In general, cytosine-phosphatidyl-guanosine (CpG)-methylated promoters are transcriptionally repressed and nuclear proteins such as MECP2, MBD1, MBD2, and MBD4 bind CpG-methylated DNA and contribute to epigenetic silencing. Methylation of viral DNA also regulates gene expression of Epstein-Barr virus (EBV), which is a model of herpes virus latency. In latently infected human B cells, the viral DNA is CpG-methylated, the majority of viral genes is repressed and virus synthesis is therefore abrogated. EBV's *BZLF1* encodes a transcription factor of the AP-1 family (Zta) and is the master gene to overcome viral gene repression. In a genome-wide screen, we now identify and characterize those viral genes, which Zta regulates. Among them are genes essential for EBV's lytic phase, which paradoxically depend on strictly CpG-methylated promoters for their Zta-induced expression. We identified novel DNA recognition motifs, termed meZRE (methyl-Zta-responsive element), which Zta selectively binds in order to ‘read’ DNA in a methylation- and sequence-dependent manner unlike any other known protein. Zta is a homodimer but its binding characteristics to meZREs suggest a sequential, non-palindromic and bipartite DNA recognition element, which confers superior DNA binding compared to CpG-free ZREs. Our findings indicate that Zta has evolved to transactivate cytosine-methylated, hence repressed, silent promoters as a rule to overcome epigenetic silencing.

## Author Summary

Epstein-Barr virus (EBV) is a model of human tumor viruses and viral latency. We have found previously that (i) EBV DNA is unmethylated upon infection, but becomes methylated over time by the host B cell, and (ii) the BZLF1-encoded protein, Zta (a cousin to AP-1), induces the viral lytic cycle, but it prefers binding sites that are CpG-methylated. As a consequence, the lytic phase gets delayed until enough methylation of viral DNA has occurred. The molecular basis of this mechanism has not been addressed but in a genome-wide screen, we now identify and characterize those viral genes, which Zta regulates. Among them are genes essential for EBV's lytic phase, which paradoxically depend on strictly CpG-methylated promoters for their Zta-induced expression. Our findings indicate that Zta has evolved to transactivate cytosine-methylated, hence repressed, silent promoters as a rule to overcome epigenetic silencing. In this way, the EBV virus has created its own time-dependent, epigenetic switch to control its biphasic life cycle.

## Introduction

The methylation of cytosines in CpG dinucleotides in mammalian DNA has long been associated with the regulation of transcription of that DNA. The details of this regulation, however, are only now being uncovered. For example genes first expressed in murine primordial germ cells after migration to the urogenital ridge are repressed prior to their migration by methylation of CpGs; the loss of methylation at their promoters correlates with these genes being transcribed [1]. An example in which expressed genes are repressed during differentiation is provided by murine embryonic stem cells induced to differentiate into neurons [2]. In this example, 2.3% of analyzed promoters become hypermethylated following differentiation with the associated genes being transcriptionally repressed leading to a loss of pluripotency. These examples demonstrate that CpG methylation is strictly linked to epigenetic gene silencing but are difficult to dissect mechanistically in part because of the complexity of the cues for differentiation that underlie them.

We have examined the fundamental events in the life-cycle of the human tumor virus, Epstein-Barr virus (EBV), in which it first establishes its infection in B-lymphocytes, drives them to proliferate, and then evolves to support its own productive infection in these cells. These experiments have allowed a dissection of the role of methylation of CpG dinucleotides in repressing and activating transcription of genes required for EBV's life cycle. Our initial experiments demonstrated that EBV uses the gradual methylation of its genome both to ensure its initial infection does not produce viral progeny to kill its host and later to activate transcription of genes necessary such that it can produce viral progeny [3].

EBV-infected cells can express two sets of viral genes that relate either to the latent or lytic phases of EBV's life cycle [4]. In newly infected B cells, EBV establishes a strictly latent infection. In these cells few viral genes termed latent genes are expressed, which are instrumental for the induction and maintenance of cellular proliferation and viral latency; some of which are also causally associated with EBV's being a human tumor virus [5]. Latently infected B cells can give rise to progeny virus, a process which requires the induction of a set of viral genes distinct from the set of EBV's latent genes. During *de novo* virus synthesis, about 80 lytic genes of EBV are expressed that asynchronously support viral DNA amplification and encode viral structural components to allow virus morphogenesis and release of progeny virus. The transition from viral latency to productive, lytic infection is orchestrated by two viral genes, *BZLF1* and *BRLF1*, which encode the transcription factors, Zta (also called Z, ZEBRA, or EB1) and Rta (also called R), respectively. The former is a master regulator of EBV's switch needed to induce the lytic phase of its life cycle in latently infected B cells [6], [7].

Zta is a basic leucine zipper (bZIP) transcription factor, which is modular in structure with a dedicated transactivation domain and a basic region that mediates DNA contact adjacent to a coiled-coil dimerization domain (for a review see [8]). Three important features of Zta include its ability to (i) bind sequence-specifically to Zta-response elements (ZREs) [9], (ii) transactivate viral genes [10], and (iii) serve as a replication factor activating the lytic origin of viral DNA replication [11]. Zta and Rta have non-redundant functions but cooperate to disrupt virus latency and control the regulated expression of all lytic viral genes [12].

The control of expression of viral genes needed to establish EBV's latency and eventually to allow its escape from latency has been unclear. A global screen revealed that the viral DNA is unmethylated in virions and acquires methylated CpG dinucleotides in latently EBV-infected B cells slowly over time [3]. Surprisingly, the functions of *BZLF1* and the methylation at CpGs are intimately connected. Previous papers suggested that Zta can bind to DNA even if it is CpG-methylated [13]–[15]. Our experimental data indicated that CpG methylation of genomic EBV DNA is an essential prerequisite for EBV's lytic phase including the synthesis of progeny virus [3]. CpG methylation of the promoter of the *BRLF1* gene was proposed to be critical for Zta regulating it [13], [15] but a detailed genetic analysis of the *BRLF1* promoter did not support this proposition [3]. Therefore we set out to identify viral genes, which both are regulated by Zta and are dependent for expression on the state of CpG methylation of genomic EBV DNA.

In genome-wide screens with unmethylated and methylated recombinant EBV DNA we now report that Zta can bind to many sites if they include methylated CpGs. These screen include chromatin-immunoprecipitations coupled to deep sequencing (ChIP-seq), which have identified highly selective binding of Zta to certain CpG-methylated promoters of lytic viral genes, some of which are essential for the completion of EBV's lytic phase. Detailed biomathematical, functional and biochemical analyses have revealed a novel CG dinucleotide-containing motif, termed meZRE, to which Zta binds exclusively and with superior affinity if it includes methylated cytosines. Our experiments suggest that transcription factors can directly activate epigenetically silenced genes in a sequence- and CpG-methylation-dependent manner.

Zta is a homodimer but its biochemical binding characteristics to meZREs suggests a sequential, non-palindromic and bipartite DNA recognition element which confers superior DNA binding compared to CpG-free ZREs. Our findings indicate that Zta has evolved to bind to CpG-methylated DNA and transactivate repressed promoters with meZRE motifs as a rule, which is a novel means to overcome epigenetic silencing.

## Results

### A differential, genome-wide screen with recombinant EBV DNA identifies putative binding sites for Zta

The extensive methylation of genomic EBV DNA during latent infection has been associated with a repression of viral transcription ([16], [17] for a recent review). In contrast, our previous work indicated CpG methylation of viral DNA as a prerequisite for synthesis of progeny EBV in latently infected cells [3]. Studies with a mutant EBV revealed that the methylation of viral DNA necessary for induction of EBV's lytic cycle is at sites other than those in the ZREs in the *BRLF1* promoter [3]. Therefore, viral genes downstream of *BRLF1* must be responsible for lytic gene expression from CpG-methylated viral templates.

We wanted to identify all potential sequence elements in EBV that favor the binding of Zta and used chromatin-immunoprecipitation coupled to next generation sequencing (ChIP-seq) as the most promising approach to do so. The 165 kbp genomic DNA of the EBV B95.8 strain cloned in E.coli [18] and free of methylated CpGs was used directly or after full CpG methylation *in vitro*. A chimeric GFP:BZLF1 protein (Supporting Figure S1A) was transiently expressed in HEK293 cells and purified with the aid of a GFP-binding recombinant antibody [19]. Immobilized GFP:BZLF1 was used to precipitate selectively EBV DNA fragments sheared to a size of approximately 250 bp as described before [3]. Bound DNAs were eluted, purified and subjected to library preparation for extensive next generation sequencing on a GAIIx sequencer (Illumina). We performed 36-bp single-end reads, which were mapped to the B95.8 EBV reference sequence [20].

**Figure 1:**
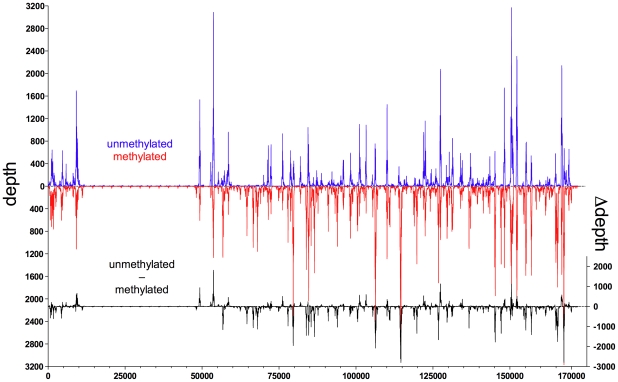
Zta binds preferentially to CpG-methylated EBV DNA *in vitro*. *In vitro* immunoprecipitation assays with GFP:BZLF1 and subsequent deep sequencing analysis indicate the preferred binding of Zta to CpG-methylated EBV DNA. E.coli-derived genomic EBV DNA free of methylated CpG dinucleotides (blue) and after complete *in vitro* CpG methylation by the methyltransferase M.SssI (red) was used as probes for the GFP:BZLF1 immunoprecipitations. Reads were mapped to the reference B95.8 EBV genome. Depicted is the read depth at single base pair resolution. In addition, the difference of read depth between the two experiments (Δdepth, black) is calculated and shown at the bottom.

In order to represent the data graphically, we normalized the read depth according to the average of the maximal read depths of the experiments, where ‘depth’ was defined as the number of times a single nucleotide residue in EBV DNA was identified. We observed a high dynamic range of read depth of approximately 1∶10^3^. The background read depth was approximately 10 with interspersed peaks with a read depth of up to 9000 (Figure 1). The calculated difference of depths between both kinds of EBV DNAs (Figure 1) indicated that Zta bound methylated viral DNA preferentially at multiple sites. In contrast, CpG-methylation of EBV DNA reduced or abrogated the binding of Zta in only a few positions (Figure 1).

**Figure 2:**
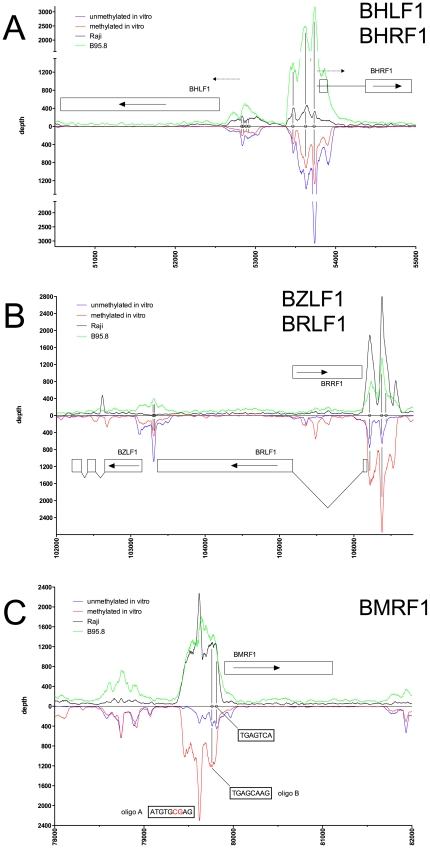
Identification of methylation-dependent Zta binding to selected viral promoter elements *in vivo* and *in vitro*. Deep sequencing data obtained from *in vitro* immunoprecipitation experiments with GFP:BZLF1 as in Figure 1 are plotted on selected genes and their promoters as indicated. EBV DNA free of CpG-methylation (blue) and after full methylation by the methyltransferase M.SssI (red) were used as probes in the experiments. *In vivo* ChIP-seq data obtained after immunoprecipitation of chromatin of Raji cells (black) or B95.8 cells (green) with GFP:BZLF1 are plotted. Ten genes, their exon compositions and the location of selected ZREs are shown. (A) The two divergently transcribed genes *BHLF1* and *BHRF1*, which bracket EBV's lytic origin of DNA replication are shown. (B) The two genes *BZLF1* and *BHRF1* and the intervening gene *BRRF1* are shown. (C) The *BMRF1* gene is shown, which encodes the viral DNA polymerase accessory protein.

Figures 2 to [Fig ppat-1001114-g003] [Fig ppat-1001114-g004] 5 show exemplary regions of known and novel ZREs binding sites in higher resolution. Since the discovery of Zta as sequence-specific DNA binding protein by two groups in 1989 [9], [21], a number of ZREs have been described in the EBV genome (Table S1 in Text S1). For example, our analysis confirmed Zta binding sites in the divergently oriented promoters of the *BHLF1* and *BRLF1* genes (Figure 2A) [22], which co-localize with the lytic origin of DNA replication of EBV, *oriLyt* [23]. The four ZREs in the *BHLF1* promoter, which reside within a 100bp span, were not individually resolved but ZRE5, -6, and -7 of *oriLyt* could be confirmed with high precision. The two very closely situated ZREs in the *BZLF1* promoter [21], [24] localized to a single peak (Figure 2B). Measurements of binding to unmethylated and CpG-methylated DNAs did not reveal major differences in the three promoters of *BHLF1*, *BHRF1* and *BZLF1* (blue and red lines, respectively, in Figure 2A, B) assuming that twofold variations of the peak depth values resulted from experimental variability.

**Figure 3 ppat-1001114-g003:**
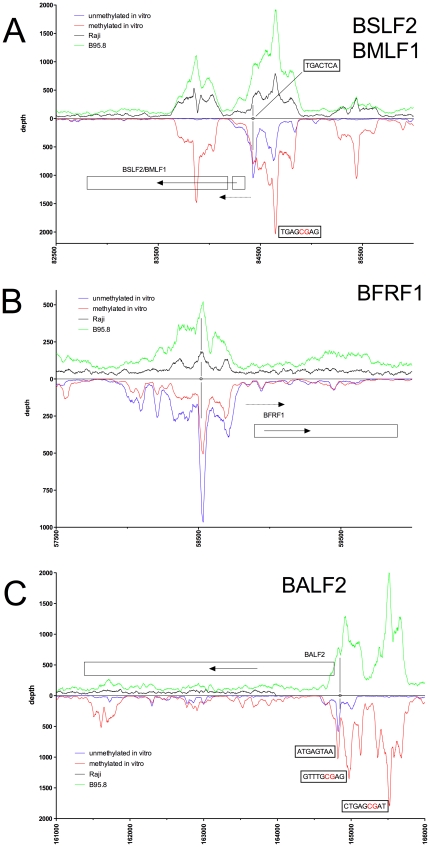
Identification of methylation-dependent Zta binding to selected viral promoter elements *in vivo* and *in vitro*. Deep sequencing data obtained from *in vitro* immunoprecipitation experiments with GFP:BZLF1 as in Figure 1 are plotted on selected genes and their promoters as indicated. EBV DNA free of CpG-methylation (blue) and after full methylation by the methyltransferase M.SssI (red) were used as probes in the experiments. *In vivo* ChIP-seq data obtained after immunoprecipitation of chromatin of Raji cells (black) or B95.8 cells (green) with GFP:BZLF1 are plotted. Ten genes, their exon compositions and the location of selected ZREs are shown. (A) The two annotated genes *BSLF2* and *BMLF1* are shown, which result in the spliced BMLF2/BSLF1 transcript encoding the viral SM protein also called EB2. (B) The *BFRF1* gene is shown. (C) The *BALF2* gene is shown, which encodes the major DNA binding protein of EBV.

**Figure 4 ppat-1001114-g004:**
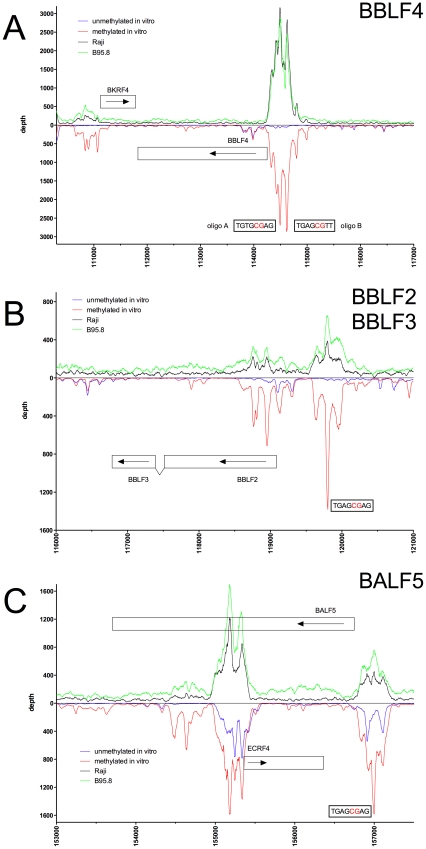
Identification of methylation-dependent Zta binding to selected viral promoter elements *in vivo* and *in vitro*. Deep sequencing data obtained from *in vitro* immunoprecipitation experiments with GFP:BZLF1 as in Figure 1 are plotted on selected genes and their promoters as indicated. EBV DNA free of CpG-methylation (blue) and after full methylation by the methyltransferase M.SssI (red) were used as probes in the experiments. *In vivo* ChIP-seq data obtained after immunoprecipitation of chromatin of Raji cells (black) or B95.8 cells (green) with GFP:BZLF1 are plotted. Ten genes, their exon compositions and the location of selected ZREs are shown. (A) The *BBLF4* gene encoding the viral DNA helicase and the *BKRF4* gene are shown. (B) The two open reading frames *BBLF2* and *BBLF3* are spliced and encode the primase-associated factor BBLF2/3. (C) The *BALF5* and the *ECRF4* genes are shown, which encode the viral DNA polymerase and a hypothetical protein, respectively.

**Figure 5:**
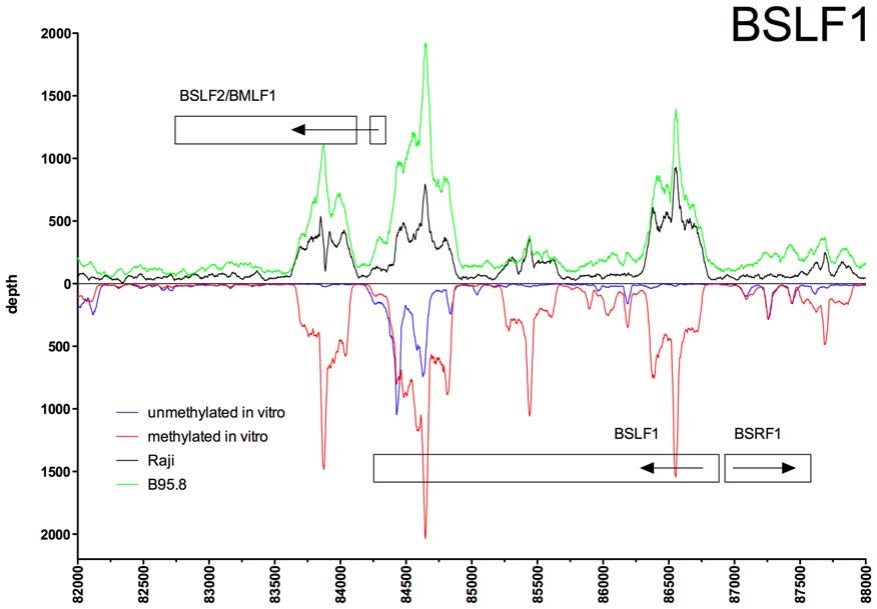
Identification of methylation-dependent Zta binding to the viral *BSLF1* and *BSLF2/BMLF1* promoter elements *in vivo* and *in vitro*. Deep sequencing data obtained from *in vitro* immunoprecipitation experiments with GFP:BZLF1 as in Figure 1 are plotted on selected genes and their promoters as indicated. EBV DNA free of CpG-methylation (blue) and after full methylation by the methyltransferase M.SssI (red) were used as probes in the experiments. *In vivo* ChIP-seq data obtained after immunoprecipitation of chromatin of Raji cells (black) or B95.8 cells (green) with GFP:BZLF1 are plotted. Ten genes, their exon compositions and the location of selected ZREs are shown. The loci of the *BSRF1* and *BSLF2/BMLF1* genes are shown in conjunction with the *BSLF1* gene, which encodes the viral primase.

In the *BRLF1* promoter (Figure 2B), two of the three previously published ZREs could be accurately determined [13], [25]. In its CpG-methylated version, ZRE2 of *BRLF1* showed an about fourfold increase in depth values confirming that this site is bound by Zta more strongly when methylated [13], [26]. ZRE3 of the *BRLF1* promoter, which was reported to bind Zta only in its CpG-methylated version (ibid), was not resolved (Figure 2B) but two additional and unreported potential ZREs were found upstream of ZRE2 in this promoter. The peaks of their depth values are set off by about 60 bp indicating that these potential sites do not colocalize and differ in their CpG-dependent binding characteristics.

Examples of our immunoprecipitation experiments shown in Figures 2C and 3 confirmed previously mapped single ZREs in the promoters of *BMRF1* [27], [28], *BSLF2/BMLF1* [9], [21], [29], *BFRF1* [30], and *BALF2* [31], [32] but they all contained additional, previously unidentified potential Zta binding sites, some of which were bound more strongly when methylated. These immunoprecipitations experiments did not confirm the recently published ZREs in the promoter of *BRRF1* [14] (Figure 2B).

The immunoprecipitation experiments also identified numerous additional Zta binding sites in the promoters of genes, which were not formerly known to be transactivated by Zta. For example, the promoters of *BSRF1* (Figure 5), *BKRF4* (Figure 4A), *BBLF4* (Figure 4A), *BBLF2/BBLF3* (Figure 4B), and *BALF5* (Figure 4C) were exclusively or preferentially enriched when CpG-methylated. The genes encode two different viral tegument proteins with unknown functions, the DNA helicase, the primase-associated factor, and the DNA polymerase of EBV, respectively. With the exception of *BSRF1* and *BKRF4*, these genes are directly or indirectly involved in lytic DNA amplification of virion DNA during productive infection, *BSLF2/BMLF1*, *BBLF4*, *BBLF2/BBLF3*, and *BALF5* being essential [33], [34]. Zta selectively targets a number of previously unknown methylated ZRE sites, for which we proposed the term meZRE [3], in the promoters of viral genes essential for EBV's lytic cycle. An extreme example is the *BBLF4* promoter, which Zta can only bind when it is CpG-methylated (Figure 4A).

### Chromatin immunoprecipitations reveal binding sites for Zta *in vivo*

Having found that Zta's binding to DNA is enhanced *in vitro* by methylation of that DNA we set out to test if Zta binds methylated DNA *in vivo*. We engineered a derivative of the human B-cell line Raji, which stably expressed a nuclear, chimeric GFP:BZLF1 protein (Supporting Figure S1B,C). Raji cells contain about 50 viral genomes per cell and are strictly latently infected with EBV. Chromatin immunoprecipitations (ChIPs) with the GFP-binding recombinant antibody [19] were performed and quantitative real-time PCR analysis confirmed the highly selective binding of GFP:BZLF1 to the *BBLF4* and *BMRF1* promoters as compared to a reference locus (terminal repeat, TR) and another Raji cell derivative, which expressed GFP:NLS as a control protein (Supporting Figure S1; Table S2 in Text S1). ChIP-seq allowed a direct comparison with our *in vitro* immunoprecipitations experiments (Figures 2 to [Fig ppat-1001114-g003] [Fig ppat-1001114-g004] 5). Identical ChIP-seq experiments were also performed with the B95.8 cell line, which is semipermissive for EBV's lytic phase (Figures 2 to [Fig ppat-1001114-g003] [Fig ppat-1001114-g004] 5).

Peak depth values in general coincided with those obtained *in vitro* with CpG-methylated EBV DNA as depicted in the promoters of *BMRF1*, *BSLF2/BMLF1*, *BBLF4*, *BBLF2/BBLF3*, and *BALF5* in Figures 2C, 3A, 4A, 4B, and 4C, respectively. The Raji EBV genome is highly CpG-methylated (Supporting Figure S3) and GFP:BZLF1 revealed a preferential binding to many promoters mimicking the patterns found with CpG-methylated EBV DNA in the immunoprecipitation experiments performed *in vitro*. In particular, Zta bound to the *BBLF4* promoter *in vivo* exactly recapitulating the depth profile seen with methylated EBV *in vitro* (Figure 4A).

A noticeable exception was the *BZLF1* promoter. *In vivo*, the two ZREs in the *BZLF1* promoter were bound by Zta in B95.8 cells, but not in Raji cells (Figure 2B) indicating that this promoter is regulated differently in the two cell lines. With the exception of the *BZLF1* promoter in Raji cells, Zta readily bound many viral lytic promoters *in vivo* indicating that that their chromatin configuration allowed access for Zta's DNA-binding moiety.

### Identification of consensus binding sites of Zta from ChIP-seq data

The coverage data of mapped reads shown in the panels of Figures 2 to [Fig ppat-1001114-g003] [Fig ppat-1001114-g004] 5 suggested the discrete localization of ZREs in the promoters of a number of viral genes. Although genomic regions enriched with mapped reads could be inferred as approximate binding sites, the fragment length of approximately 200 bp pose challenges for determining the exact protein-DNA binding site within these regions [35]. We therefore applied algorithms developed to delineate the exact localization of potential binding sites. Because the data in Figures 2 to [Fig ppat-1001114-g003] [Fig ppat-1001114-g004] 5 indicated that Zta's bind differently to unmethylated and CpG-methylated DNAs, we used the two data sets of our *in vitro* immunoprecipitation experiments to investigate whether specific consensus motifs can explain these differences. We first determined the protein binding sites with SISSRs (Site Identification from Short Sequence Reads [35]) and subsequently used these sites to identify statistically overrepresented consensus motifs within the inferred binding sites with the MEME (Multiple EM for Motif Elicitation) algorithm [36].

**Figure 6:**
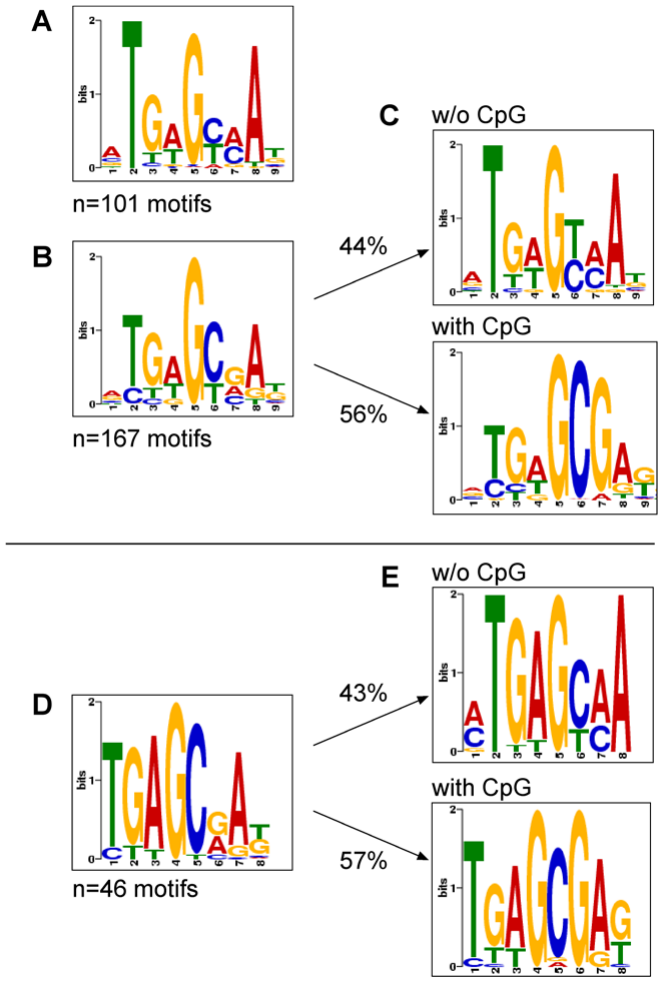
Motif discovery of Zta bound to unmethylated, CpG-methylated EBV DNA or Raji cell chromatin. ChIP-seq data and deep sequencing data after *in vitro* immunoprecipitation assays with GFP:BZLF1 were analyzed with the SISSRs (default parameters) or QuEST algorithms (kernel density estimate of 60, threshold value of 2) for putative stretches of DNA to which Zta binds. The outputs of SISSRs or QuEST were used as training sets for MEME, which identifies gapless, local, multiple sequence motifs [59]. (A) A total of 101 motifs were identified in *in vitro* immunoprecipitation experiments followed deep sequencing with E.coli-derived EBV DNA free of CpG methylation. (B) A total of 167 motifs were identified in *in vitro* immunoprecipitation experiments followed deep sequencing with fully CpG-methylated E.coli-derived EBV DNA. (C) The identified motifs in (B) were selected at the level of the SISSRs training set data and grouped into ZRE motifs with (bottom panel) and without (top panel) CpG dinucleotides followed by MEME analysis. (D) A total of 46 motifs were identified in ChIP-seq data after chromatin immunoprecipitations from Raji cells stably transfected with an expression plasmid encoding GFP:BZLF1 (Supporting Figure S1). (E) The identified motifs in (D) were selected at the level of the QuEST training set data and grouped into ZRE motifs encompassing no (top panel) or one or more (bottom panel) CpG dinucleotides. Subsequent MEME analysis identified two classes of ZREs.

SISSRs with standard settings was applied on *in vitro* ChIP-seq data for GFP:BZLF1-precipitated unmethylated and fully CpG-methylated EBV DNA. The analysis identified 126 and 200 potential peak calls, respectively, which tag the regions of high sequence read densities. We employed MEME to identify statistically overrepresented motifs within the selected peak calls representing inferred binding sites of Zta. MEME identified 101 and 167 sites in ChIP-seq data for GFP:BZLF1-precipitated unmethylated and fully CpG-methylated EBV DNAs, which fulfilled the criteria for canonical Zta-binding motifs and are depicted in Figure 6A and B, respectively, and listed in Supporting Data S1. The consensus of the motifs obtained with CpG-methylated EBV DNA contained one obvious (position 6 and 7) and several less frequent, putative C-G dinucleotide pairs (Figure 6B). We therefore conducted a second round of MEME analysis focusing only on those peak-associated sequences with CpGs. The separate analysis yielded 85 peak calls (56%) with a second consensus Zta recognition motif, in which a reliable and prominent C-G pair was evident (Figure 6C, bottom panel) in marked contrast to the motif shown in Figure 6A and 6C, top panel, which lacks any C-G dinucleotide.

We analyzed the ChIP-seq data obtained with GFP:BZLF1 immunoprecipitations of Raji and B95.8 cell chromatin with the QuEST (kernel density estimate of 60, threshold value of 2) [37] and SISSRs algorithms (default parameters). Subsequent MEME analysis of a total of 46 identified peak calls yielded a consensus motif in Raji cells (Figure 6D) reminiscent of the corresponding motif in Figure 6B. A second round of analysis identified 20 consensus motifs free of CpG pairs (Figure 6E, top panel) and very similar to the one in Figure 6A. The remaining 26 motifs contained a consensus motif with CpG dinucleotides (Figure 6E, bottom panel), which was almost indistinguishable from the one shown in Figure 6C, bottom panel. Almost identical results were obtained in our studies with B95.8 cell chromatin (Supporting Figure S4).

Taken together, *in vivo* ChIPs with GFP:BZLF1 confirmed our initial *in vitro* findings in immunoprecipitation experiments. Both strategies independently identified two classes of putative Zta binding sites. T-G-A-G-C/T-A/C-A (Figure 6E, top panel) lacks any CpG pair and stands for several previously identified Zta binding sites. The more frequent second class of ZREs is T-G-A-G-C-G-A-G/T (Figure 6E, bottom panel), contains a consistent CpG dinucleotide (underlined), is present in the ZRE2 site (T-G-A-G-C-G-A-T) of the *BRLF1* promoter [13], [25] but has not been identified elsewhere.

Surprisingly, our analysis did not identify the previously proposed sequences of the ZRE3 site (T-C-G-C-G-A-A) in the *BRLF1* promoter [13], [15], of the distal ZRE site (T-C-G-C-T-C-C) in the cellular *egr1* gene [38], or of the two ZREs in the *BRRF1* promoter (T-G-A-G-C-G-T-G and T-C-G-C-C-C-G-T) [14], which have been proposed to bind Zta when methylated.

### Zta binds preferentially to CpG-methylated viral lytic promoters

**Figure 7:**
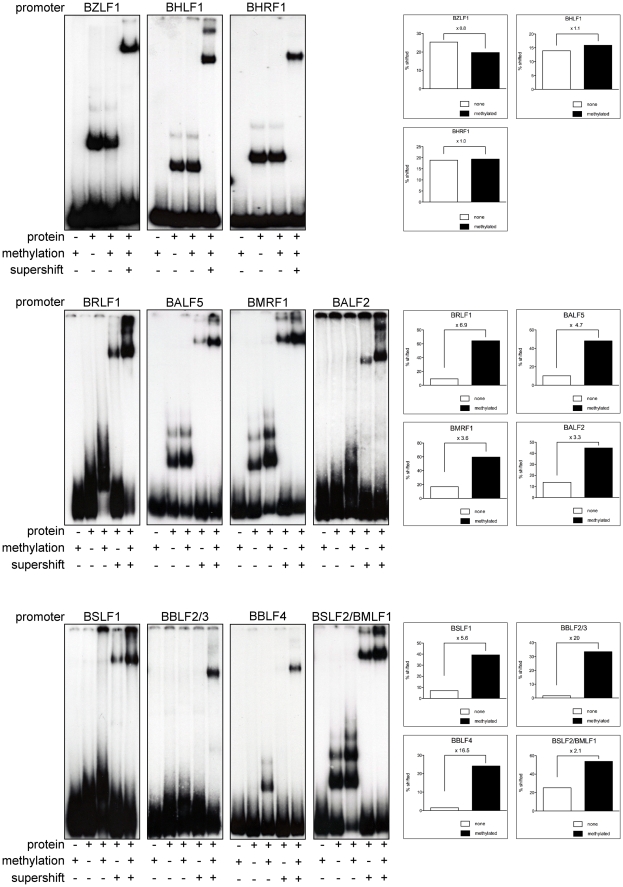
Zta binds preferentially to CpG-methylated promoters. In EMSAs, Zta bound preferentially to the CpG methylated promoters of *BRLF1*, *BALF2*, *BSLF2/BMLF1*, *BALF5* and *BMRF1* and *BSLF1* but it bound only the CpG-methylated promoters of *BBLF4* and *BBLF2/3*. CpG methylation did not influence Zta binding to the promoters of *BZLF1*, *BHLF1*, and *BHRF1*. EMSAs were performed with affinity-purified Strep/FLAG-tagged BZLF1 fusion protein transiently expressed in HEK293 cells. Eleven PCR fragments encompassing EBV promoters with BZLF1 binding sites identified in Figures 2 to [Fig ppat-1001114-g003] [Fig ppat-1001114-g004] 5 served as radioactive probes. PCR fragments (Table S3 in Text S1) were used either unmethylated or fully CpG-methylated *in vitro* with M.SssI. Supershifts with a FLAG-antibody confirmed the identity of the protein-DNA complexes. The signals were scanned and the percent ratios of bound DNAs versus total input DNAs are shown for each sample.

The results in Figures 2 to [Fig ppat-1001114-g003] [Fig ppat-1001114-g004] 5 suggested that Zta binds to a number of promoters of lytic viral genes when CpG-methylated. We used electrophoretic mobility shift assays (EMSAs) to re-evaluate whether purified BZLF1 protein expressed in HEK293 cells can directly bind to viral promoter sequences in a CpG-methylation-dependent fashion. We examined the promoters of eleven genes (Table S3 in Text S1), which are all indispensable for EBV's lytic phase. Zta bound to the promoters of the *BZLF1*, *BHLF1*, and *BHRF1* genes irrespective of their status of CpG-methylation but CpG methylation of eight promoters increased Zta's binding to DNA two- to 20fold (Figure 7). *BRLF1* encodes the immediate early transcription factor Rta, which was previously found to be regulated in a methylation-dependent fashion [13]. The promoter of the early *BSLF2/BMLF1* gene encoding the viral RNA export factor SM is also preferentially bound by Zta when methylated. The remaining six genes with this characteristic are *BBLF4*, *BALF5*, *BMRF1*, *BALF2*, *BSLF1*, and *BBLF2/3*, which are early viral genes essential for replication of viral DNA and encode the viral helicase, DNA polymerase, DNA polymerase processivity factor, DNA binding protein, primase, and primase-associated factor, respectively.

### Zta transactivates methylated promoters of essential viral genes

**Figure 8:**
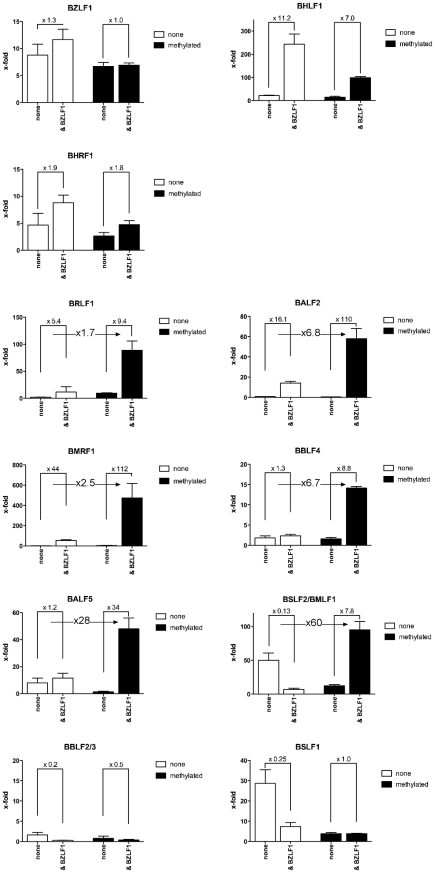
Zta transactivates CpG-methylated promoters with superior efficiency. Promoters of eleven different genes (Table S3 in Text S1) were inserted into a synthetic luciferase reporter plasmid free of CpG-dinucleotides [39]. Reporter plasmids were either used after isolation from E.coli and thus free of methylated CpG dinucleotides (unmethylated, white) or after complete *in vitro* CpG-methylation (black) and co-transfected with a BZLF1 expression plasmid or with a negative control DNA as indicated (none). After data normalization to a luciferase control plasmid free of promoter elements, the x-fold differences in the different data sets were calculated as shown.

We analyzed the promoters of lytic viral genes in luciferase reporter assays (Figure 8) with the aid of a synthetic reporter plasmid devoid of CpG dinucleotides to study the effect of CpG-methylation on the inserted promoter fragments [39]. In these experiments, the reporter plasmid DNAs were isolated from E.coli and did not carry methylated CpG dinucleotides. *In vitro* CpG-methylation by the *de novo* methyl transferase M.SssI limited cytosine methylation to the inserted promoter element. Eleven different reporter plasmids, either unmethylated or fully methylated *in vitro*, were transiently transfected in HEK293 cells and their luciferase activities were measured. The results shown in Figure 8 revealed that Zta transactivates six viral promoters as a function of their CpG methylation.

The *BRLF1* promoter was only moderately responsive to CpG methylation (1.7fold) as published previously [13], but CpG methylation of the promoters of five genes, *BALF2*, *BMRF1*, *BBLF4*, *BALF5* and *BSLF2/BMLF1* enhanced luciferase activity up to 60fold. The *BSLF2/BMLF1* promoter was reported to be responsive to Zta expression [21], [29], [40] but in our experiments the unmethylated promoter was repressed by Zta in HEK293 cells, which might be due to putative negative regulatory regions in the *BSLF2/BMLF1* promoter [41] in line with another earlier report [42]. Zta did not detectably transactivate the promoters of two genes, *BBLF4* and *BALF5* when unmethylated, but CpG methylation activated them by a factor of 6.7 and 28, respectively. For unknown reasons, the *BSLF1* and *BBLF2/3* promoters were unresponsive to Zta in the luciferase assays although it preferentially bound to their DNAs when methylated (Figures 4B, 7). Induced expression of Zta in Raji cells led to dramatic upregulation of all these genes (Table S4 in Text S1) as shown by quantitative RT-PCR analyses (Supporting Figure S5).

### Functional identification of single meZREs

**Figure 9:**
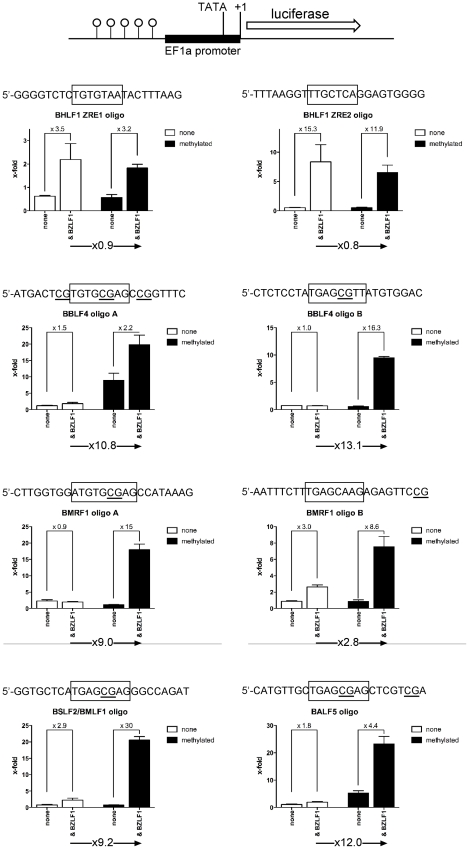
Functional identification of single ZREs and meZREs. Pentamers of ten single ZREs present in the promoters of *BHLF1*, *BBLF4*, *BMRF1*, *BSLF2/BMLF1*, and *BALF5* were introduced into a basic luciferase reporter plasmid with a minimal EF1α promoter and free of CpGs [39]. Unmethylated and fully CpG methylated reporter constructs were analyzed in the presence or absence of a co-transfected *BZLF1* expression plasmid.

Ten inferred Zta binding sites (Figures 6, 7; Supporting Figure S4) of five different promoters (*BHLF1*, *BBLF4*, *BMRF1*, *BSLF2/BMLF1*, and *BALF5*) were analyzed as pentamers in a basic luciferase reporter plasmid free of CpGs [39] in their unmethylated and fully CpG-methylated states. CpG methylation of meZREs led to a nine to 13-fold activation of luciferase (Figure 9) indicating that Zta indeed binds to the newly identified meZREs motifs and activates transcription in a methylation-dependent manner in the promoters of *BBLF4*, *BMRF1*, *BSLF2/BMLF1*, and *BALF5*. As expected, CpG methylation of ZREs that do not include CpGs did not stimulate luciferase activity as exemplified by the *BHLF1* promoter (Figure 9). In EMSAs, the fraction of Zta-bound oligonucleotides with selected, single ZREs was measured as a function of protein concentration (Supporting Figure S6). The affinity of Zta transiently expressed in and purified from HEK293 cells was determined for the different ZREs (Table S5 in Text S1) fitting the data to the Hill equation [43]. In general, Zta protein bound to CpG-methylated meZREs with considerably higher affinities (K_d, app_∼12 to 16nM) than to conventional ZREs (K_d, app_∼40 to 120nM) [44] or unmethylated meZREs (K_d, app_∼>200nM) (Table 1). Interestingly, the heterodimeric Fos-Jun and the homodimeric CREB proteins, members of the AP-1 family, were reported to bind to their cognate recognition motifs with comparable affinities (10.8nM and 5nM, respectively) and in the range of meZREs [45], [46].

**Table 1:** Binding affinities of Zta to selected unmethylated and CpG-methylated ZREs.

ZRE	EBV coordinates*	Binding motif (CpG underlined)	K_d app_ [nM] methylated mean ± SD	K_d app_ [nM] unmethylated mean ± SD
BBLF4 ZRE A	114487:114511	TGTGCGAG	16.3±2.6	219±41
BBLF4 ZRE B	114609:114633	TGAGCGTT	18.0±4.2	484±244
BMRF1 ZRE A	79611:79635	ATGTGCGAG	20.2±12.5	328±166
BRLF1 ZRE2	106363:106387	TGAGCGA	12.7±8.2	113±11.9
BRLF1 ZRE3	106423:106447	TTCGCGA	135±45	1189±227
oriLyt ZRE5	53462:53486	TTGCACA	nd	37.7±4.3
BMLF1 ZRE AP-1	84420:84444	TGACTCA	nd	40.8±23.0
BHLF1 ZRE2	52844:52867	TTGCTCA	nd	91.6±38.5

*EBV B95.8 strain; nd: not done.

## Discussion

Our data identify the structure and function of novel DNA motifs, which Zta binds with increased affinities or even exclusively when CpG-methylated. As revealed in our genome-wide search (Figure 1 and data not shown) more than 20 viral early genes are potentially regulated by Zta via meZREs. Promoters such as *BBLF4* and *BALF5* (Figures 4A, 7) are bound and transactivated by Zta, only, when their meZREs are CpG-methylated indicating a newly appreciated level of regulation for the onset of EBV's lytic productive phase. Among others, these genes constitute an essential class of viral proteins indispensable for EBV's lytic DNA replication. EBV DNA is unmethylated upon infection, and this initial state of viral DNA insures an efficient block of EBV's lytic phase preventing the onset of virus production and presumably cellular death of the newly infected cell. The failure of Zta to bind to unmethylated viral DNA and transactivate essential viral lytic genes shortly after infection therefore appears as a prerequisite for stable latent infections of primary B cells because *BZLF1* is expressed immediately after infection [3], [47]. Promoters of *BBLF4* and *BALF5* among other essential lytic genes become responsive to Zta-mediated transcriptional activation only later after *de novo* methylation in latently infected cells [3].

The class of DNA sequences, which Zta binds only when CpG-methylated, is without precedent and suggests an ingenious bypass to overcome the restricted state of epigenetic repression of silenced chromatin with CpG-methylated DNA. Remarkably, Zta can gain access to and bind to these meZREs *in vivo* despite the strictly repressed and heavily CpG-methylated chromatin of EBV DNA (Supporting Figures S3, S5; Figures 2 to [Fig ppat-1001114-g003] [Fig ppat-1001114-g004] 5; data not shown). GFP:BZLF1 encompasses Zta's DNA binding and dimerization domains, only, suggesting that Zta's binding to DNA is independent of active chromatin remodeling and/or histone modifications *in vivo* (Figures 2 to 5; Supporting Figure S2). It is likely that the transactivation domain of full length Zta induces these modifications to facilitate transcription because Zta interacts with CBP [48], [49]. Promoters with meZREs are silent in Raji cells but readily accessible to Zta's binding and poised for expression (Supporting Figure S5), a situation reminiscent of the bivalent marks in chromatin of embryonic stem cells during developmental differentiation [50]–[52].

Understanding how Zta binds meZREs with increased affinities summarized in Table 1 is fundamentally important. Zta is a member of the AP-1 transcription factor family [9], which in marked contrast to many AP-1 family members binds non-palindromic recognition elements as a homodimer (similar to homodimeric Jun-Jun). Zta can also bind to the classical, symmetric AP-1 consensus T-G-A-C/G-T-C-A (Table 1). The crystal structure of this complex was solved [53], which, however, does not resolve the riddle of how Zta binds with similar affinities to asymmetric recognition motifs of conventional ZREs and to meZREs with even higher affinities when methylated [15]. In fact, heterodimeric Fos-Jun and ATF2-Jun complexes exhibit orientation-dependent preferences in binding to AP-1 sites [54] suggesting non-identical recognition of the two half sites of the AP-1 recognition sequence by Fos, ATF2 and Jun. Jun binds preferentially to the consensus at asymmetric recognition sequences whereas Fos binds to the non-consensus half site affecting transactivation and cooperativity with other transcription factors such as NF-AT [55]. How Zta binds to non-palindromic sequences remains to be addressed experimentally but the structural similarities of homodimeric Zta and heterodimeric Fos-Jun when bound to DNA [53], [56] support a model in which certain members of the AP-1 transcription factor family also ‘read’ DNA sequences in a methylation-dependent manner. In this model Zta would be the founding example in a mechanism that directly promotes transcriptional regulation of repressed cellular chromatin.

## Materials and Methods

### Cells

HEK293, B95.8 and Raji cells have been described and were maintained in RPMI medium with 10% fetal calf serum (FCS), 1% penicillin-streptomycin and 1% sodium-pyruvate at 37°C and 5% CO_2_.

### Plasmids

The wild-type maxi-EBV plasmid (p2089) encompassing the complete genome of the EBV prototype B95.8 strain and the *BZLF1* expression plasmid have been described. The GFP:BZLF1 expression plasmid p3927 was described recently [3]. The GFP:NLS expression plasmid p4247 served as a control and contains the nuclear localization signal from SV40 T-antigen (amino acids PKKKRKVG) cloned in phase with the GFP gene expressed from the CMV promoter in pEGFP-C1 (Clontech). The BZLF1:Strep/FLAG expression plasmid (p3928) contains amino acids 149–245 of BZLF1 cloned in phase with a tandem StrepII/FLAG-tag [57]. Luciferase plasmids were constructed by PCR amplifying the promoter fragments of interest (Table S3 in Text S1) from B95.8 genomic EBV-DNA and inserted upstream of the CpG-basic vector described [39].

### Stable transfection and establishment of cell lines

5 µg DNA of either p3927 (GFP:BZLF1) or p4247 (GFP:NLS) DNA was transfected into 5×10^6^ cells by electroporation in 250 µl Optimem (Invitrogen) at 230 V and 975 µF using a Biorad electroporation apparatus in 4mm cuvettes. Immediately after electroporation, cells were resuspended in 400µl FCS and 10 ml growth medium was added. Cells were incubated in tissue culture flask at 37°C and 5% CO2 for two days. Afterwards cells were plated into 96-well plates in 200µl growth medium supplemented with G418 (1.5 mg/ml for Raji cells and 1.0 mg/ml for B95.8 cells). Within the next 4–5 weeks, G418 resistant cells grew out. When an appropriate cell density was reached, cells were expanded successively into 48 and 24 well plates. Cells were maintained in culture under G418 selection.

### DNA transfection

DNA transfections into HEK293 cells were performed using polyethylenimine (PEI) (Sigma-Aldrich). Transfection mixtures and transfection reactions were prepared as recently described [3]. For protein extracts 5×10^6^ cells per 15mm dish were seeded the day prior to transfection. Each plate was transfected with 15µg BZLF1:Strep/FLAG (p3928). For reporter assays 3.5×10^5^ HEK293 cells were seeded into six-well cluster plates the day before transfection. Each well was co-transfected with 1µg of reporter plasmid together with 0.5µg transactivator (p509) and 0.05µg renilla plasmid DNA as an internal control for data normalization.

### *In vitro* immunoprecipitation assays with GFP:BZLF1

The DNA binding and dimerization domain of BZLF1 (amino acid residue #149 to #245) was cloned in phase downstream of the coding region of eGFP in pEGFP-C1 (Clontech) to generate the expression plasmid p3927.1. This plasmid was transiently transfected into HEK293 cells, which expressed the GFP:BZLF1 chimera at high levels as a nuclear protein. The GFP:BZLF1 protein was purified from the cells and *in vitro* immunoprecipitation assays with E.coli-derived genomic EBV DNA were performed as described in detail [3].

### Native chromatin immunoprecipitations (ChIP)

5×10^7^ Raji cells stably transfected with GFP:BZLF1 were collected and washed 3 times with ice cold PBS. Cells were resuspended in a hypotonic buffer (10mM Hepes, 10mM KCl, 340mM sucrose, 1.5mM MgCl_2_, pH 7.9) and incubated on ice for 15min. Nuclei were prepared by adding Triton-X 100 (Sigma) to the swollen cells at a final concentration of 0.1%. Nuclei were collected by centrifugation and lysed with RIPA buffer (50mM Tris, 150mM NaCl, 1% NP40, 0.5% DOC, 0.1% SDS, ph 8.0) for 15 minutes on ice and chromatin was sheared by sonication to an average size of 250–350 bp. DNA-bound GFP:BZLF1 was immunoselected and immobilized with a GFP-Nanotrap reagent at 4°C over night. The immunocomplex was washed with low salt buffer (0.1% SDS, 1% Triton X-100, 2mM EDTA, 20mM Tris-HCl, pH 8.1, 150mM NaCl), high salt buffer (0.1% SDS, 1% Triton X-100, 2mM EDTA, 20mM Tris-HCl, pH 8.1, 500mM NaCl) and TE-buffer (10mM Tris, 0.5mM EDTA, pH 8.0). The beads were resuspended in elution buffer (10mM Tris, 0.5 mM EDTA, 2% SDS, pH 8.0 containing 20µg proteinase K) and incubated at 65°C for 2h. Protein-free DNA was phenol extracted, precipitated and analyzed by real-time PCR or ChIP-seq.

### Electromobility shift assays (EMSA)

EMSAs were performed with purified protein from HEK293 cells, transiently transfected with BZLF1:Strep/FLAG (p3928). Two days post transfection, cells from eight 15mm plates were pooled and lysed in 10ml RIPA-buffer (50mM Tris, 150mM NaCl, 1% NP40, 0.5% DOC, 0.1% SDS, pH 8.0). Cell lysates were sonicated and BZLF1:Strep/FLAG was column affinity purified with Strep-Tactin sepharose as described by the manufacturer (iba-biotagnology). BZLF1:Strep/Flag was eluted in 500µl Strepelution buffer. For each EMSA reaction 1 µl of a 1∶10 dilution of purified protein was incubated with 5000cpm of radioactive labeled probe in the presence of 20mM Hepes, 75mM NaCl, 10% glycerin, 2µg polydIdC (Roche), 2mM MgCL_2_ 100ng calf thymus DNA and 0.1mg/ml BSA. EMSA probes were created by PCR amplification to generate promoter specific large probes (Table S3 in Text S1). Oligonucleotides containing single ZREs were provided by Sigma-Aldrich or Metabion in a CpG-methylated or an unmethylated form (Table S5 in Text S1). Probes were ^32^P end-labeled with T4 polynucleotide kinase (Promega) in accordance with the manufacturer's instructions. For Kd-value calculation annealed double-stranded oligonucleotides (250pM) were incubated with twofold serial dilutions of purified BZLF1:Step/Flag protein for 30 minutes. Unbound DNA was separated from Protein-DNA complexes by polyacrylamide gel electrophoresis (12% (w/v) 29∶1 acrylamide/bisacrylamide, 0.5× TBE). Gels were scanned using a phosphoimager (FLA 5100, Fuji) and the ratio of the free and bound DNA was calculated. These data were fit to the Hill equation with one site specific binding using the Prism 5 (graphpad.com) software to determine the dissociation constant.

### *In vitro* methylation

Luciferase reporter constructs and PCR fragments (EMSAs) were methylated *in vitro* with the *de novo* methyltransferase M.SssI and S-adenosyl methionine (SAM) as the methyl donor. 15µg plasmid DNA or 5µg PCR-product were incubated with 40U M.SssI (New England Biolabs) and 1µl SAM (32mM stock concentration) in 150µl H_2_O buffered with NEB buffer 2 at 37°C over night. The next day the reaction was again supplied with 1µl SAM and further incubated for 2 hours. Complete CpG-methylation of plasmids and PCR fragments was confirmed by digestion with the methylation-sensitive restriction enzyme *HpaII* (New England Biolabs).

### Luciferase reporter assays

48 hours post transfection the HEK293 cells were analyzed with the Dual-Luciferase Reporter Assay System (Promega). Luciferase activity was measured in a 96-well microplate luminometer (Orion II, Berthold).

### Library construction

Sequence libraries were constructed with paired-end DNA sample preparation kits (Illumina) according to the manufacture's recommendations with minor modifications. Briefly, 500ng of double-stranded DNA fragments were converted to blunt ends using T4 DNA polymerase and Klenow polymerase. Subsequently a single adenine base was added to the DNA using Klenow exo- (3′ to 5′ exo minus). Next, paired end DNA adaptors with a single thymidine base overhang at the 3′ end were ligated to the above products. The adaptor-modified DNA fragments were then separated on a 2% low melting agarose gel (Biozym), a 300+/−25 bp DNA band was excised from the gel and purified (Qiagen Gel Extraction Kit). 12 cycles of PCR enrichment with paired end PCR primers 1.0 and 2.0 (Illumina) were performed. The purified (Qiagen MinElute PCR purification kit) libraries were quantified using an Agilent bioanalyzer. Library hybridization to the flow cells and cluster generation was performed on an Illumina cluster station following the manufactures protocols (Illumina paired-end cluster generation kit GAII v1, 36-cycle sequencing kit v1).

### Read mapping

Sequencing was performed on an Illumina Genome Analyzer IIx. *In vitro* immunoprecipitations with unmethylated and fully CpG-methylated EBV DNAs were converted into the libraries ‘44466_unmethylated’ and ‘44467_methylated’, respectively. We generated 6.13 million and 3.78 million 36-bp single-end reads for libraries ‘44466_unmethylated’ and ‘44467_methylated’, respectively. DNA obtained with ChIP of Raji cell chromatin was converted into the library ‘46312_Raji_BZLF1’, which was analyzed with 6.13 million 36-bp paired-end reads.

Read mapping to the reference sequence and subsequent assembly was performed using the re-sequencing software MAQ (v0.7.1; [58]). We used a combination of the EBV (recombinant EBV strain 2089) [18] and the bovine genome (build bosTau4) as reference sequences for the two libraries ‘44466_unmethylated’ and ‘44467_methylated’. (Calf thymus DNA was used as an unspecific competitor in the *in vitro* immunoprecipitation assays with GFP:BZLF1.) The reference sequence for the *in vivo* sample was composed of the recombinant 2089 EBV genome and the human reference sequence (build hg18). Approximately 83–87% of the reads mapped to reference sequences. Duplicated reads of the library ‘46312_Raji_BZLF1’ (0.55%), as defined by identical outer coordinates of mate-pair reads, were removed.

### Transcription factor binding site detection

Genomic regions with a read depth above the background level are considered as transcription factor binding sites. We used two different programs, SISSRs [35] and QuEST [37] to identify these regions. Transcription factor binding sites are typically shorter than the sequenced DNA fragments. Both programs employ methods to infer the position of the binding sites within the reads from the densities of the forward and reverse reads.

The SISSRs algorithm uses the read direction (sense or antisense strand), density of reads and the average DNA fragment length to identify potential binding sites. The genome is therefore scanned in a sliding window approach using a window size of 20 bp (default) with consecutive windows overlapping 10 bp. For each window the net tag count, defined as the number of reads on the sense strand subtracted by the number of reads on the antisense strand, is computed. The algorithm then identifies transitions from positive to negative net tag count and records the transition points as potential binding site, which needs to satisfy further criteria to be considered a true binding site. These further criteria include threshold values for the individual positive and negative net tag counts as well as a threshold for the combined count of positive and negative tags. This combined count, referred to as number of directed reads supporting the site, is then also interpreted as score to rank binding sites.

Binding sites in this study were inferred using the default SISSRs parameters.

The second algorithm employed for peak calling is QuEST [37]. Similar to SISSRs, QuEST uses read counts from forward and reverse genomic strands as well as the average fragment length as information, yet they differ in several key points. QuEST's statistical framework is based on the kernel density approach to aggregate the signal originating from densely clustered reads at potential binding sites. As only the first 36 base pairs of each DNA fragment get sequenced, forward and reverse strand reads accumulate on opposite sides, leading to an under-representation of reads at the actual binding site. To cope with this, QuEST constructs two density profiles, one for forward and one for reverse reads, then combines both profiles in a procedure termed “peak-shift”. The actual distance of the peak-shift is defined as half the distance between forward and reverse profile and may vary between different experiments depending on the DNA fragment length distribution.

After determining the experiment-specific peak-shift, both density profiles are shifted accordingly and summed to produce a combined density profile, which is employed in all further analysis. Actual binding sites are determined by scanning the combined density profiles for local maxima with sufficient enrichment when compared to the background model or control data. The output of QuEST is a list of all potential binding sites with genomic coordinates, ranked according to their kernel density estimation-derived scores.

In order to identify consensus motifs within the discovered binding sites, the sequences around the binding sites were extracted and used as input for the motif finding algorithm MEME [36]. Default parameters were used for MEME.

## Supporting Information

Text S1 Supporting Tables S1 to S5, figure legends for Supporting Figures S1 to S6.
(0.13 MB DOC)

Figure S1 Analysis of GFP fusion proteins stably expressed in Raji cell lines.
(6.84 MB PDF)

Figure S2 GFP:BZLF1 binds sequence-specifically to selected EBV promoters *in vivo*.
(0.02 MB PDF)

Figure S3 MeDIP (Methylated DNA Immunoprecipitation) analysis indicates a high degree of CpG methylation of genomic EBV DNA in Raji cells.
(0.19 MB PDF)

Figure S4 Motif discovery of Zta binding to B95.8 DNA in ChIP-seq data. ChIP-seq data were selected via the SISSRs algorithms (default parameters) [3] and the output was used as the training set for MEME (Multiple EM for Motif Elicitation), which identifies gapless, local, multiple sequence motifs [4]. (A) A total of 39 motifs were identified shown as a consensus logo motif in the unselected SISSRs data training set. (B) The identified motifs in (A) were selected at the level of the SISSRs training set data and grouped into ZRE motifs with (bottom panel) and without (top panel) CpG dinucleotides followed by MEME analysis.
(1.18 MB PDF)

Figure S5 BZLF1 induces expression of genes essential for viral replication *in vivo*. A conditional expression plasmid [5] encoding a tetracycline-regulated *BZLF1* allele (p3862) was stably introduced into Raji cells. Total cellular RNAs were isolated before (−dox) and twelve hours after addition of doxycyclin (+dox). After reverse transcription relative levels of selected viral transcripts were assessed by quantitative real-time PCRs, which were normalized to the constitutive transcripts level of the housekeeping cytochrome c (*cyt*) gene.
(0.03 MB PDF)

Figure S6 EMSA quantification of the fraction of selected Zta-bound unmethylated und methylated ZRE oligonucleotides and determination of K_d app_. In EMSAs, the fraction of Zta-bound oligonucleotides with selected, single ZREs was measured as a function of protein concentration and the Kd values of Zta and different unmethylated and CpG-methylated ZREs were determined as described [6]. EMSAs of typical experiments are shown as examples.
(1.55 MB TIF)

Supporting Data S1 Training sets and MEME output files.
(0.44 MB DOC)
