# Supplementary material for: CpG-Methylation Regulates a Class of Epstein-Barr Virus Promoters
Source: PLoS Pathog. 2010 Sep 23;6(9):e1001114. doi: 10.1371/journal.ppat.1001114 (PMC2944802; doi:10.1371/journal.ppat.1001114)
Supplement: Text S1 — Supporting Tables S1 to S5, figure legends for Supporting Figures S1 to S6. (0.13 MB DOC) [file ppat.1001114.s001.doc]

CpG-Methylation Regulates a Class of Epstein-Barr Virus’ Promoters

Martin Bergbauer^1^, Markus Kalla^1^, Anne Schmeinck^1^, Christine Göbel^1^, Ulrich Rothbauer^2^, Sebastian Eck^3^, Anna Benet-Pagès^3^, Tim M. Strom^3^, and Wolfgang Hammerschmidt^1^*

**SUPPORTING FILES**

^1^Department of Gene Vectors

Helmholtz Zentrum München

German Research Center for Environmental Health

Marchioninistr. 25

D-81377 Munich, Germany

^2^Biocenter at the Department of Biology II

Ludwig-Maximilians University Munich

Grosshaderner Str. 2

D-82152 Martinsried, Germany

^3^Institute of Human Genetics

Helmholtz Zentrum München

German Research Center for Environmental Health

Ingolstädter Landstr. 1,

D-85764 Neuherberg, Germany

*Corresponding author

Wolfgang Hammerschmidt

Phone: +49-89-3187-1506

Fax: +49-89-3187-19-1506

E-mail: hammerschmidt@helmholtz-muenchen.de

**Supporting Table S1: Overview of Zta’s known binding sites in EBV**

| Promoters with ZREs | References |
| --- | --- |
| BZLF1 | Urier, G., Buisson, M., Chambard, P., and Sergeant, A. (1989). The Epstein-Barr virus early protein EB1 activates transcription from different responsive elements including AP-1 binding sites. EMBO J *8*, 1447-1453.  Flemington, E., and Speck, S. H. (1990). Autoregulation of Epstein-Barr virus putative lytic switch gene BZLF1. J Virol *64*, 1227-1232. |
| BRLF1 | Packham, G., Economou, A., Rooney, C. M., Rowe, D. T., and Farrell, P. J. (1990). Structure and function of the Epstein-Barr virus BZLF1 protein. J Virol 64, 2110-2116.  Bhende, P. M., Seaman, W. T., Delecluse, H. J., and Kenney, S. C. (2004). The EBV lytic switch protein, Z, preferentially binds to and activates the methylated viral genome. Nat Genet *36*, 1099-1104. |
| BMLF1/BSLF2 | Rooney, C. M., Rowe, D. T., Ragot, T., and Farrell, P. J. (1989). The spliced BZLF1 gene of Epstein-Barr virus (EBV) transactivates an early EBV promoter and induces the virus productive cycle. J Virol *63*, 3109-3116.  Farrell, P. J., Rowe, D. T., Rooney, C. M., and Kouzarides, T. (1989). Epstein-Barr virus BZLF1 trans-activator specifically binds to a consensus AP-1 site and is related to c-fos. EMBO J *8*, 127-132.  Urier, G., Buisson, M., Chambard, P., and Sergeant, A. (1989). The Epstein-Barr virus early protein EB1 activates transcription from different responsive elements including AP-1 binding sites. EMBO J *8*, 1447-1453.  Buisson, M., Manet, E., Trescol, B. M., Gruffat, H., and Durand, B. (1989). The Epstein-Barr virus (EBV) early protein EB2 is a posttranscriptional activator expressed under the control of EBV transcription factors EB1 and R. J Virol *63*, 5276-5284. |
| BHLF1/BHRF1 | Lieberman, P. M., Hardwick, J. M., Sample, J., Hayward, G. S., and Hayward, S. D. (1990). The zta transactivator involved in induction of lytic cycle gene expression in Epstein-Barr virus-infected lymphocytes binds to both AP-1 and ZRE sites in target promoter and enhancer regions. J Virol *64*, 1143-1155.  Chavrier, P., Gruffat, H., Chevallier-Greco, A., Buisson, M., and Sergeant, A. (1989). The Epstein-Barr virus (EBV) early promoter DR contains a cis-acting element responsive to the EBV transactivator EB1 and an enhancer with constitutive and inducible activities. J Virol *63*, 607-614. |
| BMRF1 | Kenney, S. C., Holley-Guthrie, E., Quinlivan, E. B., Gutsch, D., Zhang, Q., Bender, T., Giot, J. F., and Sergeant, A. (1992). The cellular oncogene c-myb can interact synergistically with the Epstein-Barr virus BZLF1 transactivator in lymphoid cells. Mol Cell Biol *12*, 136-146.  Quinlivan, E. B., Holley-Guthrie, E. A., Norris, M., Gutsch, D., Bachenheimer, S. L., and Kenney, S. C. (1993). Direct BRLF1 binding is required for cooperative BZLF1/BRLF1 activation of the Epstein-Barr virus early promoter, BMRF1. Nucleic Acids Res *21*, 1999-2007. |
| BALF2 | Hung, C. H., and Liu, S. T. (1999). Characterization of the Epstein-Barr virus BALF2 promoter. J Gen Virol *80*, 2747-2750.  Nakayama, S., Murata, T., Murayama, K., Yasui, Y., Sato, Y., Kudoh, A., Iwahori, S., Isomura, H., Kanda, T., and Tsurumi, T. (2009). Epstein-Barr Virus Polymerase Processivity Factor Enhances BALF2 Promoter Transcription as a Coactivator for the BZLF1 Immediate-Early Protein. J Biol Chem *284*, 21557-21568. |
| F promoter | Zetterberg, H., Jansson, A., Rymo, L., Chen, F., Karlsson, A., Klein, G., and Brodin, B. (2002). The Epstein-Barr virus ZEBRA protein activates transcription from the early lytic F promoter by binding to a promoter-proximal AP-1-like site. J Gen Virol *83*, 2007-2014. |
| BFRF1 | Granato, M., Farina, A., Gonnella, R., Santarelli, R., Frati, L., Faggioni, A., and Angeloni, A. (2006). Regulation of the expression of the Epstein-Barr virus early gene BFRF1. Virology *347*, 109-116. |
| BRRF1 | Dickerson, S. J., Xing, Y., Robinson, A. R., Seaman, W. T., Gruffat, H., and Kenney, S. C. (2009). Methylation-dependent binding of the Epstein-Barr virus BZLF1 protein to viral promoters. PLoS Pathog *5*, e1000356. |

**Supporting Table S2: Oligonucleotides for quantitative real-time PCR amplification of ChIP**

| Region | Product size (bps) | forward primer sequence (5’ - 3’)  backward primer sequence (5’ - 3’) |
| --- | --- | --- |
| BBLF4 | 218 | GTGCGTGACCTGCTGACCAC  TCCGTCCCGATACTCCTGATG |
| BMRF1 | 194 | CACACCACCCCCCAAGGA  GCAGCAGCAGAAGCCAAC |
| TR | 203 | TACCGTTGTGACCGCCACC  AAAGCAGAGGAAGTGAACTGTTAGAGAC |

**Supporting Table S3: Oligonucleotides for PCR amplification of selected promoters**

| putative  promoter | genomic EBV coordinates B95.8 | Product size (bps) | forward primer sequence (5’-3’)  backward primer sequence (5’-3’) |
| --- | --- | --- | --- |
| BBLF4 | 114260-115010 | 751 | TCCTGTTCACCCCACGCCGG  CTCCAGCGTGCGGTAGCACA |
| BMRF1 | 79350-79898 | 549 | CTGCTGATTGAAGGCATCTT  GATCACAAGCAGCAGCAGAA |
| BALF5 | 156766-157266 | 501 | CCTCCCCAAGCAGTGCGGC  GAAATCAATTCGTGGACGG |
| BSLF1 | 86900-87900 | 1002 | CCCAACGGCGGGCTAAACA  ACGTCAGCCTGACCAACTT |
| BSLF2/BMLF1 | 84300-84900 | 601 | CTACGACGGGCTAGCTGGG  CGGAAGAGGAAGACAAGCA |
| BRLF1 | 106145-107249 | 1105 | GAGGTGTTGTGTCCTGTATG  CCCAACACCATGGGTGATA |
| BBLF2/3 | 119116-120085 | 970 | TCAACTGAGACCATCGCAATC  CCACCGAGCACAGGATAGG |
| BALF2 | 164767-165773 | 1007 | GCATGATGCCCAAGGTATC  TACTAAAAAGAAGGTGTTGGCAC |
| BZLF1 | 103159-103369 | 211 | CAGCAAAGATAGCAAAGGTGG  TTAGACACTTCTGAAAACTGCC |
| BHRF1 | 53610-53850 | 241 | TCGCCTTGCCTGCCTCAC  ACACACAGACGAAACCTTGACC |
| BHLF1 | 52760-53128 | 369 | GCACCCCCCGAGCGTCTGG  GGATAATGGAACCCTATGGATAC |

**Supporting Table S4: Oligonucleotides for quantitative real-time PCR amplification of selected transcripts**

| gene | Product size (bps) | forward primer sequence (5’ - 3’)  backward primer sequence (5’ - 3’) |
| --- | --- | --- |
| BZLF1 | 250 | GGTTTCCGTGTGCGTCGTG  AGCCTGCTCCTGAGAATGCTT |
| BRLF1 | 198 | TCGGGCTCGGGAGACTTTC  ATGGGGAATGATGGGGGTG |
| BMRF1 | 230 | TTGAGGTTTTACAGGTCTGGCATC  GGTGGCGGAGGTGAAGGAG |
| BALF5 | 170 | GGTGAGGCATCTGGGTGCTC  CCAAGAGTCATTTACACAAGGGCTAC |
| BSLF1 | 198 | GCGGGTCCTCTGGATTAGAT  CAGGGCGGTGGTCTTAGC |
| BMLF1 | 108 | CTGGGCAAGGTGACAAATGTAATC  GAAGCAGGCGAGGCAAGAAC |
| BBLF4 | 170 | GTCCTCCGTGGCTAAAAGCG  CAAGACCAAAAAGTCCATCTG |
| BBLF2 | 202 | GTCGGGAGTCTCGGTGGAATAG  AGCACAGGTGGTCTGCCAAAG |
| cytochrome c | 178 | CAATGCTCCGTTGTTGGCAG  CCTGGTGGGCGTGTGCTAC |

**Supporting Table S5: EMSA oligonucleotides for Kd-value determination**

| ZRE | genomic EBV coordinates B95.8 | Binding motive | forward primer sequence (5-3’)  backward primer sequence (5-3’) |
| --- | --- | --- | --- |
| BBLF4 ZRE A | 114487:114511 | **TGTGCGAG** | ATGACTCG**TGTGCGAG**CCGGTTTCG  CGAAACCGGCTCGCACACGAGTCAT |
| BBLF4 meZRE A |  |  | ATGACTCG**TGTGmeCGAG**CCGGTTTCG  CGAAACCGGCTmeCGCACACGAGTCAT |
| BBLF4 ZRE B | 114609:114633 | **TGAGCGTT** | CTCTCCTA**TGAGCGTT**ATGTGGACT  AGTCCACATAACGCTCATAGGAGAG |
| BBLF4 meZRE B |  |  | CTCTCCTA**TGAGmeCGTT**ATGTGGACT  AGTCCACATAAmeCGCTCATAGGAGAG |
| BMRF1 ZRE A | 79611:79635 | **ATGTGCGAG** | TTGGTGG**ATGTGCGAG**CCATAAAGC  GCTTTATGGCTCGCACATCCACCAA |
| BMRF1 meZRE A |  |  | TTGGTGG**ATGTGmeCGAG**CCATAAAGC  GCTTTATGGCTmeCGCACATCCACCAA |
| BRLF1 ZRE2 | 106363:106387 | **TGAGCGA** | CTAAGCTTA**TGAGCGA**TTTTATCAC  GTGATAAAATCGCTCATAAGCTTAG |
| BRLF1 meZRE2 |  |  | CTAAGCTTA**TGAGmeCGA**TTTTATCAC  GTGATAAAATmeCGCTCATAAGCTTAG |
| BRLF1 ZRE3 | 106423:106447 | **TTCGCGA** | CAGTCAAAA**TTCGCGA**TGCTATAAA  TTTATAGCATCGCGAATTTTGACTG |
| BRLF1 meZRE3 |  |  | CAGTCAAAA**TTmeCGmeCGA**TGCTATAAA  TTTATAGCATmeCGmeCGAATTTTGACTG |
| oriLyt ZRE5 | 53462:53486 | **TTGCACA** | TTGTCACCT**TTGCACA**TTTGGTCAG  CTGACCAAATGTGCAAAGGTGACAA |
| BMLF1 ZRE AP-1 | 84420:84444 | **TGACTCA** | GCGAAGCAC**TGACTCA**TGAAGGTGA  TCACCTTCATGAGTCAGTGCTTCGC |
| BHLF1 ZRE2 | 52844:52867 | **TTGCTCA** | TTTAAGGT**TTGCTCA**GGAGTGGGG  CCCCACTCCTGAGCAAACCTTAAA |

**Figure Legends**

**Supporting Figure S1: Analysis of GFP fusion proteins stably expressed in Raji cell lines.**

(A) The chimeric GFP:BZLF1 fusion protein consists of amino acids 149 to 245 of BZLF1 fused to the green fluorescence protein in the expression plasmid pEGFP-C1 (p3927.1). GFP:NLS served as a negative control consisting of the nuclear localization signal from SV40 T-antigen fused to GFP in pEGFP-C1 (p4247.1).

(B) Western blot immunodetection with an anti-GFP antibody showed similar expression levels of both fusion proteins. β-tubulin signals served as loading control. Single cell FACS analysis revealed a homogenous cell population for both cell lines.

(C) GFP:BZLF1 as well as GFP:NLS localize exclusively to the nucleus of transiently transfected HEK293 cells, which were used for preparation of GFP:BZLF1 protein for subsequent *in vitro* immunoprecipitation experiments with EBV DNA.

**Supporting Figure S2: GFP:BZLF1 binds sequence-specifically to selected EBV promoters *in vivo*.**

Native chromatin immunoprecipitation (ChIPs) experiments with Raji cells stably transfected with GFP:BZLF1 or GFP:NLS were performed with the GFP-nanotrap approach [1] followed by quantitative real-time PCR. *In vivo* GFP:BZLF1 binds tightly and selectively to the *BBLF4* and *BMRF1* promoters as compared to a reference locus in the terminal repeat (TR) region of EBV. The control derivative Raji cell line expressing the GFP:NLS fusion protein did not show any specific enrichment for *BBLF4* and *BMRF1* promoter DNAs.

**Supporting Figure S3: MeDIP (Methylated DNA Immunoprecipitation) analysis indicates a high degree of CpG methylation of genomic EBV DNA in Raji cells.**

DNA isolated from Raji cells was enriched for methylated DNA using a monoclonal antibody directed against 5’-methylcytidine as described [2] and either analyzed using qPCR (A) or hybridized to a custom-made EBV tiling microarray (B) as described previously [2].

(A) Analysis of CpG methylation of selected EBV promoter regions by qPCR.

MeDIP-enriched DNA of three independent experiments was quantified for precipitated DNA compared to input DNA in a Roche LightCycler 480 Real-Time PCR System at eight selected promoter regions. While the *C* promoter, the *LMP1* promoter, the *Q* promoter, the *W* promoters and the *BBLF4* promoter show a high degree of CpG methylation (exceeding 10% of input DNA), the *BZLF1* promoter and oriP show a lower degree of methylation (about 5% of input DNA). The *EBER* locus seems to be spared from methylation (below 1% of input DNA).

(B) Genome-wide microarray analysis of the methylation profile of EBV DNA in Raji cells.

MeDIP DNA and input DNA of three independent experiments were hybridized to a custom-made EBV-tiling array [2]. The array consists of four identical sets of 285 PCR fragments with an average length of 500 bp, covering the entire genome of B95.8 strain of EBV. Data was normalized such that the median of signal ratios was set to one. The methylation profile of Raji DNA indicates an overall high degree of CpG methylation in Raji cell DNA. An exception is the *EBER* locus, which is free of methylation in a region of >1kb (two spots on the microarray/two bars in the diagram), which is in line with the qPCR results in (A).

**Supporting Figure S4: Motif discovery of Zta binding to B95.8 DNA in ChIP-seq data.**

ChIP-seq data were selected via the SISSRs algorithms (default parameters) [3] and the output was used as the training set for MEME (Multiple EM for Motif Elicitation), which identifies gapless, local, multiple sequence motifs [4].

(A) A total of 39 motifs were identified shown as a consensus logo motif in the unselected SISSRs data training set.

(B) The identified motifs in (A) were selected at the level of the SISSRs training set data and grouped into ZRE motifs with (bottom panel) and without (top panel) CpG dinucleotides followed by MEME analysis.

**Supporting Figure S5: BZLF1 induces expression of genes essential for viral replication *in vivo*.**

A conditional expression plasmid [5] encoding a tetracycline-regulated *BZLF1* allele (p3862) was stably introduced into Raji cells. Total cellular RNAs were isolated before (-dox) and twelve hours after addition of doxycyclin (+dox). After reverse transcription relative levels of selected viral transcripts were assessed by quantitative real-time PCRs, which were normalized to the constitutive transcripts level of the housekeeping cytochrome c (*cyt*) gene.

**Supporting Figure S6: EMSA quantification of the fraction of selected Zta-bound unmethylated und methylated ZRE oligonucleotides and determination of K_d app_.**

In EMSAs, the fraction of Zta-bound oligonucleotides with selected, single ZREs was measured as a function of protein concentration and the Kd values of Zta and different unmethylated and CpG-methylated ZREs were determined as described [6]. EMSAs of typical experiments are shown as examples.

**References Supporting Files**

1. Rothbauer U, Zolghadr K, Muyldermans S, Schepers A, Cardoso MC et al. (2008) A versatile nanotrap for biochemical and functional studies with fluorescent fusion proteins. Mol Cell Proteomics 7: 282-289.

2. Kalla M, Schmeinck A, Bergbauer M, Pich D, Hammerschmidt W (2010) AP-1 homolog BZLF1 of Epstein-Barr virus has two essential functions dependent on the epigenetic state of the viral genome. Proc Natl Acad Sci U S A 107: 850-855.

3. Jothi R, Cuddapah S, Barski A, Cui K, Zhao K (2008) Genome-wide identification of in vivo protein-DNA binding sites from ChIP-Seq data. Nucleic Acids Res 36: 5221-5231.

4. Bailey TL, Boden M, Buske FA, Frith M, Grant CE et al. (2009) MEME SUITE: tools for motif discovery and searching. Nucleic Acids Res 37: W202-8.

5. Bornkamm GW, Berens C, Kuklik-Roos C, Bechet JM, Laux G et al. (2005) Stringent doxycycline-dependent control of gene activities using an episomal one-vector system. Nucleic Acids Res 33: e137.

6. Ryder SP, Recht MI, Williamson JR (2008) Quantitative analysis of protein-RNA interactions by gel mobility shift. Methods Mol Biol 488: 99-115.
