# Supplementary figures and images for: CpG-Methylation Regulates a Class of Epstein-Barr Virus Promoters
Source: PLoS Pathog. 2010 Sep 23;6(9):e1001114. doi: 10.1371/journal.ppat.1001114 (PMC2944802; doi:10.1371/journal.ppat.1001114)

**A**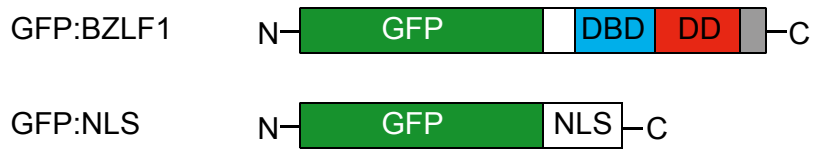**B**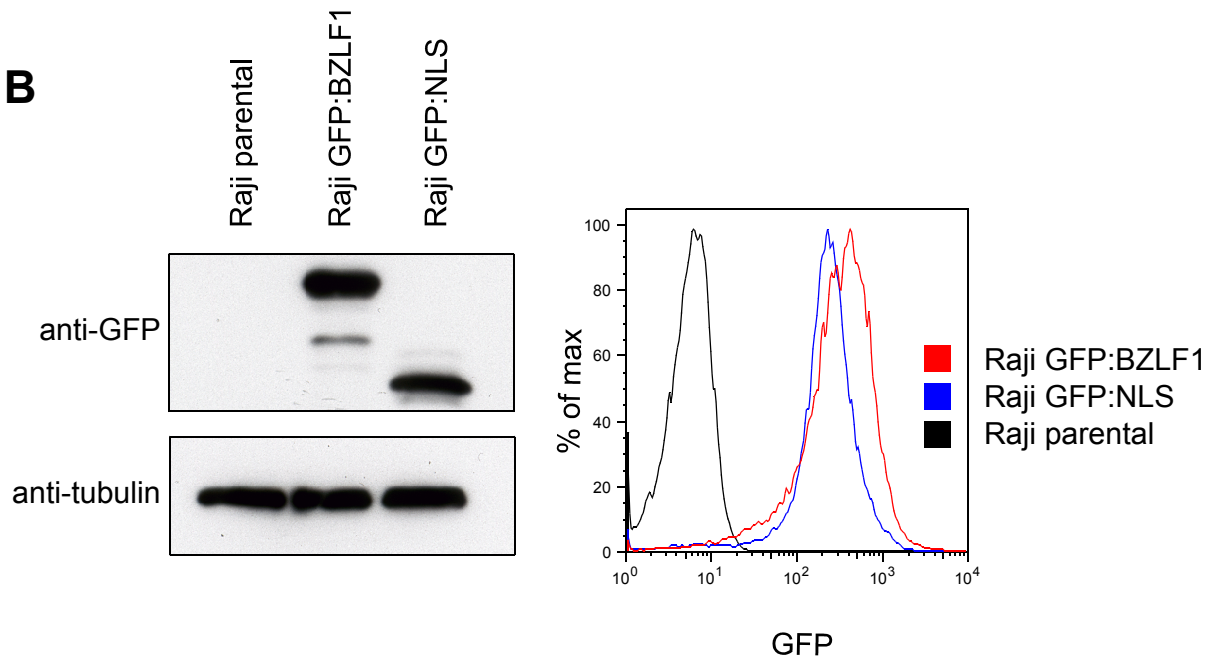**C**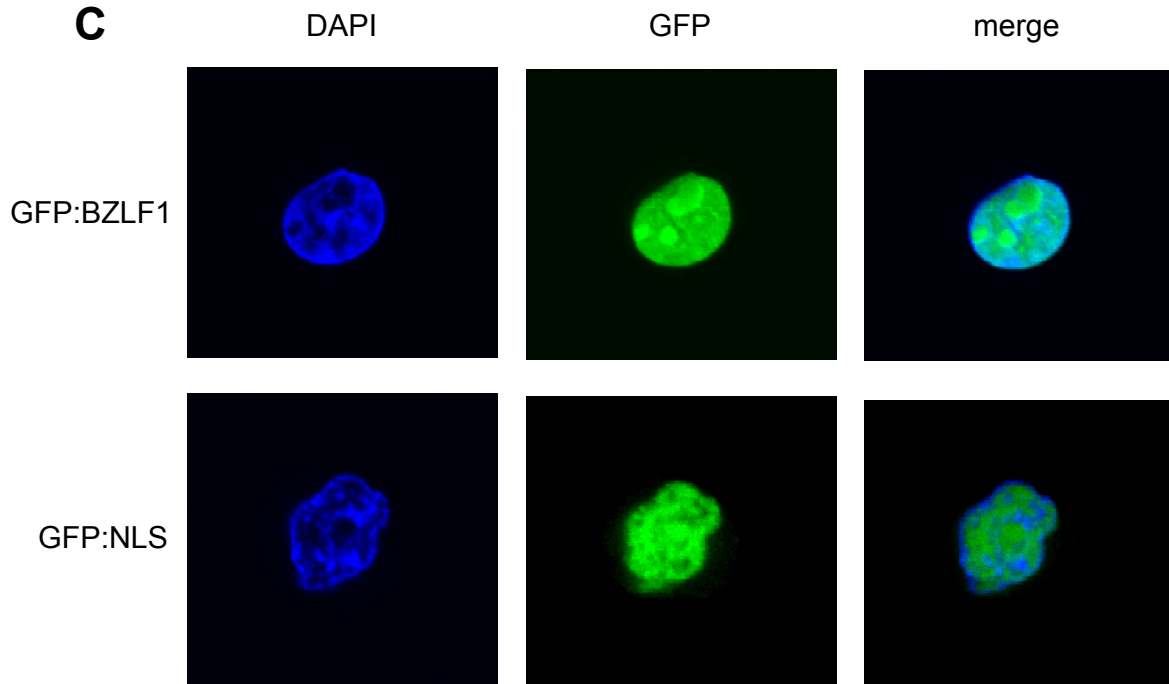

Supplement: Figure S1 — Analysis of GFP fusion proteins stably expressed in Raji cell lines. (6.84 MB PDF) [file ppat.1001114.s002.pdf]

native ChIP with Raji cell chromatin

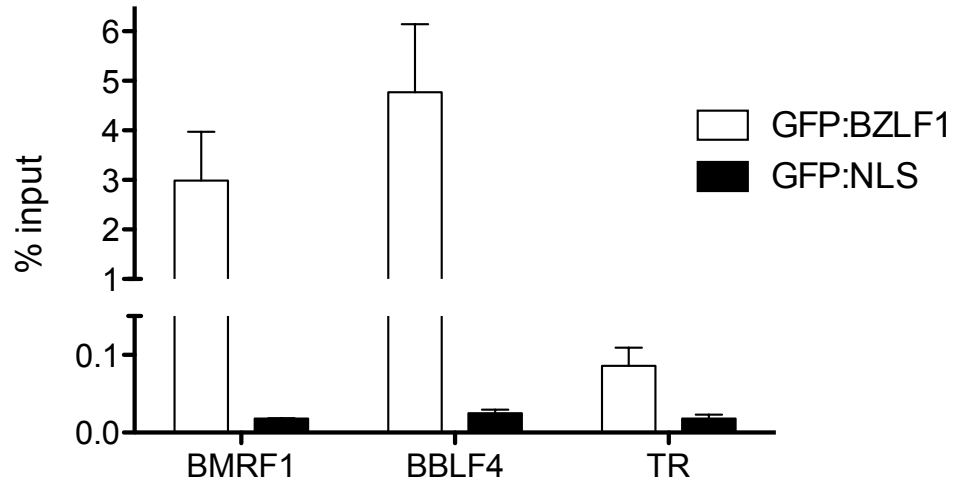

Bergbauer et al.,  
Supporting Fig. S2

Supplement: Figure S2 — GFP:BZLF1 binds sequence-specifically to selected EBV promoters in vivo. (0.02 MB PDF) [file ppat.1001114.s003.pdf]

**A**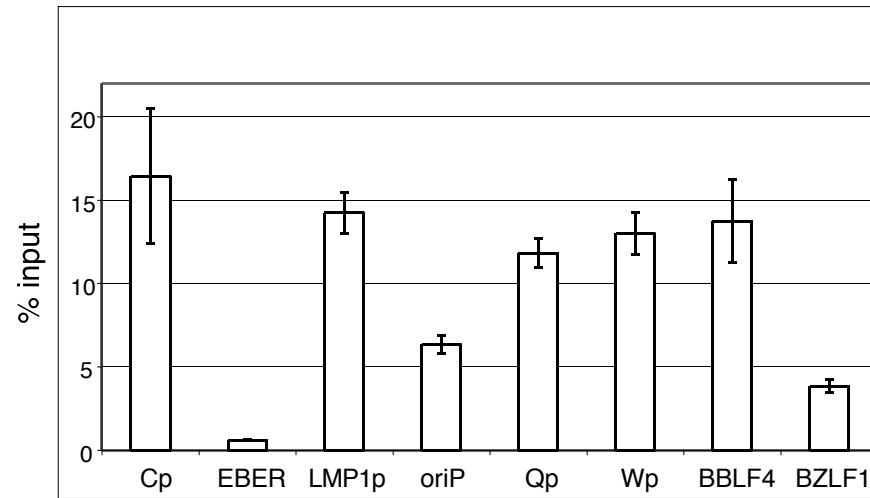**B**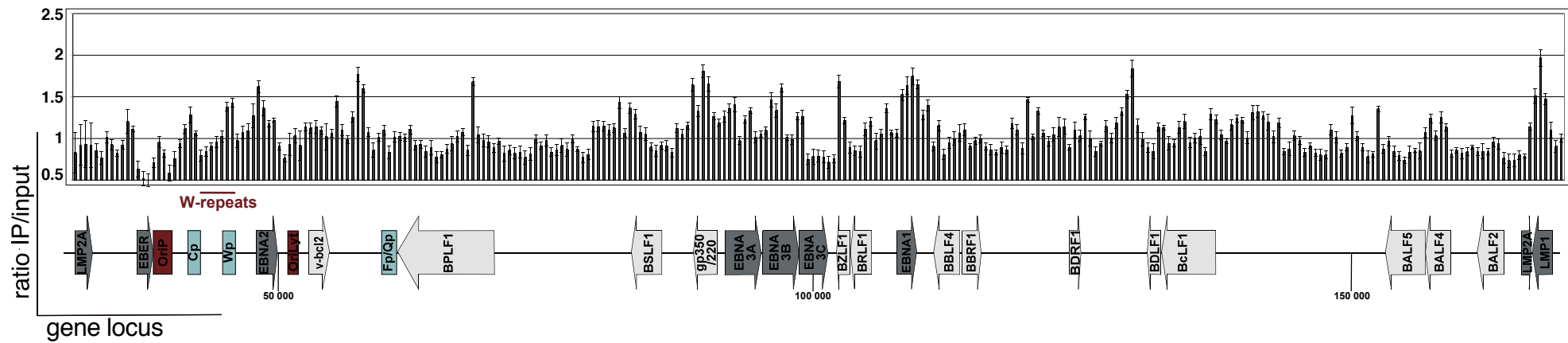

Bergbauer et al.  
Supporting Fig. S3

Supplement: Figure S3 — MeDIP (Methylated DNA Immunoprecipitation) analysis indicates a high degree of CpG methylation of genomic EBV DNA in Raji cells. (0.19 MB PDF) [file ppat.1001114.s004.pdf]

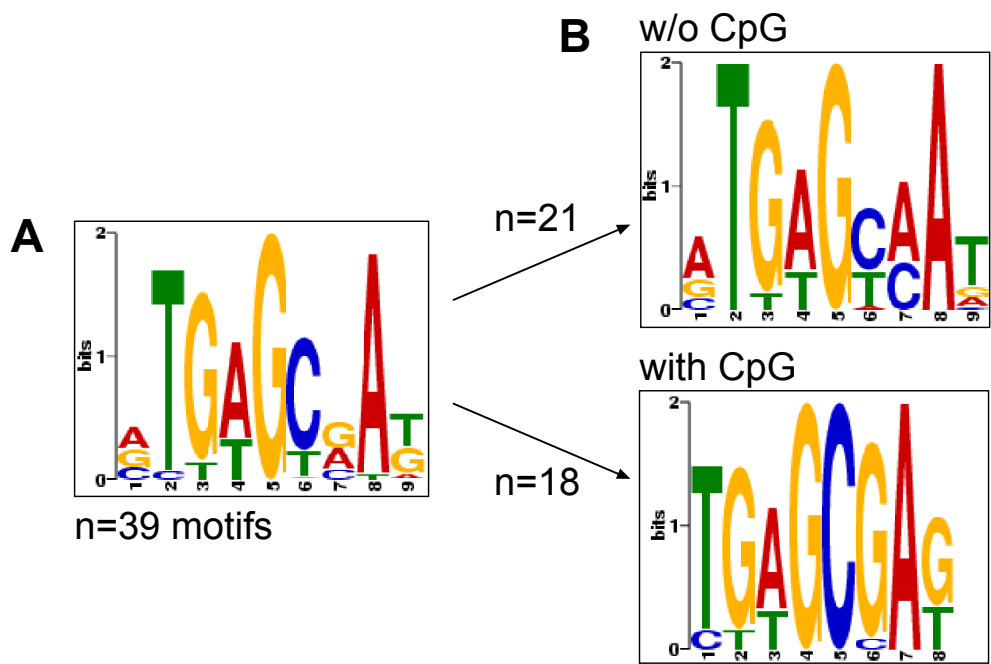

Bergbauer et al.  
Supporting Fig. S4

Supplement: Figure S4 — Motif discovery of Zta binding to B95.8 DNA in ChIP-seq data. ChIP-seq data were selected via the SISSRs algorithms (default parameters) [3] and the output was used as the training set for MEME (Multiple EM for Motif Elicitation), which identifies gapless, local, multiple sequence motifs [4]. (A) A total of 39 motifs were identified shown as a consensus logo motif in the unselected SISSRs data training set. (B) The identified motifs in (A) were selected at the level of the SISSRs training set data and grouped into ZRE motifs with (bottom panel) and without (top panel) CpG dinucleotides followed by MEME analysis. (1.18 MB PDF) [file ppat.1001114.s005.pdf]

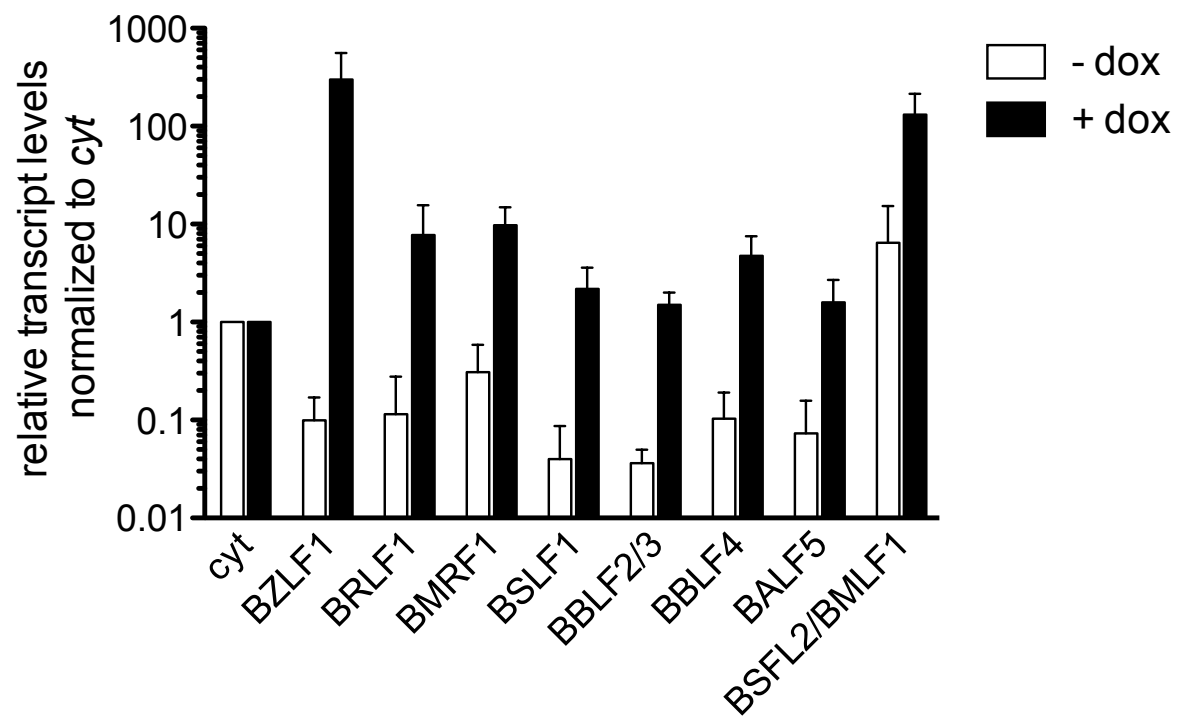

Bergbauer et al.  
Supporting Fig. S5

Supplement: Figure S5 — BZLF1 induces expression of genes essential for viral replication in vivo. A conditional expression plasmid [5] encoding a tetracycline-regulated BZLF1 allele (p3862) was stably introduced into Raji cells. Total cellular RNAs were isolated before (−dox) and twelve hours after addition of doxycyclin (+dox). After reverse transcription relative levels of selected viral transcripts were assessed by quantitative real-time PCRs, which were normalized to the constitutive transcripts level of the housekeeping cytochrome c (cyt) gene. (0.03 MB PDF) [file ppat.1001114.s006.pdf]

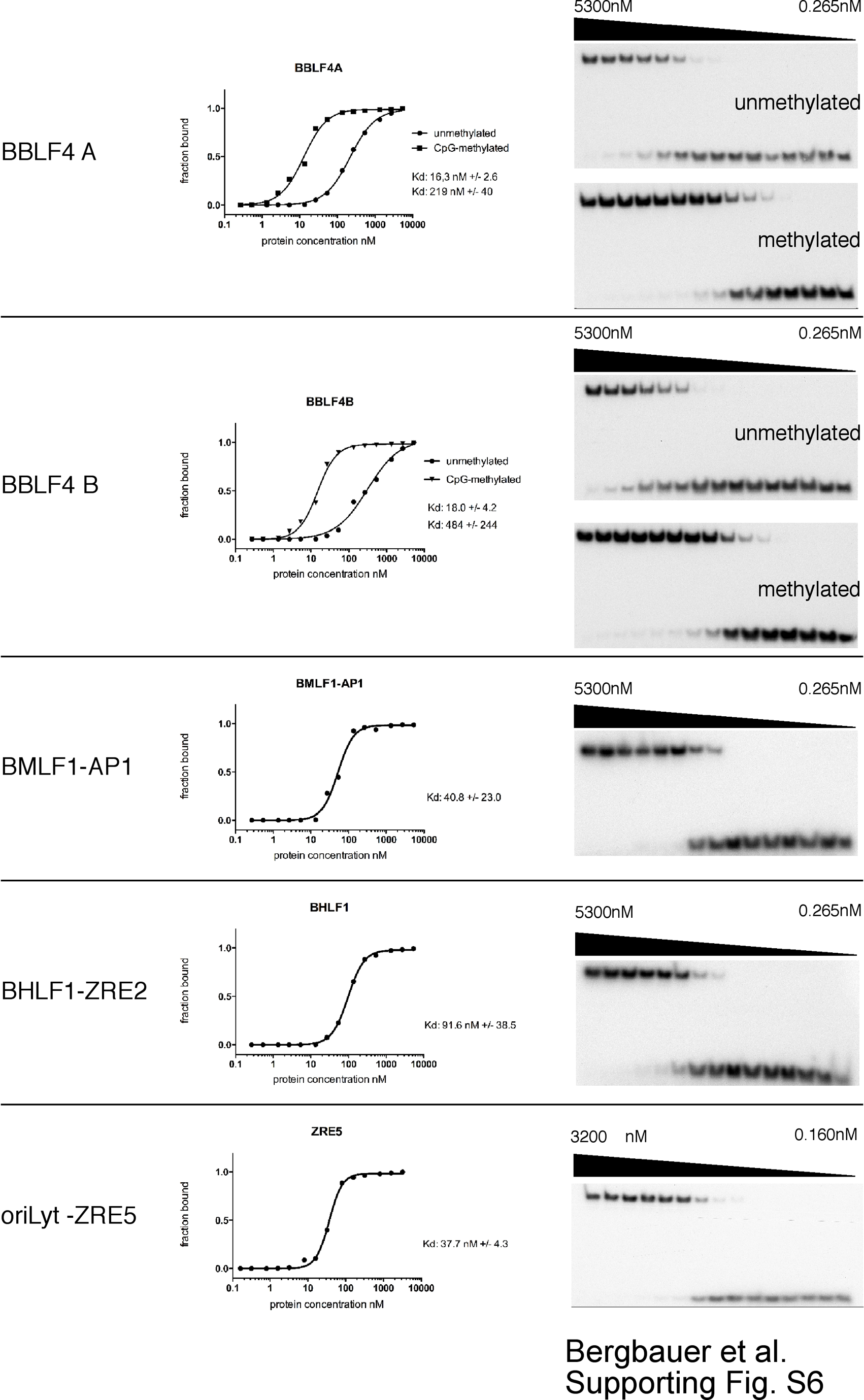

Supplement: Figure S6 — EMSA quantification of the fraction of selected Zta-bound unmethylated und methylated ZRE oligonucleotides and determination of Kd app. In EMSAs, the fraction of Zta-bound oligonucleotides with selected, single ZREs was measured as a function of protein concentration and the Kd values of Zta and different unmethylated and CpG-methylated ZREs were determined as described [6]. EMSAs of typical experiments are shown as examples. (1.55 MB TIF) [file ppat.1001114.s007.tif]
