# Supplementary material for: CpG-Methylation Regulates a Class of Epstein-Barr Virus Promoters
Source: PLoS Pathog. 2010 Sep 23;6(9):e1001114. doi: 10.1371/journal.ppat.1001114 (PMC2944802; doi:10.1371/journal.ppat.1001114)
Supplement: Supporting Data S1 — Training sets and MEME output files. (0.44 MB DOC) [file ppat.1001114.s008.doc]

**Supporting Data S1: Training sets and MEME output files.**

Four experiments were analyzed by next generation sequencing. Two samples were derived from *in vitro* DNA immunoprecipitation with unmethylated and CpG-methylated EBV DNA and two samples were obtained from *in vivo* chromatin immunoprecipitation (ChIP) with Raji and B95.8 chromatin. The results of next generation sequencing were mapped to the maxi-EBV genome p2089 [Delecluse HJ, Hilsendegen T, Pich D, Zeidler R, Hammerschmidt W (1998) Propagation and recovery of intact, infectious Epstein-Barr virus from prokaryotic to human cells. Proc Natl Acad Sci U S A 95: 8245-8250], a cloned derivative of the B95.8 EBV reference strain (Genbank entry V01555.2 GI:94734074) [Baer R, Bankier AT, Biggin MD, Deininger PL, Farrell PJ et al. (1984) DNA sequence and expression of the B95-8 Epstein-Barr virus genome. Nature 310: 207-211]. In B95.8, nucleotide coordinates # 152020 to #152022 were replaced with a 9837-bp long stretch of DNA encompassing the F-factor plasmid backbone as described [Delecluse HJ, Hilsendegen T, Pich D, Zeidler R, Hammerschmidt W (1998) Propagation and recovery of intact, infectious Epstein-Barr virus from prokaryotic to human cells. Proc Natl Acad Sci U S A 95: 8245-8250]. The reads mapped to the F-factor plasmid backbone of p2089 were deleted from the mapped data. Subsequently, the ChIP-seq data sets were analyzed by SISSRs (Site Identification from Short Sequence Reads) or QuEST (quantitative enrichment of sequence tags) as described in Materials and Methods. The four output files listed in the table below were used as so-called ‚training sets’ with the MEME (Multiple EM for Motif Elicitation) algorithm (MEME search tool: <http://meme.nbcr.net/meme4_4_0/intro.html>) to identify statistically overrepresented consensus motifs within the inferred binding sites. The characteristics of the four training sets, their sizes, the number consensus sites identified in the trainings sets, and the MEME output files are listed in the table below and on the following pages.

Table Supporting Data S1: Overview of the training sets and attached files

|  | Training set | Size of training set | number of identified sites in MEME |
| --- | --- | --- | --- |
| unmethylated p2089 DNA | SISSRs file name:*  meme w/o F-factor.txt | 126 | 101 |
| methylated p2089 DNA | SISSRs file name:*  meme-meth w/o F-factor.txt | 200 | 167 |
| Raji ChIP-seq | QuEST+ file name:*  46312.QuEST.params.old.peaks.meme | 46 | 46 |
| B95.8 ChIP-seq | SISSRs file name:*  GFP95.8.SISSRs.out.meme | 42 | 39 |

* nucleotide coordinates of the p2089 maxi-EBV genome DNA.

Training set: **meme w/o F-factor.txt**

>ebv:1051-1091,numTags=1456

TCTTCATGTCCACTGAGCAAGATCCTTCTGGCACGACTGT

>ebv:1111-1151,numTags=1720

TTGTTGCTAGCCTCCGCGCTAATCGCTGGTGGCAGTATTT

>ebv:1271-1311,numTags=1640

TTCCTTAGATTTGTTCTGCATGTTATTACTGATTGTCGCT

>ebv:1891-1931,numTags=674

CCGGGGCCCCTGATTGCTGAGAACCTGCTGCTAGTGGCGC

>ebv:10151-10191,numTags=314

TACATAGAAGCATACATGACAATTAAAGCCAGGTGATAAT

>ebv:101151-101191,numTags=3028

GCATGGCCGTGGGGTGTGACTCATCAGGTACAGCATGCCC

>ebv:101451-101491,numTags=922

TTTGATCTTTGACTGACACATAAACATGGTTTAAGGAATG

>ebv:103291-103331,numTags=3124

CTATGAGGTACATTAGCAATGCCTGTGGCTCATGCATAGT

>ebv:105351-105391,numTags=562

CCTGGCAAGAGTCTTGCAGGCTGTACAGCAGCACAAGCTG

>ebv:106211-106251,numTags=2486

TGAGCCATTGGCATGGGCGGGACAATCGCAATATAAAACC

>ebv:110091-110131,numTags=4469

TAGCGACATTTACCTGGTGAGCAATCAGACATGCGACGGC

>ebv:113951-113991,numTags=1252

TTCTGTGCGGCCACGGTTGTGGCTCCCGTGACCAGGCAGT

>ebv:114471-114511,numTags=379

AGCGATGGCCGAGCGATGACTCGTGTGCGAGCCGGTTTCG

>ebv:115651-115691,numTags=326

GGATCTGACTGAACTTTGGGAAAGCGAGCTGTTTAACACC

>ebv:116431-116471,numTags=449

TGTCCACCCTGGTCAAGTACCACGACCTGGAGGAGGGGGC

>ebv:119091-119131,numTags=465

GCAAATTATTTAAAAGGTGAATGCTCAACTGAGACCATCG

>ebv:120531-120571,numTags=293

GAGTAAGCATGGGGCTCTGATCGGGTCCGCCGGGTTCTCA

>ebv:121991-122031,numTags=3090

AGGTTCTCAGACTGCCCCCTGGTCATGGACTCCACGTACG

>ebv:122471-122511,numTags=3871

GAATTGACTCATATGCTCCGCCGTGACATCCACGCACGGA

>ebv:122791-122831,numTags=366

CCCTCAAGATGTAGCACTTGCTCAAAAGGCAGAGGGGCTT

>ebv:122931-122971,numTags=350

CGGTCGTGGAGGGAAGCAGTCCCATAATCAGTCAGGACCA

>ebv:123291-123331,numTags=631

GTCACAGAGTCATAGAGTTTGACCGTGGCATTATCTGCAT

>ebv:124331-124371,numTags=619

GCACATGGTGTATAAACCGCAGTGGGTAGCAGAATAGGGC

>ebv:124471-124511,numTags=640

CACCAGGGAGGTCCAGGTTTGGGAGTGGGCCAATATTTGC

>ebv:125111-125151,numTags=326

TATTGCCCAATGCTTCGAGCATACTGCCGCCACTATGGCC

>ebv:125151-125191,numTags=326

CCAGGCCCGTGTTTGTAGCTTCTGATGAATCATTACCCAT

>ebv:125971-126011,numTags=994

ACCTCTTTCCCATTAGCAAGAACCCCCTGCAGGACACGTG

>ebv:126111-126151,numTags=521

TATGGGTACGTAGTCGTTGTTCAGGGCCGTGCATGGCAGC

>ebv:126231-126271,numTags=578

ATAGCATGTCACACATTCCCCGTTATCACATCTGGTTAGC

>ebv:127491-127531,numTags=7979

GGGGGCTAAAAATATGAGCAATTCCCCTGACCAGGGCCCT

>ebv:129331-129371,numTags=1106

ATCTCGTCTGTGAACTCGGCTGACCAGGCCACCAGCTTTC

>ebv:129751-129791,numTags=436

GGCACCGGAATCGCCGCCGTGACTCACGACAGGGCGGACC

>ebv:130271-130311,numTags=1373

CTGACGACGTGCTGGTTGCGCTAGTCATGGCTCATTTTCT

>ebv:131031-131071,numTags=240

ATAACAGCGCCTGCCTTGTCTCTGGCGTGTGCCATCGCGT

>ebv:131351-131391,numTags=3075

CACCCCATCAAAGACATAATATGTCTCATAGTGGCAGTGA

>ebv:133231-133271,numTags=224

ATCCTCTGTTGCATCTTGGCAATCTCATCGGTATACAGAC

>ebv:133951-133991,numTags=1541

GGAGCTCCATTAACATTAGTCATACCTGCCAGGTATGTCC

>ebv:134531-134571,numTags=1393

CCCGCTGCTGAGGATTGCGCATAACAAAGGTGGCAACATC

>ebv:137131-137171,numTags=1841

TTGCTGGGTCTCCTTCCGTCCCCGTGAGCAATGGTGGGGA

>ebv:137891-137931,numTags=506

GCTACTTGTCTCATCACCAATTTGAGCGCTGCTTCGAGCA

>ebv:137991-138031,numTags=606

TGCTGGCTCATCTGAATCAGGTTACCAGGATCCCCCCCTG

>ebv:139491-139531,numTags=487

ACGAGCGCAAGACCCGCCTGGACATACTGGGCCGTAACGC

>ebv:139971-140011,numTags=413

ACGGTGGGCATGACACAGGACAACGGCATTCCCGTGCTTT

>ebv:141211-141251,numTags=285

GCGTCCTGGTAAAATTCTGGATCTTTGGAATCTGGCGGGG

>ebv:141371-141411,numTags=405

AGAGATTATGAATGTCACATTGATGAGCGGGATAATCATC

>ebv:142331-142371,numTags=474

ATAATGCACAAAGGTTGTAACAATCACCAGGGAATAGTCC

>ebv:142431-142471,numTags=445

TTCGTCTTGCCAAAGACCAGGCTCAACACATGCTCATCTC

>ebv:142951-142991,numTags=811

TGTCCAGGTGCAGCTTAACCTCGCTGAGGCTGGCAGCCCC

>ebv:143051-143091,numTags=813

CCCTCTCAAAATCAGAGATCACCTTGCTCAGACCAGCCCA

>ebv:143471-143511,numTags=1677

TCTTCAGGTAGCCAGCATCCAGTCCTGACTCATGTTTCCT

>ebv:144291-144331,numTags=257

CTCATCCTCAGACTCACAGTCAGATTTGTCATCGAGCCCA

>ebv:144691-144731,numTags=243

TCTTCCTCACCTGCCCCTGAGTCACTACCGGTTGGGGTAA

>ebv:145131-145171,numTags=1978

TATTGTAGAATTTAAGACGGCCATGAGCAACACGGCCAAC

>ebv:148171-148211,numTags=5434

GGACTCAATTTACCACGATTGCGATATTGCACAGAGTCAG

>ebv:150171-150211,numTags=684

TGTCACATTGCACGTGTAGTAACCTGCATGCGCAAGGGTC

>ebv:150491-150531,numTags=11164

CACAGCAGCACAATAGAAGCACTAAATGAGTCATTCCTAA

>ebv:150951-150991,numTags=4581

GCAATTGCGTGCCTAGAACATTGCTAATGTGCCCTGGGTT

>ebv:162091-162131,numTags=10900

AGTATAATGAGCAACAGGGCCAAACAGGCGGCGGGGCCTG

>ebv:162131-162171,numTags=10858

GGTAGTAGCGGGCAACGAGAGACTCTGTGCAATCAAACCC

>ebv:165071-165111,numTags=3882

ATCCATGACCAGGGCCGAGTCCTGCACGCAGTACATGCCG

>ebv:166811-166851,numTags=1898

TCACCAGAGGCATGTTGCTGAGCGAGCTCGTCGATCCCGG

>ebv:169611-169651,numTags=308

TACAGGCTGTGCGTCAGCGCGTGCAGGTGCTCCGAGCTCA

>ebv:171551-171591,numTags=306

AGGTTGCACACCACATCAAACACACAGTCTTCCTCCTGTT

>ebv:172131-172171,numTags=419

GGTTGCCAGGCACCACATCTATGACGTTGCTCTCTTCGTG

>ebv:172691-172731,numTags=539

TCATAGCACATACAGATGGGCAGGGAGATGTCCTGCAGGA

>ebv:174631-174671,numTags=1574

CAGTCCCACATTAAATGTTACACTTTACTCATCACGCAAC

>ebv:176691-176731,numTags=10313

CCCTACTCTCCACGGGATGACTCATCTCAACACATATACG

>ebv:177471-177511,numTags=2428

TATGCGCAAAGAATGTGTCAACAACAGGTGTTCCTGCCTC

>ebv:177991-178031,numTags=672

GGTTAGTCATAGTAGCTTAGCTGAACTGGGCCGTGGGGGT

>ebv:178131-178171,numTags=958

GTCTGTCATCAAAGGCGGGCCCTGGTCACCTCCTTTGTTT

>ebv:179031-179071,numTags=2193

AATACTTACTCATCAGTAGGAGTATACAAAGGGCTCCAAG

>ebv:179291-179331,numTags=234

TCAAGGTCGTGTTCCATCCTCAGGGCAGTGTGTCAGGAGC

>ebv:2491-2531,numTags=252

ACCCTTGACACATGGAAGGTGTTGAGCAGGGACACACGAA

>ebv:331-371,numTags=300

AGTTCACTTCCTCTGCTTTTCTCCTCAGTCTTTGCAATTT

>ebv:3951-3991,numTags=356

CTTGGATCCGGAAGACGTCTTGCTCACCCTGCATCTACAC

>ebv:4671-4711,numTags=2035

GCTGCTGGGAACCTGGTCATCCTTTGCCAGCGAGCAGTAC

>ebv:48971-49011,numTags=230

CAAACAGCCCCTCAGGCCAGGTTGGTCCAGCCACATGTCC

>ebv:49271-49311,numTags=5471

CAATCAATGCACCCTCTTACTCATCAAAGCACCCCAAATG

>ebv:5831-5871,numTags=844

AAACACTACAATAAACCCAATGTGCAAATGTGGTTTGTAT

>ebv:52831-52871,numTags=1787

TCTGTGTAATACTTTAAGGTTTGCTCAGGAGTGGGGGCTT

>ebv:53711-53751,numTags=9625

TTTTCTATGTGGGGGTGGAAATATGAGCAAGAATAAGGAC

>ebv:55271-55311,numTags=771

AGCACACACGTAATTTGCAAGCGGTGCTTCACGCTCTTCG

>ebv:56291-56331,numTags=476

ATGGTGCCACAAAAGTGTCCGGTGACCAGGTAAGCGTAGA

>ebv:56591-56631,numTags=265

ACGGCCTCTGAGACATGTATGGGGGTGTTCATCTCACGCA

>ebv:56751-56791,numTags=264

CAACCCTTTCCATTCTTCCCAGCTCTGAGCGAGATTTTCC

>ebv:57211-57251,numTags=1091

GTGGTTGATGGCACACAGGGGGTCGCAGAACAGGTGCTTG

>ebv:57271-57311,numTags=1058

CATCTGCAGCCAGGCCCTTAGCCTGGGGCATGGCACATCC

>ebv:58191-58231,numTags=1016

CGGGGGCATCTGAGTGATTGACCAGGGTGTCGAGCAGATC

>ebv:58511-58551,numTags=3101

ACTTTGTGAGCCATGACACATCTGGGTGGCAAGGTGAGGT

>ebv:59411-59451,numTags=249

CCAGAAGGCCTCGCGGGTCATGGATATGATTAGTGATATG

>ebv:6371-6411,numTags=284

GAGACCCTAGAATGTGTCGAAATGACCAAGCGTCCCCGCA

>ebv:70051-70091,numTags=397

GACTGAAGGAGCGATAGTTGAGACTGGCGCGGGGTGGGGT

>ebv:70111-70151,numTags=381

TTGGTGAGGGATGGGGATTACTGGAGGGGGAAGGCGAGCT

>ebv:71491-71531,numTags=1713

GGTGCGCTGGCTTTGCCCCCAGTCGCCGTTACTCATCTTC

>ebv:72411-72451,numTags=2299

AACAGGCGGGCGAATGTGTAATCCCGGAACCGGTAGGCGA

>ebv:76111-76151,numTags=2637

TCTTAAGAATTTATTACTCACCATCCATCATGCACCGCTA

>ebv:76531-76571,numTags=348

CGAGTCAGTTACACACACAGTAGCCGAATATCTGGAGGTC

>ebv:78731-78771,numTags=2383

GTTGACATCATGAGCAAGGGCCCAGGGGAGGGACCAGGTG

>ebv:79751-79791,numTags=2258

AATTTCTTTGAGCAAGAGAGTTCCGATTACACCATGCTGG

>ebv:8091-8131,numTags=862

TGCTTGGCGCTCAGGCGCAAGTGTGTGTAATTTGTCCTCC

>ebv:8551-8591,numTags=531

GGATTATGTGTTGCCCATAGCCATAAATTCGTGTGAGATG

>ebv:8651-8691,numTags=481

AATGTTTCATTCCTACACTAGTATTTATTGCCCAAGGGGT

>ebv:81911-81951,numTags=1809

CCATTCCAGCATCATTGCATGTGTCATGGCCACCCTCTGC

>ebv:84451-84491,numTags=3687

GGCCTGTGATGTGTAGTAGAGTACCAGAAACACCCTCACA

>ebv:84811-84851,numTags=366

GGCCGGCCAGCCCACGTGCAGTGAGTGATGTAAGAGGTTT

>ebv:86171-86211,numTags=322

CGCAGTTTCTCGTGTGCAAAGTGGGCAAAAGCCTCGGTCT

>ebv:87251-87291,numTags=597

GCTCAGTCATCTGGAATACCATGATCTCTCTGGCTGTAAG

>ebv:87411-87451,numTags=450

CTGGGCGCAAGAGATCTCGGCCGTTGTCTCATCGGTGACC

>ebv:88571-88611,numTags=247

TTGCAAAGGCTGTGCCACTGCTCTTCCTCGCCGCCACCTG

>ebv:88751-88791,numTags=508

CCATCTGGGCACTTCTGACGCTTGTCTTAGTCATTATAGC

>ebv:9151-9191,numTags=5281

AACACTATTGCTAATGCCCTCTTGAGGGTCCGCTTATCGG

>ebv:9431-9471,numTags=2022

GTTTGGTCCCCCCCCGTGTCACATGTGGAACAGGGCCCAG

>ebv:9571-9611,numTags=2085

AAATAGAACGCCCTGGAACTGCCCCACTGTGCAATGCAGC

>ebv:90991-91031,numTags=290

TCCGATTGCTCGCAAATGCACCAGAAATATTTTCACAACC

>ebv:91091-91131,numTags=255

CCAGGCCCAAAAGGCAGTCACTGTAACATTTGGCGAGTTT

>ebv:91151-91191,numTags=247

TGGCACTGAATAGGTAGCATTGTCACCCACATATGTGATG

>ebv:91311-91351,numTags=287

AGGATACTGTTATTGCCAAGAAATCGTGACACTGGACGTG

>ebv:92191-92231,numTags=844

AGGTACAAGGGGGGTGCGGTGTTGGTGAGTCACACTTTTG

>ebv:92831-92871,numTags=245

GCCGGATGGCCTATGGGTTACCAGGCATACAGCAGCTGGA

>ebv:95091-95131,numTags=556

TGGTGTCAGCTGTTGTTCACATGTGTCAGGATGACGAGTT

>ebv:95271-95311,numTags=292

TTCATATTCCATGAGAGAGACCTCGCATATTTGCAGAAGG

>ebv:95411-95451,numTags=268

CGAGGACCCACCAGATTATGGAGATCAAGGTAATGTGACA

>ebv:95851-95891,numTags=2205

TATGGATGTGCATGACCGTGCGTCACAGATGTCAGGCCAT

>ebv:98231-98271,numTags=2021

ATCGAACGATGAGTGATTTCGCCCATGTAACAAGAACTGG

>ebv:98291-98331,numTags=1829

ACTGCGGGGAGGAGGGGGGCAGTGATAAGTCATGACAATT

>ebv:98791-98831,numTags=425

CCATTAATTTTAGCAATCGCACCTGCAAGCGCTATCAAAC

Training set: **meme-meth w/o F-factor.txt**

>ebv:1111-1151,numTags=6405

TTGTTGCTAGCCTCCGCGCTAATCGCTGGTGGCAGTATTT

>ebv:1531-1571,numTags=4442

CACCGTTCTGACACATGACTTACATGGGTTTGGCTTTTGT

>ebv:1711-1751,numTags=5368

TCACGTGTAACTTGACGTGCAAGGATGGAAGAGAGGGGCA

>ebv:1871-1911,numTags=3954

GCTCCGCGCTCGGCCTAGACCCGGGGCCCCTGATTGCTGA

>ebv:1991-2031,numTags=2061

TCGTTGGCATTCTACTAGGAAACGGCGAGCAGGGTGAACA

>ebv:11091-11131,numTags=2307

GACGCAGGGCTCGCAAAGTATAGTGGCCCCGTGGGACCTT

>ebv:11211-11251,numTags=1887

CTTGCTTACGCAAGCTCAGTTAATTCGCCCACGACTTGAA

>ebv:100471-100511,numTags=9299

AACGCCTCGCCCATCATGGGCCCCATCAGTCTGCGCCCTC

>ebv:100571-100611,numTags=9420

CCCACACAGCCGATATCGCACGAAGAACAACCCCGGTATG

>ebv:101151-101191,numTags=4538

GCATGGCCGTGGGGTGTGACTCATCAGGTACAGCATGCCC

>ebv:101451-101491,numTags=1515

TTTGATCTTTGACTGACACATAAACATGGTTTAAGGAATG

>ebv:102531-102571,numTags=1893

TGCGAGCAAGGGAATGCGTTACTACAAGTGGTGCCTAGTC

>ebv:102651-102691,numTags=2122

TACCGATTCTGGCTGTTGTGGTTTCCGTGTGCGTCGTGCC

>ebv:103271-103311,numTags=4629

CGTGCTAAATTTAGGTGTGTCTATGAGGTACATTAGCAAT

>ebv:105471-105511,numTags=5146

TGAACAGCGTGACATCGCCGAGGTTCTTGACCACCTCAAG

>ebv:106351-106391,numTags=39554

TGGGGCATTAACTAAGCTTATGAGCGATTTTATCACAGGA

>ebv:106891-106931,numTags=3710

AATCCCTGACCTCGATGACTCCGATGCTGTTTTCGCACGC

>ebv:109551-109591,numTags=1517

CCATTGAGTCGTCTCCCCTTTGGAATGGCCCCTGGACCCG

>ebv:110071-110111,numTags=14021

CTTCTCGCGTTGGAAAACATTAGCGACATTTACCTGGTGA

>ebv:110811-110851,numTags=7921

GTGCAAAAGGGCAAGCCCGGCTCGCACGCAGACATTGGCT

>ebv:110891-110931,numTags=8163

GCGGTTAAAAGCGTGCGTGTTTATGCTGTGGGGTGCGAAG

>ebv:112711-112751,numTags=1519

GTAGATGCCTCCGTAGATGAGTCGGGCCAGGAAGCTGTAG

>ebv:112831-112871,numTags=1200

GGTATAGCCACAGATGGACTTTTTGGTCTTGCAGTTGACC

>ebv:113951-113991,numTags=4107

TTCTGTGCGGCCACGGTTGTGGCTCCCGTGACCAGGCAGT

>ebv:114471-114511,numTags=38235

AGCGATGGCCGAGCGATGACTCGTGTGCGAGCCGGTTTCG

>ebv:114591-114631,numTags=27817

TACACAGCCGCTCCGTGCTCTCCTATGAGCGTTATGTGGA

>ebv:115331-115371,numTags=1066

GATCAGATAAACGAGCAATTTGACCAGATTAATGGCCTAG

>ebv:116411-116451,numTags=1062

CAATGGGGCCAGGACGCTGCTGTCCACCCTGGTCAAGTAC

>ebv:117871-117911,numTags=895

TCCCTCGCCGCCTTCCCGCGTGCAAACGTGGCGAGGGTTA

>ebv:118791-118831,numTags=6112

GCTCCCGCTCCCATCGCAAAAAGTTGAGTTCGGTAGTCGA

>ebv:118931-118971,numTags=5495

ATGCCCACGACCCAAATCGCCCACCACCGCCCTGCGGTCT

>ebv:119091-119131,numTags=4035

GCAAATTATTTAAAAGGTGAATGCTCAACTGAGACCATCG

>ebv:119771-119811,numTags=12892

CCTTGCCCTGGAGACGCTGGTCTTCTCGCTCAGCGTGTTC

>ebv:120171-120211,numTags=1671

CTAGAGTCAGAGCCGAGGCCTAGGCCCTCGCGCACGCCAT

>ebv:120911-120951,numTags=1275

TCCCCCGTCCACGTCGCCTATGGAGTTGACTCGTCGTCGG

>ebv:121991-122031,numTags=4347

AGGTTCTCAGACTGCCCCCTGGTCATGGACTCCACGTACG

>ebv:122471-122511,numTags=10006

GAATTGACTCATATGCTCCGCCGTGACATCCACGCACGGA

>ebv:122791-122831,numTags=3171

CCCTCAAGATGTAGCACTTGCTCAAAAGGCAGAGGGGCTT

>ebv:123291-123331,numTags=1069

GTCACAGAGTCATAGAGTTTGACCGTGGCATTATCTGCAT

>ebv:123931-123971,numTags=2335

CGCTTAGAACTACTCATCTTCATAAGTCACCATGTCCGCA

>ebv:124111-124151,numTags=2954

TTTATACTGAGCGTTTAGGTTTTGTTTATGTAGCAAGCAC

>ebv:124471-124511,numTags=1072

CACCAGGGAGGTCCAGGTTTGGGAGTGGGCCAATATTTGC

>ebv:124971-125011,numTags=1040

AGAACCTAAGAAACGCCCTTCAGCAGGACAGCACCACGCA

>ebv:125091-125131,numTags=1305

CAATTCACGCCAGTAATTTATATTGCCCAATGCTTCGAGC

>ebv:125671-125711,numTags=1192

GAATCTCTCCAACGTGCAAATAGGCTACGTGGCTCACCAG

>ebv:125851-125891,numTags=1616

TGCCACCTGCTTCAATAAGAATGTAAGACCTGACGTTTCA

>ebv:125971-126011,numTags=2218

ACCTCTTTCCCATTAGCAAGAACCCCCTGCAGGACACGTG

>ebv:126091-126131,numTags=1320

AACAAAGCCCGATGCCCAGTTATGGGTACGTAGTCGTTGT

>ebv:126231-126271,numTags=1195

ATAGCATGTCACACATTCCCCGTTATCACATCTGGTTAGC

>ebv:126771-126811,numTags=20597

GACTTTCATCTGGGGCGTAGAGGCATCGCTCAGCACCCAG

>ebv:127491-127531,numTags=12707

GGGGGCTAAAAATATGAGCAATTCCCCTGACCAGGGCCCT

>ebv:128451-128491,numTags=1230

CTTTTGTGAGTAAATAGAGATGATGACATGGATGTAGAGA

>ebv:128591-128631,numTags=1365

GCGCCCCCTCCTGATCAAAGAGGGCCTCGCTGACCCCGGA

>ebv:129431-129471,numTags=5093

AGCATCGGCAAGATTTTGACATGCAGGACAGCATGGTCTC

>ebv:129571-129611,numTags=6629

CTGATGGGTGACACCTCCTCGCTGAGCCAGGGTAGCCTGA

>ebv:129731-129771,numTags=5689

CCAACAACACATCCGCATCAGGCACCGGAATCGCCGCCGT

>ebv:130291-130331,numTags=3231

CTAGTCATGGCTCATTTTCTCGCAACAACACAGAAGCACA

>ebv:131151-131191,numTags=5826

TCCTCGAGGTATGCAGGGAATGAGCGGTCCGTGAGCCGGT

>ebv:131391-131431,numTags=11269

TGATGCATCACCACCACAGCACTCGCCAGGACCCTCTGCA

>ebv:133951-133991,numTags=3705

GGAGCTCCATTAACATTAGTCATACCTGCCAGGTATGTCC

>ebv:134451-134491,numTags=3123

CGCGTATTTGCCCATGGGCGAGCGGTGCCACTCCCGGTAC

>ebv:134871-134911,numTags=1189

GAGCGTCCCATTCTCCAAATTGTCGAGGATGTCCTCGTCC

>ebv:136711-136751,numTags=10820

ACCATCTCAGAGAGACGGCTTCGCACGTACTGAGAAAACC

>ebv:137131-137171,numTags=8204

TTGCTGGGTCTCCTTCCGTCCCCGTGAGCAATGGTGGGGA

>ebv:137911-137951,numTags=1883

TTTGAGCGCTGCTTCGAGCAGTTCTGCCGCGTGGTCCCGC

>ebv:137971-138011,numTags=1931

TAAGCGAAACTCTGCAAAGATGCTGGCTCATCTGAATCAG

>ebv:138631-138671,numTags=1363

GCGTCGAGACGCCCCTCCACCCCCGCTCGATTCCAGCTTC

>ebv:138751-138791,numTags=1373

GGTGTGCTCAGAGCCCCTGGTGAAGATGCGAGTTTGCAAT

>ebv:139451-139491,numTags=5241

GGTCACAGACATTCGGCGAACGTGACAAAGCTGGTGGTAA

>ebv:139951-139991,numTags=1502

ATATCCTAGAGGGACAGCTGACGGTGGGCATGACACAGGA

>ebv:140391-140431,numTags=1267

GCGCAGGTCAGCAACGTGCTCATCGCCAACCGCTCCCACA

>ebv:141371-141411,numTags=4771

AGAGATTATGAATGTCACATTGATGAGCGGGATAATCATC

>ebv:142331-142371,numTags=2531

ATAATGCACAAAGGTTGTAACAATCACCAGGGAATAGTCC

>ebv:142951-142991,numTags=5095

TGTCCAGGTGCAGCTTAACCTCGCTGAGGCTGGCAGCCCC

>ebv:143471-143511,numTags=4342

TCTTCAGGTAGCCAGCATCCAGTCCTGACTCATGTTTCCT

>ebv:144291-144331,numTags=1070

CTCATCCTCAGACTCACAGTCAGATTTGTCATCGAGCCCA

>ebv:144591-144631,numTags=976

CACGGCAGCCTGGGTCCGGCACGGCACCCTCTCCCCAGAC

>ebv:145211-145251,numTags=25745

CCGCCCAGTTGTGTGACTGTGCCAATTTTCTTCGCACGTC

>ebv:146211-146251,numTags=1337

CGGAGAGCCTAGAGAAAATCATCGCCGACCTCAAGGCCAA

>ebv:147151-147191,numTags=10643

GTCATTGGCAAAAAGGTAAATAAACTCATCGCACGGGGGT

>ebv:147591-147631,numTags=2065

GAGACCGTGAGCGGTTACCGGGGCGCAGGGCCTCTGCCGG

>ebv:148171-148211,numTags=13968

GGACTCAATTTACCACGATTGCGATATTGCACAGAGTCAG

>ebv:148971-149011,numTags=2348

ACTACGCCGGGCCCGCGGGCGGGGACCCGGGTGCCTTCTT

>ebv:150171-150211,numTags=8893

TGTCACATTGCACGTGTAGTAACCTGCATGCGCAAGGGTC

>ebv:150491-150531,numTags=27336

CACAGCAGCACAATAGAAGCACTAAATGAGTCATTCCTAA

>ebv:150951-150991,numTags=12433

GCAATTGCGTGCCTAGAACATTGCTAATGTGCCCTGGGTT

>ebv:162131-162171,numTags=27260

GGTAGTAGCGGGCAACGAGAGACTCTGTGCAATCAAACCC

>ebv:163051-163091,numTags=1317

CGCCCTTGCGTGTCCATTGTTGCAAGGAGCGATTTGGAGA

>ebv:163311-163351,numTags=2134

TCTTTGCCAGCCTTCATACTAGATTCAGCGATCCCCCGGT

>ebv:164451-164491,numTags=7214

TGCAGCGTCACCGTCTCGGCGATGGAGAGGCAGGGAAAGA

>ebv:165011-165051,numTags=24255

GATGTGAGCGATCTTGGCAATCTCTGCCACCTCCACGTGG

>ebv:165911-165951,numTags=1090

CTTGTCTTGCAGCCACGTGGCCACGTGACACACACTGTTG

>ebv:166431-166471,numTags=1037

GGGGGCTGAGGCCCCCGCACATCCACCACCCCTGCGGCGC

>ebv:166811-166851,numTags=21944

TCACCAGAGGCATGTTGCTGAGCGAGCTCGTCGATCCCGG

>ebv:168311-168351,numTags=3068

TAAGTGCCCTTGTCCAGGAAGGCTCGGCGTTCGCCTTGCG

>ebv:168391-168431,numTags=3082

AGTTCTCACGTGGAAGGAGTCTGCCCGCTCATGGAAGGTT

>ebv:169131-169171,numTags=1168

CGCTTAGCACCCTACGCCGAGTCATCTCTCATTTGGAGGT

>ebv:169511-169551,numTags=2801

GGGCAGCGTACCTGCGGCGTCACAGCAGCGAGCCAGGGCC

>ebv:169711-169751,numTags=843

CGGCGCGCCTGTCCTCCCAGGGACCCGAGACGAAGGCCCG

>ebv:169831-169871,numTags=963

TCAGGGTCTCGCTCTGTTGCGCCAGGCAGGACTGCAGCTT

>ebv:170651-170691,numTags=1956

CAGAACCATCAGCTCGCCCACACAGCGCCAGCAGGGCACA

>ebv:171431-171471,numTags=8207

CTGGAGGCAATGGTCGCCAGGGTTTCTAGGACGCTGTCCG

>ebv:171491-171531,numTags=8234

AATAAGTACTCCGCGTCGTCCCTAGTCAGCGAGGCGCATG

>ebv:172111-172151,numTags=1129

CTGCTTGGCGTAAGTGACCAGGTTGCCAGGCACCACATCT

>ebv:172731-172771,numTags=3504

TGGTCAGCAGTGAGCGGTAAAACAGCTGGGTGAAGATGGG

>ebv:173351-173391,numTags=2278

GCAAGAAGGTTGGGCGAGAAGGAGGCCGCATAGACCAGGT

>ebv:173491-173531,numTags=1486

CAGGGTCCCCAGGCCGTGGGTCATGTAGAAACTGTTAAAG

>ebv:173691-173731,numTags=2275

GCCACGAGTCGTAGTTGAGGCTGGCCGGGGTCTCGTGCGA

>ebv:174771-174811,numTags=17273

CCAGCCTCGCAAACCAGAGTCTGCGATAGAGGGCCAGGTA

>ebv:175331-175371,numTags=27235

AGAGCAATGGCCAGGTTCATCGCTCAGCTCCTCCTGTTGG

>ebv:175671-175711,numTags=1326

AGGTCACCAAGCAGGAACACCTGAGCGTGGTGAAGCCTCT

>ebv:176691-176731,numTags=19901

CCCTACTCTCCACGGGATGACTCATCTCAACACATATACG

>ebv:177391-177431,numTags=39020

GTGGAAAGATGAGCGAGGACAGGTGTGGAGGTTTTGGGCT

>ebv:177991-178031,numTags=1277

GGTTAGTCATAGTAGCTTAGCTGAACTGGGCCGTGGGGGT

>ebv:178131-178171,numTags=1600

GTCTGTCATCAAAGGCGGGCCCTGGTCACCTCCTTTGTTT

>ebv:179031-179071,numTags=5493

AATACTTACTCATCAGTAGGAGTATACAAAGGGCTCCAAG

>ebv:179851-179891,numTags=3989

GGTGGCGGATTCAGGCGAAAAGGGTGTGGGCTGTGCGAGT

>ebv:2511-2551,numTags=5655

GTTGAGCAGGGACACACGAACCGCTCATAGTTTGGAGCAC

>ebv:3971-4011,numTags=1184

TGCTCACCCTGCATCTACACCTGGATCCGCGCCGGGCAGA

>ebv:4051-4091,numTags=1003

TACGCGCGTGGCCTGGGCGTGAAGCTGACCTTTGGCTCGG

>ebv:4091-4131,numTags=1141

CCTCCTGCCCCGAGACCGGCTCGTCCGCCTCCAACTTCAT

>ebv:4371-4411,numTags=6755

GTCAGGGCATGACATCAGCGACGGGGGCCTGGTGACCTGC

>ebv:4671-4711,numTags=5632

GCTGCTGGGAACCTGGTCATCCTTTGCCAGCGAGCAGTAC

>ebv:48111-48151,numTags=1508

GATTAACGTGCAAGACGCTAAACTTAACCAAGGTCAGCCA

>ebv:49271-49311,numTags=7443

CAATCAATGCACCCTCTTACTCATCAAAGCACCCCAAATG

>ebv:5831-5871,numTags=1402

AAACACTACAATAAACCCAATGTGCAAATGTGGTTTGTAT

>ebv:52831-52871,numTags=2953

TCTGTGTAATACTTTAAGGTTTGCTCAGGAGTGGGGGCTT

>ebv:53611-53651,numTags=12082

GCCTTGCCTGCCTCACCATGACACACTAAGCCCCTGCTAA

>ebv:53711-53751,numTags=13997

TTTTCTATGTGGGGGTGGAAATATGAGCAAGAATAAGGAC

>ebv:55271-55311,numTags=1454

AGCACACACGTAATTTGCAAGCGGTGCTTCACGCTCTTCG

>ebv:56751-56791,numTags=16911

CAACCCTTTCCATTCTTCCCAGCTCTGAGCGAGATTTTCC

>ebv:57211-57251,numTags=3433

GTGGTTGATGGCACACAGGGGGTCGCAGAACAGGTGCTTG

>ebv:57271-57311,numTags=3278

CATCTGCAGCCAGGCCCTTAGCCTGGGGCATGGCACATCC

>ebv:57351-57391,numTags=2630

CTAGGATTTTGATGTTGTTGCCGAACGAGTCAAGAATCAG

>ebv:58171-58211,numTags=1504

ATAGTCCTCAAAGAAGGCCACGGGGGCATCTGAGTGATTG

>ebv:58511-58551,numTags=5371

ACTTTGTGAGCCATGACACATCTGGGTGGCAAGGTGAGGT

>ebv:59431-59471,numTags=909

TGGATATGATTAGTGATATGTCTCAGCAACTGTCTCGGTC

>ebv:6931-6971,numTags=1058

GAGTTACGGTTCGCTACATCAAACAGGACAGCCGTTGCCC

>ebv:6971-7011,numTags=1094

TAGTGGTTTCGGACACACCGCCAACGCTCAGTGCGGTGCT

>ebv:61451-61491,numTags=1374

GTAGAATTGCCACCTGGTGAGCGGTGTGCGGGCTGCCGTC

>ebv:62431-62471,numTags=2855

GGGATAGCGTGCGCTACCGGATGGCGGGTAATACATGCTA

>ebv:64091-64131,numTags=1925

CGTGCAAAGATGGACATCTCTGGCTCGAAAGACTCGGAGT

>ebv:64131-64171,numTags=1879

AGCCGTCCAGGTCCTGCAGAAAATTCAGCGAGATGGTCTC

>ebv:64591-64631,numTags=9698

GAAGCACCGGCGTGCGAGGAGCAGCATGCAGGCTCGGGCG

>ebv:65071-65111,numTags=1517

GATGAGACTGGCGTGCGCTAAAAGTGTCATGGCCACAATG

>ebv:66611-66651,numTags=12604

ACGTACTCGCTCACGATGGCCGTCAGGGCAGCCTCGGCTG

>ebv:67491-67531,numTags=4679

GTTTCTTGAGCTTCCCTGGGATGAGCGTTTGGGAGAGCTG

>ebv:67971-68011,numTags=11728

ACCCTGCATTTGCGATGTACTTTTCTATGACGGGGATGGT

>ebv:68271-68311,numTags=3811

CCAGGGGCCAGGGGATACCGCTCATATCACTAAGGGCGGT

>ebv:69011-69051,numTags=2392

TTGTTTTCTGATAGCATGTTTGCGAGGTTTTGGATGTTAA

>ebv:711-751,numTags=6439

ACTGGCTTTCGTCCTCTGGCTCTCTTCGCCAGGGGGCCTA

>ebv:70051-70091,numTags=2031

GACTGAAGGAGCGATAGTTGAGACTGGCGCGGGGTGGGGT

>ebv:70631-70671,numTags=915

CGCCGTTCAAACGGTAGCATGACGGGAGGGCTGCTATCAG

>ebv:71071-71111,numTags=1140

AACAACTGCCGAGTTTGCGATCTGGGCAGGGAATAGGACG

>ebv:71131-71171,numTags=1175

GCGGTATATGTGCACGCGCCCACCGCCCCTCAGGACCACG

>ebv:71491-71531,numTags=4919

GGTGCGCTGGCTTTGCCCCCAGTCGCCGTTACTCATCTTC

>ebv:72071-72111,numTags=2273

CACGAGGTGGTGGGTCTGGACCCGGGCCGTGCGAGCAAAG

>ebv:72411-72451,numTags=5636

AACAGGCGGGCGAATGTGTAATCCCGGAACCGGTAGGCGA

>ebv:74971-75011,numTags=2029

GGAGGACCGCCGCCGGGTAAGCGTGGCCGCTCATGAGGGT

>ebv:75351-75391,numTags=1241

GTTCGTGGGCTGGTTGAGGAACACGCGCAGGCCTCCTCGC

>ebv:76111-76151,numTags=4573

TCTTAAGAATTTATTACTCACCATCCATCATGCACCGCTA

>ebv:76531-76571,numTags=955

CGAGTCAGTTACACACACAGTAGCCGAATATCTGGAGGTC

>ebv:77911-77951,numTags=8122

CCTGGGCTGGCAGTACACTGACCCTCCCTCTCGCTCGTTA

>ebv:78731-78771,numTags=6164

GTTGACATCATGAGCAAGGGCCCAGGGGAGGGACCAGGTG

>ebv:78851-78891,numTags=4624

GGTTTTACTGAACGAGCCTGTGAATCTTGCCAATAAACGT

>ebv:79611-79651,numTags=31282

TGGTGGATGTGCGAGCCATAAAGCAGTTTCTGGAGGCCAC

>ebv:8111-8151,numTags=2919

GTGTGTGTAATTTGTCCTCCAGATCGCAGCAATCGCGCCC

>ebv:81911-81951,numTags=3508

CCATTCCAGCATCATTGCATGTGTCATGGCCACCCTCTGC

>ebv:83851-83891,numTags=17146

CGTCCCTGCACAGGGGTGAGCGATGCAATGTGACTGTCTT

>ebv:84631-84671,numTags=24682

TGCCAGGGTGCTCATGAGCGAGGGCCAGATGCAGGAGCTG

>ebv:85431-85471,numTags=10161

AGAGCTGCTCGCTCAGGCTGTTCTGTGAATAGTACACTGG

>ebv:85891-85931,numTags=1904

ACGCACATGCTCAGCCAGGTAAGTCTCCCGGGTGAAGTAG

>ebv:86031-86071,numTags=3105

ATCGCTTAAAGGTTCCCTCGTTGAAGCACTGTGCGTGGGC

>ebv:86171-86211,numTags=3147

CGCAGTTTCTCGTGTGCAAAGTGGGCAAAAGCCTCGGTCT

>ebv:86531-86571,numTags=16953

AGTTCCACGAGGGGCGTGAGCGAGCGCAGGGCCCCCGCGC

>ebv:87131-87171,numTags=1680

ATTTGCTCGCTGTTCAAGCCAATAGCATCACGGATCACCT

>ebv:87251-87291,numTags=2008

GCTCAGTCATCTGGAATACCATGATCTCTCTGGCTGTAAG

>ebv:87411-87451,numTags=1181

CTGGGCGCAAGAGATCTCGGCCGTTGTCTCATCGGTGACC

>ebv:87671-87711,numTags=5409

CTCCCCTCGAGCGCCTCGCTCAGCCCACTATCACCCATGG

>ebv:971-1011,numTags=3245

ACTCATTACTTGTTCTTTTGTAATCGCAGCTCTAACTTGG

>ebv:9131-9171,numTags=9232

GTTAACAAGGGGGCCTTATAAACACTATTGCTAATGCCCT

>ebv:9271-9311,numTags=5773

GCATTGGTGTAAGAGCTTCAGCCAAGAGTTACACATAAAG

>ebv:9431-9471,numTags=3041

GTTTGGTCCCCCCCCGTGTCACATGTGGAACAGGGCCCAG

>ebv:9591-9631,numTags=3742

GCCCCACTGTGCAATGCAGCTTTTAGCCATGCCATGCTCT

>ebv:90071-90111,numTags=967

AGCGGAGGTTAGTAAAGGCATATGTGACGTTGAATTGTCA

>ebv:90991-91031,numTags=7771

TCCGATTGCTCGCAAATGCACCAGAAATATTTTCACAACC

>ebv:92191-92231,numTags=1652

AGGTACAAGGGGGGTGCGGTGTTGGTGAGTCACACTTTTG

>ebv:92671-92711,numTags=993

CATCGACACACGAGCCATAGACCAGTTTTTCGGATCCCAG

>ebv:92831-92871,numTags=909

GCCGGATGGCCTATGGGTTACCAGGCATACAGCAGCTGGA

>ebv:93211-93251,numTags=3668

TGTCAGCGATGCCCTGGGCACCACTAGTATCCAAACACCG

>ebv:93951-93991,numTags=9909

CGACAGTGGCACTTGAGCGACCAGTTTACCCCAAGCCAGT

>ebv:94271-94311,numTags=1148

AGCCAGGTTGCTGATGTGGTCCGGGCACCTGGGGTACCGG

>ebv:95871-95911,numTags=5078

CGTCACAGATGTCAGGCCATCAGAAAAAAACCATTACCAA

>ebv:95951-95991,numTags=5107

ATCCTGGCGTATGGGTTACCGCACGCATAACCTCAAAGTA

>ebv:98031-98071,numTags=5506

CCGTCTAAACGAGCGAAGATCGAGGCCTATACAGAGCCCG

>ebv:98231-98271,numTags=6163

ATCGAACGATGAGTGATTTCGCCCATGTAACAAGAACTGG

>ebv:98311-98351,numTags=5698

AGTGATAAGTCATGACAATTTTAGATGAGGTAGAAATTTT

>ebv:98391-98431,numTags=5430

GGGGACTCTAGACAGTCACCCGACAATGAGCGGGGAGATA

>ebv:98791-98831,numTags=2058

CCATTAATTTTAGCAATCGCACCTGCAAGCGCTATCAAAC

>ebv:98931-98971,numTags=2345

AGGGCGGTGTGGGTTGGCGACATTGGCTTCTAACATCTCC

Training set: **46312.QuEST.params.old.peaks.meme**

>ebv:114242-114900

CTCGGCTCCTCGGCCATTCCTGTTCACCCCACGCCGGCCTCGGTCCGACT

TTTTGAGATCCTGCAGGGAAAGTACGCCTACGTCCAGGGACAGACCATCT

ACGCCAACCTCCGCAACCCCGGAGTCTTCTCGAGGCAGGTGTTTACCCAT

TTGTTTAAACGAGCCATCTCTCATTGCACGTACGATGACGTGCTACATGA

CTGGAACAAGTTCGAGGCCTGCATCCAGAAGCGATGGCCGAGCGATGACT

CGTGTGCGAGCCGGTTTCGTGAGTCCACCTTCGAGTCGTGGTCCACGACC

ATGAAGCTGACCGTGCGTGACCTGCTGACCACCAACATCTACCGAGTGCT

ACACAGCCGCTCCGTGCTCTCCTATGAGCGTTATGTGGACTGGATCTGCG

CCACCGGCATGGTGCCCGCCGTTAAGAAGCCCATAACCCAAGAGCTCCAC

TCCAAGATAAAGAGCCTGAGGGACAGGTGCGTCTGTCGGGAATTGGGGCA

CGAGAGGACCATCAGGAGTATCGGGACGGAATTATATGAGGCAACGAAGG

AAATAATAGAGTCGCTCAACTCCACGTTCATCCCCCAGTTTACGGAGGTG

ACCATCGAGTACCTTCCGAGGAGCGACGAGTATGTGGCCTACTACTGTGG

CCGCCGCA

>ebv:106126-106579

TGCGTCTGTTTGTGTAGTGAGGTGTTGTGTCCTGTATGGTATTCTACTTT

AAAAAGGCCGGCTGACATGGATTACTGGTCTTTTATGAGCCATTGGCATG

GGCGGGACAATCGCAATATAAAACCCTGACCATCACATGGGGCATTAGGC

GACTCTGCATCAGCATCGCTTAAGTATGAGTGGGCAGCAGAGAGGCTCGG

TTATTTTGGTTCCTGAACATCTGGCTGGGGCATTAACTAAGCTTATGAGC

GATTTTATCACAGGACAAGATGTCACTCTTTCTGGAGGAAATATTGCAGT

CAAAATTCGCGATGCTATAAACCAGACCCCCGGGGGTGGTGATGTAGCTA

TACTTTCTTCCCTGTTTGCTTTATGGAATGCCCTCCCAACATCTGGTAGA

CAATCCTCCAGGGACGATTTAATCCCAGCCGCCGTGCAGGCCTTAACCAC

GGC

>ebv:79396-79867

CCTGCTGCGGGTCCGGGGCCTAATGCCTGGCATCTGCCTGGCCAATAACT

ACATAAGTAGGGATGAGCTGCTCCACACCCGCGCTGCCTCCCTGTTATAC

AATAGCATGACAGCCAAGGCTGACCGACCAAGGGCCACCTGGATCCAGGA

GCTGTTTCGCACTGCGGTGGAGGTAGAGACTGCCTTCATCGAGGCTCGTG

GAGAGGGGGTTACCTTGGTGGATGTGCGAGCCATAAAGCAGTTTCTGGAG

GCCACGGCCGATCGCATCCTGGGTGACATTGGTCAGGCTCCCTTGTATGG

CACACCACCCCCCAAGGACTGCCCGCTCACCTACATGACTAGCATCAAGC

AAACTAATTTCTTTGAGCAAGAGAGTTCCGATTACACCATGCTGGTGGTA

GATGACCTTTGAGTCAGGGTGGCTACTTGCTCAGGTTTCTGGGCATAAAT

TCTCCTGCCTGCCTCTGCTCT

>ebv:86005-86756

TTCTCCCATGCTCGTTAACTGAGAGTATCGCTTAAAGGTTCCCTCGTTGA

AGCACTGTGCGTGGGCCAAATAGACGTAGCGCACGAGATCGGCCGAGGCC

AGGGGAAGGCGCCCCCTGTAGGCGTCTATCGTCCTTGCCACAGCGCGGAT

CTCTCGCGAGTCCCGCCGCAGTTTCTCGTGTGCAAAGTGGGCAAAAGCCT

CGGTCTGCTCCGCCCATGCCGAGGAGCCAAAGACCTCCCCCAGCTCGGCC

AGGGACGTGACGGCGGCCAGGCTCTGACCAGACTCGGAAGTAAATAGCTC

CGTGAGGTGCGCCAGGGTCTCAATCGTACAAGGAATGCCCCAAAAATAGT

AAGCAGCCGTGACTAGCACGAACTGGGCCTCGTGGGAGCCAAAGGTGCTA

ATGAACCACCTGGCCGAGATGTTAACGCGGTAGATGCGGCGCAGACAGCC

CACGATCTTGGGACGCAGCCACGCCACGCGGCCTCTGGCATCCCCCTGTG

GCTGTTTCTTAGCGCTCAGTGTGAGCAGTTCCACGAGGGGCGTGAGCGAG

CGCAGGGCCCCCGCGCGATCTAGGTAGGTGGATAGACGGTCCGCGGTGAG

CGGCGTGAGGCCGCGCAGGAAGGGGAAGGCCTCCTCCGCCGGCAGGTGCA

GCGTCAGAACCAGGCCGCAGCGGCTCTGTGAGGTCAGCCGCTTCTTGGGC

AGGTGAAGCTGCAGTTCCACGAGAGAACCCGCCACGTGGTGGAGGGGCGA

G

>ebv:177171-177615

ACACAGAGTACCACCAGGAGTAGTCTTAGTCTGCTGACGTCTGGGTCCTC

GGGGCAGGGGTGGCTAGGCCTGGTCTCCGTAGAAGAGCCGGGCAGGCCGC

AGGCAGAGGACTGCTGCTCTAGCAAAGCACGCTCCAGGACGTGTACCATC

TCGAGAGTGAGGCACAGCTGTTTTCGTGGACTTTTATACAGTAAGGACAA

GGAAAGAAGGCCAGAGGAATGTGGAAAGATGAGCGAGGACAGGTGTGGAG

GTTTTGGGCTAGCTCTTAGTTTCTGGGTGTGAGAGAGGGATTAAAGTGCT

TATGCGCAAAGAATGTGTCAACAACAGGTGTTCCTGCCTCTGCTGGCATG

AGTTAGGTGTGGCTTGGGCTGAATCCAAATGTGTATTGGCACAAGATGGA

AAGCAAAGTTGCTGGAGTTACTGGGTGGGAGACAGGGATGTATG

>ebv:126570-126986

CAGGCTGTGTGGCCCCTGGACTGCCTTAGTGAAGAGAACCTCGGGCCTGG

CCACGGTGGCTAGGGTTCCATTGATGTAGACGGTCACATAGGTGGGCTTC

TTCTTGGGCTTCAGCACAATGAGGGTAACATTCATGTAGGTTTTAGGAGG

TCCGGCTATCTGAGGCACGTACACAGCTGACACGGCGGTTGTGGCCGTAT

AGACTTTCATCTGGGGCGTAGAGGCATCGCTCAGCACCCAGAGGCACTCC

TTGTTGAGGAACTTGCGAAGCTGTTCCCGGCTACTGTTCGCGGCGGATGC

CATGACGTGCCAGAATATATCCCCTCTCCTCGGGGGTGAGTGCCAATTGG

CCTTTAATAACAAAGCCCCCAGGCAGCACCAAAAATGCCTGCCCGTCCGA

TGTGGTGGCCAGGTGG

>ebv:164807-165214

GCTCGGGTAGAGGCTGGCAAAGTCCACCACCAGAACCGGGGAGTTGTAGA

ATCCGGACAGGGGCTGGATGACGGTGGCCCCCTGGTAGCCGTCCCGGTCA

GAGGCCGAGGGCATGGGCAGGATAAAGTTTTCCTTTTGGGCGGCCGCCAG

GAGGCAGGAGAACACGCGGATCTGCTGCCCATCGTCCAGCACCCGCCTGC

AGGGGATGTGAGCGATCTTGGCAATCTCTGCCACCTCCACGTGGATCACG

AAATGGTTTAGCAGATCCATGACCAGGGCCGAGTCCTGCACGCAGTACAT

GCCGAGCCGCCTGCGCCCCTCGGGGCCCGCTGCAAAGAGGCGAGGAATCT

CCTTGTAATGCACATCCTCCTTCTTGGCCCCCAGTAGGTGCCTGGCTACT

GTGTCCA

>ebv:109902-110308

CGTATTAAAAGCCGTGTATTCCCCCGCACTAAAGAATAAATCCCCAGTAG

ACATCATGCGTGCTGTTGGTGTATTTCTGGCCATCTGTCTTGTCACCATT

TTCGTCCTCCCAACATGGGGCAATTGGGCATACCCATGTTGTCACGTCAC

TCAGCTCCGCGCTCAACACCTTCTCGCGTTGGAAAACATTAGCGACATTT

ACCTGGTGAGCAATCAGACATGCGACGGCTTTAGCCTGGCCTCCTTAAAT

TCACCTAAGAATGGGAGCAACCAGCTGGTCATCAGCCGCTGCGCAAACGG

ACTCAACGTGGTCTCCTTCTTTATCTCCATCCTGAAGCGAAGCAGCTCCG

CCCTCACGGGCCATCTCCGTGAGTTGTTAACCACCCTGGAGACTCTTTAC

GGTTCA

>ebv:161935-162298

TCTGGTCCCTATCTCCACCCGCCAGGCCTGTGTGTCAGTTTGCAGGGCCA

TCCTCGCACTCAGGTGGACTGGCTAGGCACCCTTCTGAAGTATCTGGCGG

TGACTGTCACCTGGTTCTTGAGAGAGTCCATAAAATGGCTGAAGCTCCAG

GCGTATAGTATAATGAGCAACAGGGCCAAACAGGCGGCGGGGCCTGGGTA

GTAGCGGGCAACGAGAGACTCTGTGCAATCAAACCCCAGGCTCCCGGCCT

CACCCAGGAAGAGCAGCGGCAGGGACAGCATAAACCAGGAGAAGGCGCAA

ATGAGTCCGGTGAAGGTGACGTTGCATATCAGGCGCGGCTTCCTTCCGAA

TTTTGTGCGCAAA

>ebv:84350-84802

TTTATGATTCTGGAGGCGGGCACGCTGATGGAGAAATGGGCGGTCGGTTG

ATTGGCCCCACAGCGACCGGCGAAGCACTGACTCATGAAGGTGACCGTGA

TGGCCTGTGATGTGTAGTAGAGTACCAGAAACACCCTCACATTCTTGGAG

CTGGCCCTGTGGGTATGCCTCAGGCACGCAAAGTTCCTGCCCCGGGCATG

GCACACCTGAACTAAGTTTGGCCCGGTTTGCTCAAACGTGACATGGAGAA

ACTGGGGGAATTTGTCTTCTGGCACAGCTGTTGCCAGGGTGCTCATGAGC

GAGGGCCAGATGCAGGAGCTGACCCAGGCGACGAGATCCAGGCCCAGATG

TCCCTCTATCATGGCGCAGACATTCTCCACGGTGGGGGGCAGGGTCTCGC

GGGTCCTCTGGATTAGATAGTCACGCCCATCATCCGCGATGTGGTAGCAG

AA

>ebv:83676-84041

GGAGACCTCGGTGCTCTAGGCACCCTGGGGGCCCTTGGGGCTCTGGGCGC

TCTTGCTCCCGGGGGCAGGTGTCGGCGCTTGCCATAACTTTCATCGGTGC

AGCCATGGACCTCTCCGCGTCGCCTTTTGTGGCCTCTGGTGTAAGAGGAG

TTGCCAGTCTCCTCCTTCTCGTCCTCGTCCCTGCACAGGGGTGAGCGATG

CAATGTGACTGTCTTGTCCTGTAGGTCCCACTTCTTTCTGGGAATCACAA

ACGATGCCGAGGTAGGGGTTATGACCACGCTGGAGGGCCGTGCAGGTATG

GCGTGGGCCGGAGTTGGATCTTCATCCTCCTCCTCTGAGGATGAAATCTC

TCCATCTGTGGAGTG

>ebv:166640-166967

CGTCTTCTGCGTAGGCCTGGAAAACGGTCCCTGGCTGCCTGCAATGCTCT

GCTGGCCACTGAGGGTCCGGCCGCCCTCTGAGCTGCTCTCTTTTGCTCCT

GGTTTTGCTCATGCAGCGCTAACATGATGGCTTGTAATTCTGTCTTACTA

ATGGGATTAATGCCTGGACCCTCACCAGAGGCATGTTGCTGAGCGAGCTC

GTCGATCCCGGGGTAGAGCATCTGCACCGGCTGCTGCGACATCTGGCGCG

TGCGCCTCGTGAGGGAAATAACCAGGATCACCACCCCCGCCACCAGGACC

AGAATGAGCATGCCGCCGAAGGGGTTT

>ebv:110629-111123

ATCACGGGGGTCAAGCAAACGGCCTGGCATTCAGCGTCGCATACGGCTTT

CCAGTTCCCCCCAGCCTGAGGAACATCTACGCGGAGCTGCACCGGAGCCT

GCCGGAGTTTTCTCCCCCAGATCACGGCTGTCTAGACGCGTGGGCCTCCC

AGGGGGTGTTGCTACTCAACACCATCCTGACCGTGCAAAAGGGCAAGCCC

GGCTCGCACGCAGACATTGGCTGGGCGTGGTTTACTGACCACGTAATTTC

ATTGCTCTCTGAGCGGTTAAAAGCGTGCGTGTTTATGCTGTGGGGTGCGA

AGGCGGGAGACAAAGCTTCACTAATCAACTCCAAGAAGCATCTGGTTCTG

ACCTCTCAGCATCCCTCTCCCCTGGCCCAGAACAGCACCCGAAAGAGTGC

CCAGCAGAAGTTCCTGGGCAACAACCACTTTGTCCTCGCTAACAACTTTT

TGCGTGAGAAGGGGCTCGGTGAGATAGATTGGAGGCTGTAGAGG

>ebv:66442-66774

GGCCTGGAGGAGATCGTCCCGCTCTCGCCTTGTCAGCAGCTGGGGCTTCT

TAGGCCAGAGCGCCGAGTCCGAGGCCAGCCTGGGCGGGGCGGTTGCCTGG

GGGATAGTTGGAGGAGGAGGCAGGTTAGCCTGGCCTGGGTCATTAGTGGC

TTCGGGTAGCGTCCGATCCACGTACTCGCTCACGATGGCCGTCAGGGCAG

CCTCGGCTGCTCGTCTTTTTTCCAGAAGCCCGGCCAGCCCCCGCTCGTAC

TCCGCGTAGGGGGCCTCCAGATCCGTGTTGACCACCGCTGATTTCATGTC

CGGGGACTGCAGGGCCTGGCGCGTCTGCGCGA

>ebv:147967-148363

GCGCCCGGACGCCCCACCCAAGGACGCCTGCCTTCACCTGGATCCCCTCA

CCGTCAAGAGCCAGCTCCCTCTGAAGAAGCCCTTGCCACTCACGGTGGAA

CACCTGCCGGATGCTCCGGTCGGCTCAGTCTTTGGCCTTTACCAGAGCCG

AGCGGGTCTCTTTAGCGCAGCCTCGATTACCTCTGGGGACTTCCTGTCCC

TGCTGGACTCAATTTACCACGATTGCGATATTGCACAGAGTCAGCGCCTG

CCCCTCCCTCGAGAACCCAAGGTGGAGGCTCTGCACGCCTGGCTCCCCTC

ACTGTCACTGGCCTCCCTCCACCCAGACATACCCCAAACCACCGCAGATG

GAGGCAAGCTGTCCTTCTTTGACCACGTGTCTATCTGTGCCCTGGG

>ebv:145053-145344

ACTATTTTTCGAAGTCACCCTGGGTCGGAGAATCGCAGATTGCGTTGTGG

TAGCTCTGCAGCCTTATCCCCGGTGTTATATTGTAGAATTTAAGACGGCC

ATGAGCAACACGGCCAACCCGCAAAGCGTTACTCGCAAGGCACAGAGGCT

AGAGGGCACCGCCCAGTTGTGTGACTGTGCCAATTTTCTTCGCACGTCCT

GCCCCCCCGTGCTGGGCAGTCAGGGCCTGGAAGTCTTGGCGGCGTTGGTA

TTTAAAAACCAGCGATCCCTGAGAACGCTCCAGGTAGAGTT

>ebv:67792-68122

GACCGGCAAGTCACGATAGAGGTGGTCTATGCTCTCCTCGAAGGAGGCAA

TGTAGTTATCGATGGTGTAGAAGGTGATGGATTTCAGGATGTTCATCAGG

TACTTTTTGGAGCGAACAATCTGCTGTATAGTGTCACGTAGGCGGATGTA

CGTGGGGTTCTTTGCGGCCCCGACTATCGACCCTGCATTTGCGATGTACT

TTTCTATGACGGGGATGGTGAGGGCCGCGGTGTCGGCCAGCGGTGGCGTG

GCTTCGGGGTTGTCGTGGTTGGCGGGTGTCGCAGAGGGAGAGGCGGGAGA

GATGGGGGCGCCTGGGGCCGAGGCCACACC

>ebv:53437-53839

TGGCACCCCGCGTGCCTTACTGACTTGTCACCTTTGCACATTTGGTCAGC

TGACCGATGCTCGCCACTTCCTGGGTCATGACCTGGCCTGTGCCTTGTCC

CGTGGACAATGTCCCTCCAGCGTGGTGGCTGCCTTTGGGATGCATCACTT

TGAGCCACTAAGCCCCCGTTGCTCGCCTTGCCTGCCTCACCATGACACAC

TAAGCCCCTGCTAATCCATGAGCCCCGCCTTTAGGAAGCACCACGTCCCG

GGGACGGAAGGGGACTTGGGGTGATTTTCTATGTGGGGGTGGAAATATGA

GCAAGAATAAGGACGGCTCCTTATTAACCTGATCAGCCCCGGAGTTGCCT

GTTTCATCACTAACCCCGGGCCTGAAGAGGTTGACAAGAAGGGTCAAGGT

TT

>ebv:176541-176815

CGTGAATCTAATGAAGAGCCCCCACCGCCTTATGAGGACCCATATTGGGG

CAATGGCGACCGTCACTCGGACTATCAACCACTAGGAACCCAAGATCAAA

GTCTGTACTTGGGATTGCAACACGACGGGAATGACGGGCTCCCTCCCCCT

CCCTACTCTCCACGGGATGACTCATCTCAACACATATACGAAGAAGCGGG

CAGAGGAAGGTAAGAGTGCCATCTATCTGTACTTTTATTTATTGCATCAC

AAGTCACATCAATAATAAGGGCGC

>ebv:122344-122869

GACCAAAGAAGTCGTCTGCCAAGAGTTCAGCTACCAGACCTGGAAGATGA

GGGTGCTCAAACCGTGGGCGACAGTTGAAGAAGTAGCTCTCCTTGAACCT

CTTTTTAAGGCTCCGGCACCACTGCAAGAATTGACTCATATGCTCCGCCG

TGACATCCACGCACGGACTCTCGCCACACGAGGTCAGGCCCATGTCTAAG

TTCAGGTTCCACATCTGCGACAGCACCTCCAACAGCACCACCTTTGGGGC

TGCAAATTGCAAAAAGTAGAGCGGGTCGGATCGGTCAAATCCCATGTCAG

GGTTGGGGTAGGGGATTTTGTGGGTGGAGTCAGCGAGGTGCATGATACCA

TAGAGCAGCGAGTAGCCGAGCGACTGCAGATCCAGGCGAAGGGCCGTCTG

CGCCCCCACGGGGCCACACGCCGAGGGGTCAGGGATGTGCCCAGCCCCCC

TCAAGATGTAGCACTTGCTCAAAAGGCAGAGGGGCTTATAGGTGTCCTTG

GCTATAGAAAATGGTTCCCTCTGGC

>ebv:49077-49424

AACCACTCATGATGCCACCAAGGCCTACCCCTCCTACCCCTCTGCCACCT

GCAACACTAACGGTGCCACCAAGGCCTACCCGTCCTACCACTCTGCCACC

CACACCACTACTCACGGTACTACAAAGGCCTACCGAACTTCAACCCACAC

CATCACCACCACGCATGCATCTCCCTGTCTTGCATGTGCCAGACCAATCA

ATGCACCCTCTTACTCATCAAAGCACCCCAAATGATCCAGATAGTCCAGA

ACCACGGTCCCCGACTGTATTTTATAACATTCCACCTATGCCATTACCCC

CCTCACAATTGCCACCACCAGCAGCACCAGCACAGCCACCTCCAGGG

>ebv:56613-56899

GGGTGTTCATCTCACGCAGAAAATCTTTGCCCAGCTCAAAGTTGGCAGAG

ATTCCCCTGAAGAAGTCCCGTAGTGAAAAATGGGATCTGTCTACACCATG

TCTGGTGTGCCGGGAACATATTGATCGGGCCACACTGCCAACCCTTTCCA

TTCTTCCCAGCTCTGAGCGAGATTTTCCACACCTGGACACCGACTTCACG

CTATGCGCCGAGGCCTTTGAGGCCGTGTAGTTTCTGTGGTGCGGATGCAT

TAGGCGGCGCAATGCGGGATCTGCCGGTCGCTGTTG

>ebv:119625-119934

TCAGTTCTACACGCCTGCGCATGTGTGACCCGCTTCTCCCCGGTCTGGGT

GGTCAAGGCCCAGGACAACTCTATTCCCCAGGACACCTTCTTGTGGTGGG

TGGTCTTCTACCTGAAGCCCGTAGTTACAAACCTGTACCTGGGGTGCCTT

GCCCTGGAGACGCTGGTCTTCTCGCTCAGCGTGTTCCTGGCCCTGGGCAA

CAGCTTTTACTTTATGGTGGGGGACATGGTGCTGGGAGCCGTGAACCTCT

TCCTCATCCTGCCCATCTTCTGGTACATTCTGACGGAGGTGTGGCTGGCC

TCCTTCCTG

>ebv:85292-85589

AGTACCTCACGCCGCACGGTCCGGCACAGGTCATAGATTTCCTCCAGCGA

CCAGGGGGCCCCCTTGATCTTTAGATCCAGGTCCAAGACCAGGTTGCAGA

CCGGAAGCCGGGGATTAAAGTATTCATGCCGGGAGACAAAGAGCTGCTCG

CTCAGGCTGTTCTGTGAATAGTACACTGGGGTGTAGGAGAGGGCCCTGGT

GAGACACGTGTCTGGGAGGCGGCAGTTGGTCGGGGTGGAGACGACCTCCG

CCAGGTGGGATGAGAAGGGGTCAGCGGCTGTCATTACAAAGTAGTGC

>ebv:52737-53008

CGCCGGGCCGGCTGGGAGGTGTGCACCCCCCGAGCGTCTGGACGACGCTG

GCGAGCCGGGCCGGCTCGCCTTCTTTTATCCTCTTTTTGGGGTCTCTGTG

TAATACTTTAAGGTTTGCTCAGGAGTGGGGGCTTCTTATTGGTTAATTCA

GGTGTGTCATTTTAGCCCGTTGGGTTTCATTAAGGTGTGTCACCAGGTGG

GTGGTACCTGGAGGTTATTCTATTGGGATAACGAGAGGAGGAGGGGCTAG

AGGTCCGCGAGATTTGGGGTA

>ebv:64473-64710

GGAGCTCTTCTCCGCGTGTATAGGCCTGCTTCACAAAGTCGCGCAGGTAG

TCCTGAAATGCGACCGGGCCCTCCAGCGGGCGCAATGAGTGCCAGAGCTG

CTGAAGGGCCTCGGGGGCGAAGCACCGGCGTGCGAGGAGCAGCATGCAGG

CTCGGGCGCGGGCCGTACTTTGGTTGTGGACCAGGCCCAAGAACTCGGGG

TGCGGCCAGAGGGCGGCTCGGGTATCCATCTCCTCCC

>ebv:9107-9270

GGAAGCATATGCTACCCGTTTAGGGTTAACAAGGGGGCCTTATAAACACT

ATTGCTAATGCCCTCTTGAGGGTCCGCTTATCGGTAGCTACACAGGCCCC

TCTGATTGACGTTGGTGTAGCCTCCCGTAGTCTTCCTGGGCCCCTGGGAG

GTACATGTCCCCC

>ebv:127346-127560

ATGAGGAAGCGAAACTCCCTCTCTGGAGCTCCCAAAATTGAAACCTCAGC

AAGATCTGTTGCTGGGGAGGCATGGGTGACAGCTGTCATCCTGTGCAGTC

TGCCCTGGGCACTCAGCTCTGGATATGTGACAACATAGAGAGCGTGGGGG

CTAAAAATATGAGCAATTCCCCTGACCAGGGCCCTGGACTCACGAATGGC

CCGACGGGTCTTAG

>ebv:150337-150695

TGTCCATCCCCGGGAGTGTATCCCCGGCCCAACTCGATCCGAGAGACCGA

CTCATTGCTAGGAACGCTGCAGGTGAGATTCACTCTAGCACCTGCATGGG

CGGTGACATTTTCAAATTTAACCAGATCTGAGAAAAATGCACAAACAGAC

CCCACACAGCAGCACAATAGAAGCACTAAATGAGTCATTCCTAAACTGTC

AGTTTTAAAACTCCCTGCTTCTCAGGCCTAAATATGTGGTGGGGTGTGCT

TAGGATCACTTTCATATTCTGCAACAACAGCCATACCCGGAAGAGGAGCT

GCCGGTTGCCATTTTTCAAGCTGCTAAACCACGAGTGGCAGCAGGCCTAA

GAAGCTCC

>ebv:147010-147325

CTCGCCCATGCTTGCGGCGCGGGAGATCCTGGAGCGTCAGTTCTCAGCCT

CGGATGACTACGACCGGCTGTACTTCCTGACGCTGGGCTACCTGGCCTCC

CCGGTGGCCCCAAGCTGAGCCAGTTCCTCGCACTGGAGTGGGTCATTGGC

AAAAAGGTAAATAAACTCATCGCACGGGGGTTTTGCCTCCTTCTCGTCTC

TTGTTTCGGGTAGGGGAGTAAGGCCGCTGCCAGGCCGCCATGCTCAGGGC

CACGGCGTGCCAGAGGCCCTCGTAGTCGTGCGCATCCGAGAGGATGGCAC

GGTCCAGAAGCAGAT

>ebv:150724-150976

TGGCAGGAGCAGGGAGTCACGTAGGCACTAGCCTCTTCATGTGAGGTAAG

AGATCGCTAAAAATGGGATCAGGGTATGTAAACCGAGTTTTGCGGGGGAT

GGTGAGCCAGACACGGCGGGTGGGGGAAGGAGCTGACACGATTGAGTAGA

AAGGGCCAAAAATACACCAGCTATAAGGAATTGCTCAGGCCAAAGTTGTT

CCTCAGGTGGCTTTAGGCCTAATGTAGGCAATTGCGTGCCTAGAACATTG

CT

>ebv:2440-2626

TCCGCGCTGCGAGACACTTACTCGGGCCACTTGGTCCTTCAGCCCACGGA

GACCCTTGACACATGGAAGGTGTTGAGCAGGGACACACGAACCGCTCATA

GTTTGGAGCACGGATTCATTCATGCCGCGGGGACCATCCAGGCCAACTGC

CCACAGCTGTTTATGAGACGCCAGCACCCCGGCCTC

>ebv:118717-118899

TCTAAATGAAAGGTCAAATAGGTCCTTCTCGGCGGCATCGGCGAGCATAG

CAATGAGCCCCCCGCTGCGCCTGAGCTCCCGCTCCCATCGCAAAAAGTTG

AGTTCGGTAGTCGAGGGCGCGTTGACCACGGGGGGCTCCAGGGAGCCTCC

AAGCGGCGGCTGGCAGGCCTGCACCACGATCA

>ebv:87610-87796

ATTGGTAAAACAAGGGATATGAAGGTGTCATTGACCCGAGGATCCAAACC

CCCTCCCCTGTCTCCCCTCGAGCGCCTCGCTCAGCCCACTATCACCCATG

GCCAGGCTCGGCACCTCCTCGAAGGTGCAGCTGGCCCACCTAAAGAGAGA

TCTGGGGCCAAGGACCCCCGCGTCACTGTGGGGGCT

>ebv:129518-129739

GCAACATCCGAGCAACCACCAACCTCTTTCTGGACGGGGCCTTTAGCACC

GAGCTGATGGGTGACACCTCCTCGCTGAGCCAGGGTAGCCTGAGCCGCAC

TGTGCGTGACGATGCCATCAACCAGCTGGAGCTCTGCCGGGTTGACACCC

TCAACCCCCGAGTAGCCGGACGCCTAGCCTCCTCCCTCTACGTGTACGTT

GATCCGGCCTATACCAACAAC

>ebv:137062-137258

ATGGACAGTTCCATTTCCGTACTAATGTGGTGTTTGTGGCAATTTTTGAC

CACAATGAATGTCCGCTGCTTGCTGGGTCTCCTTCCGTCCCCGTGAGCAA

TGGTGGGGACGGAGATTCGAAATTGAATCTTGCCATCCGTCATACGACTC

AGGTCTTTGAATTCCGTGTTCACACAGGACACGGCCAGTGCCGTCT

>ebv:4263-4402

TGAGCAGCTCTTTAGCGACGTGGCCACGACCCCACGGGCACCCGAGGCGT

TGTCTCTGAAGAATCTCTTCCGGGCAGTCCAGCAGCTGGTCAAGAGCGGC

ATCGTGCTGTCAGGGCATGACATCAGCGACGGGGGCCTG

>ebv:147487-147715

GGGTGACCTGGCTCAGGTCCGGCGCCCTGCGAGTCCAAGCTGCGCCCACA

CACATGCACAGACGGCCCCTGTGACATCAGGCCGGTCATGCAAAAACAGA

CAAAGAGACCGTGAGCGGTTACCGGGGCGCAGGGCCTCTGCCGGGAAGCC

CACCCGGGCCAGGGCCCGGTAAAGCAGGTACCAGTATTCATCCGGCACCT

TGCGTGCCAACACACGATTCGTGCGGTT

>ebv:77811-77973

TGGTGGCCTGCTCCATTCTTCAGGGATCCCCCACTTATGATTCCAGGGAT

ATGGCCTCCATGGGCCTCGGGGTGCAGGGCCTGGCCGATGTCTTTGCGGA

CCTGGGCTGGCAGTACACTGACCCTCCCTCTCGCTCGTTAAACAAGGAAA

TATTCGAACATA

>ebv:90892-91091

ATATAACCTTGTGGGTTGTTGTGGTGGCATTGGTAGCCGTTCGTGTGATA

ATGAGTGTCTTGGGGGCCGTGCCAAGACCCGAGACAGTAATGTCAAATGT

CCGATTGCTCGCAAATGCACCAGAAATATTTTCACAACCCGAAGGTGTCC

CCGAGGTGAGAGTCCATTTGCACTTAAAGTCAGTTTCAGTGTTGTTTGG

>ebv:172693-172866

ATAGCACATACAGATGGGCAGGGAGATGTCCTGCAGGATGGTCAGCAGTG

AGCGGTAAAACAGCTGGGTGAAGATGGGGCAGGCGGGCTGCGCAAAGGGG

TTGCACGAGTACTGCATCACGTGGTAGCAGCTCTTGACCAGGTCCTTGTA

GGTGATGTTGTTCTTGGCCATGC

>ebv:97959-98189

GTGACACAGGCACCCACGGAATATACCAGGGAAAGGAGGGGAGTGGGGCC

TATGCCTCCCACCGATATTCCGCCGTCTAAACGAGCGAAGATCGAGGCCT

ATACAGAGCCCGAGATGCCGCACGGGGGGGCCTCGCACTCTCCCGTCGTT

ATCTTGGAGAATGTCGGCCAGGGGCAACAGCAGACTCTGGAGTGCGGAGG

AACTGCTAAACAGGAAAGGGACATGTTGGG

>ebv:136661-136793

TCGTGTAGGTACTGACCCCCTTGAGCACGCTCTCCCCGGACACGGCCGCT

ACCATCTCAGAGAGACGGCTTCGCACGTACTGAGAAAACCCGGAGCCCAT

GTTCTCGGCCCGGTCCAGGAAGAAGGAGTGCT

>ebv:78693-78782

CGCCTCGTCCCATGGAACCGGCGCAGGTTGCGGGGCCGGTTGACATCATG

AGCAAGGGCCCAGGGGAGGGACCAGGTGGGTGGTGTGTG

>ebv:61370-61478

GCCGTTTGCTCCATCTGTCAGCAACGCCAAGCTCCAGAGGACGCCCGCGA

CGAGCCTCACCTGTTCTCCTCCTGTTTAGAGGTAGAATTGCCACCTGGTG

AGCGGTGT

>ebv:58438-58517

TACGCCACTCCTCGTGAGGGGGCAGCTGGACAGTCTTTTTCCGGTCAGGG

GGTTTGGCTCGTTTGCGCTCGTGACTTTG

Training set: **GFP95.8.SISSRs.out.meme**

>ebv:106351-106391,numTags=2230

TGGGGCATTAACTAAGCTTATGAGCGATTTTATCACAGGA

>ebv:110091-110131,numTags=2793

TAGCGACATTTACCTGGTGAGCAATCAGACATGCGACGGC

>ebv:110831-110871,numTags=1122

CTCGCACGCAGACATTGGCTGGGCGTGGTTTACTGACCAC

>ebv:114491-114531,numTags=6774

TCGTGTGCGAGCCGGTTTCGTGAGTCCACCTTCGAGTCGT

>ebv:119791-119831,numTags=1199

TCTTCTCGCTCAGCGTGTTCCTGGCCCTGGGCAACAGCTT

>ebv:122471-122511,numTags=1528

GAATTGACTCATATGCTCCGCCGTGACATCCACGCACGGA

>ebv:126791-126831,numTags=2404

AGGCATCGCTCAGCACCCAGAGGCACTCCTTGTTGAGGAA

>ebv:127491-127531,numTags=2810

GGGGGCTAAAAATATGAGCAATTCCCCTGACCAGGGCCCT

>ebv:129591-129631,numTags=907

GCTGAGCCAGGGTAGCCTGAGCCGCACTGTGCGTGACGAT

>ebv:129771-129811,numTags=989

GACTCACGACAGGGCGGACCCTAACAGGGTCATCGTCCTG

>ebv:131351-131391,numTags=933

CACCCCATCAAAGACATAATATGTCTCATAGTGGCAGTGA

>ebv:137151-137191,numTags=1551

CCCGTGAGCAATGGTGGGGACGGAGATTCGAAATTGAATC

>ebv:145211-145251,numTags=2904

CCGCCCAGTTGTGTGACTGTGCCAATTTTCTTCGCACGTC

>ebv:148191-148231,numTags=3591

GCGATATTGCACAGAGTCAGCGCCTGCCCCTCCCTCGAGA

>ebv:150511-150551,numTags=2239

ACTAAATGAGTCATTCCTAAACTGTCAGTTTTAAAACTCC

>ebv:150631-150671,numTags=1325

GGAGCTGCCGGTTGCCATTTTTCAAGCTGCTAAACCACGA

>ebv:150951-150991,numTags=1404

GCAATTGCGTGCCTAGAACATTGCTAATGTGCCCTGGGTT

>ebv:162131-162171,numTags=5334

GGTAGTAGCGGGCAACGAGAGACTCTGTGCAATCAAACCC

>ebv:165011-165051,numTags=3536

GATGTGAGCGATCTTGGCAATCTCTGCCACCTCCACGTGG

>ebv:166811-166851,numTags=1733

TCACCAGAGGCATGTTGCTGAGCGAGCTCGTCGATCCCGG

>ebv:174751-174791,numTags=2662

CAGCGATCTTGGCATGCCGCCCAGCCTCGCAAACCAGAGT

>ebv:175331-175371,numTags=3981

AGAGCAATGGCCAGGTTCATCGCTCAGCTCCTCCTGTTGG

>ebv:176691-176731,numTags=6005

CCCTACTCTCCACGGGATGACTCATCTCAACACATATACG

>ebv:177391-177431,numTags=2730

GTGGAAAGATGAGCGAGGACAGGTGTGGAGGTTTTGGGCT

>ebv:49251-49291,numTags=3030

CTGTCTTGCATGTGCCAGACCAATCAATGCACCCTCTTAC

>ebv:52831-52871,numTags=1118

TCTGTGTAATACTTTAAGGTTTGCTCAGGAGTGGGGGCTT

>ebv:53611-53651,numTags=5312

GCCTTGCCTGCCTCACCATGACACACTAAGCCCCTGCTAA

>ebv:53711-53751,numTags=5347

TTTTCTATGTGGGGGTGGAAATATGAGCAAGAATAAGGAC

>ebv:56771-56811,numTags=1924

AGCTCTGAGCGAGATTTTCCACACCTGGACACCGACTTCA

>ebv:58491-58531,numTags=1022

TTGGCTCGTTTGCGCTCGTGACTTTGTGAGCCATGACACA

>ebv:64591-64631,numTags=1257

GAAGCACCGGCGTGCGAGGAGCAGCATGCAGGCTCGGGCG

>ebv:66611-66651,numTags=1661

ACGTACTCGCTCACGATGGCCGTCAGGGCAGCCTCGGCTG

>ebv:67951-67991,numTags=1517

CTTTGCGGCCCCGACTATCGACCCTGCATTTGCGATGTAC

>ebv:71511-71551,numTags=1366

AGTCGCCGTTACTCATCTTCGGCGGTGGGGCGGGGAGGAC

>ebv:72411-72451,numTags=1895

AACAGGCGGGCGAATGTGTAATCCCGGAACCGGTAGGCGA

>ebv:76111-76151,numTags=858

TCTTAAGAATTTATTACTCACCATCCATCATGCACCGCTA

>ebv:78731-78771,numTags=1534

GTTGACATCATGAGCAAGGGCCCAGGGGAGGGACCAGGTG

>ebv:79631-79671,numTags=4860

AAGCAGTTTCTGGAGGCCACGGCCGATCGCATCCTGGGTG

>ebv:83851-83891,numTags=2258

CGTCCCTGCACAGGGGTGAGCGATGCAATGTGACTGTCTT

>ebv:84631-84671,numTags=3441

TGCCAGGGTGCTCATGAGCGAGGGCCAGATGCAGGAGCTG

>ebv:86531-86571,numTags=2980

AGTTCCACGAGGGGCGTGAGCGAGCGCAGGGCCCCCGCGC

>ebv:9151-9191,numTags=1155

AACACTATTGCTAATGCCCTCTTGAGGGTCCGCTTATCGG

********************************************************************************

MEME - Motif discovery tool

********************************************************************************

MEME version 4.4.0 (Release date: Tue Apr 27 10:09:30 EST 2010)

For further information on how to interpret these results or to get

a copy of the MEME software please access http://meme.nbcr.net.

This file may be used as input to the MAST algorithm for searching

sequence databases for matches to groups of motifs. MAST is available

for interactive use and downloading at http://meme.nbcr.net.

********************************************************************************

********************************************************************************

REFERENCE

********************************************************************************

If you use this program in your research, please cite:

Timothy L. Bailey and Charles Elkan,

"Fitting a mixture model by expectation maximization to discover

motifs in biopolymers", Proceedings of the Second International

Conference on Intelligent Systems for Molecular Biology, pp. 28-36,

AAAI Press, Menlo Park, California, 1994.

********************************************************************************

********************************************************************************

TRAINING SET

********************************************************************************

DATAFILE= **meme_w:o_F-factor.txt**

ALPHABET= ACGT

Sequence name Weight Length Sequence name Weight Length

------------- ------ ------ ------------- ------ ------

ebv:1051-1091,numTags=14 1.0000 40 ebv:1111-1151,numTags=17 1.0000 40

ebv:1271-1311,numTags=16 1.0000 40 ebv:1891-1931,numTags=67 1.0000 40

ebv:10151-10191,numTags= 1.0000 40 ebv:101151-101191,numTag 1.0000 40

ebv:101451-101491,numTag 1.0000 40 ebv:103291-103331,numTag 1.0000 40

ebv:105351-105391,numTag 1.0000 40 ebv:106211-106251,numTag 1.0000 40

ebv:110091-110131,numTag 1.0000 40 ebv:113951-113991,numTag 1.0000 40

ebv:114471-114511,numTag 1.0000 40 ebv:115651-115691,numTag 1.0000 40

ebv:116431-116471,numTag 1.0000 40 ebv:119091-119131,numTag 1.0000 40

ebv:120531-120571,numTag 1.0000 40 ebv:121991-122031,numTag 1.0000 40

ebv:122471-122511,numTag 1.0000 40 ebv:122791-122831,numTag 1.0000 40

ebv:122931-122971,numTag 1.0000 40 ebv:123291-123331,numTag 1.0000 40

ebv:124331-124371,numTag 1.0000 40 ebv:124471-124511,numTag 1.0000 40

ebv:125111-125151,numTag 1.0000 40 ebv:125151-125191,numTag 1.0000 40

ebv:125971-126011,numTag 1.0000 40 ebv:126111-126151,numTag 1.0000 40

ebv:126231-126271,numTag 1.0000 40 ebv:127491-127531,numTag 1.0000 40

ebv:129331-129371,numTag 1.0000 40 ebv:129751-129791,numTag 1.0000 40

ebv:130271-130311,numTag 1.0000 40 ebv:131031-131071,numTag 1.0000 40

ebv:131351-131391,numTag 1.0000 40 ebv:133231-133271,numTag 1.0000 40

ebv:133951-133991,numTag 1.0000 40 ebv:134531-134571,numTag 1.0000 40

ebv:137131-137171,numTag 1.0000 40 ebv:137891-137931,numTag 1.0000 40

ebv:137991-138031,numTag 1.0000 40 ebv:139491-139531,numTag 1.0000 40

ebv:139971-140011,numTag 1.0000 40 ebv:141211-141251,numTag 1.0000 40

ebv:141371-141411,numTag 1.0000 40 ebv:142331-142371,numTag 1.0000 40

ebv:142431-142471,numTag 1.0000 40 ebv:142951-142991,numTag 1.0000 40

ebv:143051-143091,numTag 1.0000 40 ebv:143471-143511,numTag 1.0000 40

ebv:144291-144331,numTag 1.0000 40 ebv:144691-144731,numTag 1.0000 40

ebv:145131-145171,numTag 1.0000 40 ebv:148171-148211,numTag 1.0000 40

ebv:150171-150211,numTag 1.0000 40 ebv:150491-150531,numTag 1.0000 40

ebv:150951-150991,numTag 1.0000 40 ebv:162091-162131,numTag 1.0000 40

ebv:162131-162171,numTag 1.0000 40 ebv:165071-165111,numTag 1.0000 40

ebv:166811-166851,numTag 1.0000 40 ebv:169611-169651,numTag 1.0000 40

ebv:171551-171591,numTag 1.0000 40 ebv:172131-172171,numTag 1.0000 40

ebv:172691-172731,numTag 1.0000 40 ebv:174631-174671,numTag 1.0000 40

ebv:176691-176731,numTag 1.0000 40 ebv:177471-177511,numTag 1.0000 40

ebv:177991-178031,numTag 1.0000 40 ebv:178131-178171,numTag 1.0000 40

ebv:179031-179071,numTag 1.0000 40 ebv:179291-179331,numTag 1.0000 40

ebv:2491-2531,numTags=25 1.0000 40 ebv:331-371,numTags=300 1.0000 40

ebv:3951-3991,numTags=35 1.0000 40 ebv:4671-4711,numTags=20 1.0000 40

ebv:48971-49011,numTags= 1.0000 40 ebv:49271-49311,numTags= 1.0000 40

ebv:5831-5871,numTags=84 1.0000 40 ebv:52831-52871,numTags= 1.0000 40

ebv:53711-53751,numTags= 1.0000 40 ebv:55271-55311,numTags= 1.0000 40

ebv:56291-56331,numTags= 1.0000 40 ebv:56591-56631,numTags= 1.0000 40

ebv:56751-56791,numTags= 1.0000 40 ebv:57211-57251,numTags= 1.0000 40

ebv:57271-57311,numTags= 1.0000 40 ebv:58191-58231,numTags= 1.0000 40

ebv:58511-58551,numTags= 1.0000 40 ebv:59411-59451,numTags= 1.0000 40

ebv:6371-6411,numTags=28 1.0000 40 ebv:70051-70091,numTags= 1.0000 40

ebv:70111-70151,numTags= 1.0000 40 ebv:71491-71531,numTags= 1.0000 40

ebv:72411-72451,numTags= 1.0000 40 ebv:76111-76151,numTags= 1.0000 40

ebv:76531-76571,numTags= 1.0000 40 ebv:78731-78771,numTags= 1.0000 40

ebv:79751-79791,numTags= 1.0000 40 ebv:8091-8131,numTags=86 1.0000 40

ebv:8551-8591,numTags=53 1.0000 40 ebv:8651-8691,numTags=48 1.0000 40

ebv:81911-81951,numTags= 1.0000 40 ebv:84451-84491,numTags= 1.0000 40

ebv:84811-84851,numTags= 1.0000 40 ebv:86171-86211,numTags= 1.0000 40

ebv:87251-87291,numTags= 1.0000 40 ebv:87411-87451,numTags= 1.0000 40

ebv:88571-88611,numTags= 1.0000 40 ebv:88751-88791,numTags= 1.0000 40

ebv:9151-9191,numTags=52 1.0000 40 ebv:9431-9471,numTags=20 1.0000 40

ebv:9571-9611,numTags=20 1.0000 40 ebv:90991-91031,numTags= 1.0000 40

ebv:91091-91131,numTags= 1.0000 40 ebv:91151-91191,numTags= 1.0000 40

ebv:91311-91351,numTags= 1.0000 40 ebv:92191-92231,numTags= 1.0000 40

ebv:92831-92871,numTags= 1.0000 40 ebv:95091-95131,numTags= 1.0000 40

ebv:95271-95311,numTags= 1.0000 40 ebv:95411-95451,numTags= 1.0000 40

ebv:95851-95891,numTags= 1.0000 40 ebv:98231-98271,numTags= 1.0000 40

ebv:98291-98331,numTags= 1.0000 40 ebv:98791-98831,numTags= 1.0000 40

********************************************************************************

********************************************************************************

COMMAND LINE SUMMARY

********************************************************************************

This information can also be useful in the event you wish to report a

problem with the MEME software.

command: meme sequences -sf meme_w:o_F-factor.txt -dna -mod zoops -nmotifs 3 -minw 6 -maxw 50 -time 7200 -maxsize 60000 -revcomp -oc . -nostatus

model: mod= zoops nmotifs= 3 evt= inf

object function= E-value of product of p-values

width: minw= 6 maxw= 40 minic= 0.00

width: wg= 11 ws= 1 endgaps= yes

nsites: minsites= 2 maxsites= 126 wnsites= 0.8

theta: prob= 1 spmap= uni spfuzz= 0.5

global: substring= yes branching= no wbranch= no

em: prior= dirichlet b= 0.01 maxiter= 50

distance= 1e-05

data: n= 5040 N= 126

strands: + -

sample: seed= 0 seqfrac= 1

Letter frequencies in dataset:

A 0.238 C 0.262 G 0.262 T 0.238

Background letter frequencies (from dataset with add-one prior applied):

A 0.239 C 0.261 G 0.261 T 0.239

********************************************************************************

********************************************************************************

MOTIF 1 width = 9 sites = 101 llr = 636 E-value = 8.4e-043

********************************************************************************

--------------------------------------------------------------------------------

Motif 1 Description

--------------------------------------------------------------------------------

Simplified A 5::6:15a1

pos.-specific C 2:1::54:1

probability G 2:81a:1:3

matrix T 1a13:4::5

bits 2.1 *

1.9 * *

1.7 * * *

1.4 * * *

Relative 1.2 * * *

Entropy 1.0 ** * *

(9.1 bits) 0.8 ** * *

0.6 *******

0.4 ********

0.2 *********

0.0 ---------

Multilevel ATGAGCAAT

consensus C T TC G

sequence

--------------------------------------------------------------------------------

--------------------------------------------------------------------------------

Motif 1 sites sorted by position p-value

--------------------------------------------------------------------------------

Sequence name Strand Start P-value Site

------------- ------ ----- --------- ---------

ebv:127491-127531,numTag + 14 3.31e-06 GGCTAAAAAT ATGAGCAAT TCCCCTGACC

ebv:130271-130311,numTag - 27 9.97e-06 AGAAA ATGAGCCAT GACTAGCGCA

ebv:176691-176731,numTag - 17 1.33e-05 ATGTGTTGAG ATGAGTCAT CCCGTGGAGA

ebv:150491-150531,numTag + 26 1.33e-05 GAAGCACTAA ATGAGTCAT TCCTAA

ebv:78731-78771,numTags= + 10 2.02e-05 GTTGACATC ATGAGCAAG GGCCCAGGGG

ebv:53711-53751,numTags= + 23 2.02e-05 GGGTGGAAAT ATGAGCAAG AATAAGGAC

ebv:72411-72451,numTags= + 14 2.66e-05 AGGCGGGCGA ATGTGTAAT CCCGGAACCG

ebv:49271-49311,numTags= - 16 2.66e-05 GGGTGCTTTG ATGAGTAAG AGGGTGCATT

ebv:179031-179071,numTag - 5 2.66e-05 ACTCCTACTG ATGAGTAAG TATT

ebv:57271-57311,numTags= - 30 3.41e-05 GG ATGTGCCAT GCCCCAGGCT

ebv:137991-138031,numTag - 3 3.41e-05 CCTGATTCAG ATGAGCCAG CA

ebv:81911-81951,numTags= + 19 4.11e-05 GCATCATTGC ATGTGTCAT GGCCACCCTC

ebv:58511-58551,numTags= - 13 4.11e-05 TGCCACCCAG ATGTGTCAT GGCTCACAAA

ebv:143471-143511,numTag - 25 4.11e-05 AGGAAAC ATGAGTCAG GACTGGATGC

ebv:137131-137171,numTag + 24 5.85e-05 TTCCGTCCCC GTGAGCAAT GGTGGGGA

ebv:110091-110131,numTag + 17 5.85e-05 CATTTACCTG GTGAGCAAT CAGACATGCG

ebv:76111-76151,numTags= - 13 6.98e-05 ATGATGGATG GTGAGTAAT AAATTCTTAA

ebv:95091-95131,numTags= + 21 8.46e-05 TGTTGTTCAC ATGTGTCAG GATGACGAGT

ebv:162091-162131,numTag + 7 8.46e-05 AGTATA ATGAGCAAC AGGGCCAAAC

ebv:145131-145171,numTag + 23 8.46e-05 TAAGACGGCC ATGAGCAAC ACGGCCAAC

ebv:101451-101491,numTag - 13 8.46e-05 AACCATGTTT ATGTGTCAG TCAAAGATCA

ebv:71491-71531,numTags= - 28 9.15e-05 GAAG ATGAGTAAC GGCGACTGGG

ebv:9571-9611,numTags=20 + 27 1.06e-04 AACTGCCCCA CTGTGCAAT GCAGC

ebv:9151-9191,numTags=52 - 7 1.06e-04 TCAAGAGGGC ATTAGCAAT AGTGTT

ebv:162131-162171,numTag + 25 1.06e-04 ACGAGAGACT CTGTGCAAT CAAACCC

ebv:150951-150991,numTag - 20 1.06e-04 CCCAGGGCAC ATTAGCAAT GTTCTAGGCA

ebv:148171-148211,numTag - 26 1.06e-04 CTGACT CTGTGCAAT ATCGCAATCG

ebv:143051-143091,numTag - 23 1.06e-04 TGGGCTGGT CTGAGCAAG GTGATCTCTG

ebv:103291-103331,numTag - 25 1.06e-04 ACTATGC ATGAGCCAC AGGCATTGCT

ebv:1051-1091,numTags=14 + 13 1.06e-04 TTCATGTCCA CTGAGCAAG ATCCTTCTGG

ebv:52831-52871,numTags= + 2 1.34e-04 T CTGTGTAAT ACTTTAAGGT

ebv:3951-3991,numTags=35 - 19 1.34e-04 TAGATGCAGG GTGAGCAAG ACGTCTTCCG

ebv:101151-101191,numTag - 16 1.34e-04 GCTGTACCTG ATGAGTCAC ACCCCACGGC

ebv:98231-98271,numTags= + 9 1.62e-04 ATCGAACG ATGAGTGAT TTCGCCCATG

ebv:8091-8131,numTags=86 + 23 1.62e-04 AGGCGCAAGT GTGTGTAAT TTGTCCTCC

ebv:174631-174671,numTag - 24 1.62e-04 GTTGCGTG ATGAGTAAA GTGTAACATT

ebv:57211-57251,numTags= - 8 1.91e-04 GCGACCCCCT GTGTGCCAT CAACCAC

ebv:139971-140011,numTag - 10 1.91e-04 TGCCGTTGTC CTGTGTCAT GCCCACCGT

ebv:133951-133991,numTag + 15 1.91e-04 CTCCATTAAC ATTAGTCAT ACCTGCCAGG

ebv:131031-131071,numTag + 27 1.91e-04 TGTCTCTGGC GTGTGCCAT CGCGT

ebv:131351-131391,numTag - 21 2.12e-04 CACTGCCACT ATGAGACAT ATTATGTCTT

ebv:122471-122511,numTag - 4 2.12e-04 GGCGGAGCAT ATGAGTCAA TTC

ebv:125971-126011,numTag + 12 2.30e-04 CCTCTTTCCC ATTAGCAAG AACCCCCTGC

ebv:5831-5871,numTags=84 + 20 2.61e-04 AATAAACCCA ATGTGCAAA TGTGGTTTGT

ebv:79751-79791,numTags= + 8 3.28e-04 AATTTCT TTGAGCAAG AGAGTTCCGA

ebv:144291-144331,numTag + 24 3.28e-04 TCACAGTCAG ATTTGTCAT CGAGCCCA

ebv:122791-122831,numTag - 17 3.28e-04 CCTCTGCCTT TTGAGCAAG TGCTACATCT

ebv:2491-2531,numTags=25 - 5 3.63e-04 AACACCTTCC ATGTGTCAA GGGT

ebv:179291-179331,numTag + 28 3.63e-04 CCTCAGGGCA GTGTGTCAG GAGC

ebv:177471-177511,numTag + 13 3.63e-04 TGCGCAAAGA ATGTGTCAA CAACAGGTGT

ebv:141371-141411,numTag - 12 3.63e-04 CCGCTCATCA ATGTGACAT TCATAATCTC

ebv:55271-55311,numTags= + 13 3.92e-04 CACACACGTA ATTTGCAAG CGGTGCTTCA

ebv:1271-1311,numTags=16 - 25 3.92e-04 AGCGACA ATCAGTAAT AACATGCAGA

ebv:144691-144731,numTag + 17 4.32e-04 TCACCTGCCC CTGAGTCAC TACCGGTTGG

ebv:92191-92231,numTags= + 25 4.78e-04 TGCGGTGTTG GTGAGTCAC ACTTTTG

ebv:58191-58231,numTags= + 10 4.78e-04 CGGGGGCAT CTGAGTGAT TGACCAGGGT

ebv:129751-129791,numTag - 19 4.78e-04 CCGCCCTGTC GTGAGTCAC GGCGGCGATT

ebv:88751-88791,numTags= + 26 5.17e-04 TGACGCTTGT CTTAGTCAT TATAGC

ebv:84811-84851,numTags= + 21 5.17e-04 CCCACGTGCA GTGAGTGAT GTAAGAGGTT

ebv:56591-56631,numTags= + 8 5.71e-04 ACGGCCT CTGAGACAT GTATGGGGGT

ebv:177991-178031,numTag + 2 5.71e-04 G GTTAGTCAT AGTAGCTTAG

ebv:171551-171591,numTag - 3 5.71e-04 GTTTGATGTG GTGTGCAAC CT

ebv:134531-134571,numTag - 14 5.71e-04 CACCTTTGTT ATGCGCAAT CCTCAGCAGC

ebv:88571-88611,numTags= + 10 6.25e-04 TTGCAAAGG CTGTGCCAC TGCTCTTCCT

ebv:76531-76571,numTags= - 8 6.25e-04 CGGCTACTGT GTGTGTAAC TGACTCG

ebv:331-371,numTags=300 + 30 6.25e-04 TCTCCTCAGT CTTTGCAAT TT

ebv:56751-56791,numTags= + 25 6.90e-04 CTTCCCAGCT CTGAGCGAG ATTTTCC

ebv:166811-166851,numTag + 18 6.90e-04 AGGCATGTTG CTGAGCGAG CTCGTCGATC

ebv:86171-86211,numTags= + 12 7.75e-04 GCAGTTTCTC GTGTGCAAA GTGGGCAAAA

ebv:59411-59451,numTags= + 29 7.75e-04 CATGGATATG ATTAGTGAT ATG

ebv:122931-122971,numTag + 27 7.75e-04 CAGTCCCATA ATCAGTCAG GACCA

ebv:98791-98831,numTags= + 9 8.42e-04 CCATTAAT TTTAGCAAT CGCACCTGCA

ebv:150171-150211,numTag + 28 9.16e-04 AGTAACCTGC ATGCGCAAG GGTC

ebv:142431-142471,numTag - 29 9.16e-04 GAG ATGAGCATG TGTTGAGCCT

ebv:1891-1931,numTags=67 - 13 9.16e-04 GCAGCAGGTT CTCAGCAAT CAGGGGCCCC

ebv:87411-87451,numTags= - 24 9.90e-04 GGTCACCG ATGAGACAA CGGCCGAGAT

ebv:8551-8591,numTags=53 - 10 9.90e-04 ATTTATGGCT ATGGGCAAC ACATAATCC

ebv:137891-137931,numTag - 6 9.90e-04 CAAATTGGTG ATGAGACAA GTAGC

ebv:126231-126271,numTag - 6 9.90e-04 AACGGGGAAT GTGTGACAT GCTAT

ebv:9431-9471,numTags=20 - 16 1.15e-03 CCTGTTCCAC ATGTGACAC GGGGGGGGAC

ebv:87251-87291,numTags= + 2 1.15e-03 G CTCAGTCAT CTGGAATACC

ebv:4671-4711,numTags=20 + 22 1.15e-03 CCTGGTCATC CTTTGCCAG CGAGCAGTAC

ebv:114471-114511,numTag + 23 1.15e-03 GCGATGACTC GTGTGCGAG CCGGTTTCG

ebv:95271-95311,numTags= + 11 1.23e-03 TTCATATTCC ATGAGAGAG ACCTCGCATA

ebv:95851-95891,numTags= + 6 1.32e-03 TATGG ATGTGCATG ACCGTGCGTC

ebv:8651-8691,numTags=48 - 27 1.44e-03 ACCCC TTGGGCAAT AAATACTAGT

ebv:125111-125151,numTag - 2 1.44e-03 GCTCGAAGCA TTGGGCAAT A

ebv:90991-91031,numTags= - 9 1.54e-03 TTTCTGGTGC ATTTGCGAG CAATCGGA

ebv:172691-172731,numTag - 3 1.54e-03 GCCCATCTGT ATGTGCTAT GA

ebv:98291-98331,numTags= + 25 1.76e-03 GGGGGCAGTG ATAAGTCAT GACAATT

ebv:125151-125191,numTag + 25 2.01e-03 GTAGCTTCTG ATGAATCAT TACCCAT

ebv:91311-91351,numTags= - 12 2.34e-03 GTCACGATTT CTTGGCAAT AACAGTATCC

ebv:133231-133271,numTag + 15 2.34e-03 TCTGTTGCAT CTTGGCAAT CTCATCGGTA

ebv:119091-119131,numTag - 20 2.34e-03 ATGGTCTCAG TTGAGCATT CACCTTTTAA

ebv:6371-6411,numTags=28 + 22 2.53e-03 ATGTGTCGAA ATGACCAAG CGTCCCCGCA

ebv:169611-169651,numTag + 9 2.53e-03 TACAGGCT GTGCGTCAG CGCGTGCAGG

ebv:84451-84491,numTags= + 9 2.67e-03 GGCCTGTG ATGTGTAGT AGAGTACCAG

ebv:115651-115691,numTag - 5 2.84e-03 TTTCCCAAAG TTCAGTCAG ATCC

ebv:124471-124511,numTag + 25 3.00e-03 AGGTTTGGGA GTGGGCCAA TATTTGC

ebv:142331-142371,numTag - 3 3.16e-03 GTTACAACCT TTGTGCATT AT

ebv:142951-142991,numTag - 20 3.79e-03 GGCTGCCAGC CTCAGCGAG GTTAAGCTGC

--------------------------------------------------------------------------------

--------------------------------------------------------------------------------

Motif 1 block diagrams

--------------------------------------------------------------------------------

SEQUENCE NAME POSITION P-VALUE MOTIF DIAGRAM

------------- ---------------- -------------

ebv:127491-127531,numTag 3.3e-06 13_[+1]_18

ebv:130271-130311,numTag 1e-05 26_[-1]_5

ebv:176691-176731,numTag 1.3e-05 16_[-1]_15

ebv:150491-150531,numTag 1.3e-05 25_[+1]_6

ebv:78731-78771,numTags= 2e-05 9_[+1]_22

ebv:53711-53751,numTags= 2e-05 22_[+1]_9

ebv:72411-72451,numTags= 2.7e-05 13_[+1]_18

ebv:49271-49311,numTags= 2.7e-05 15_[-1]_16

ebv:179031-179071,numTag 2.7e-05 4_[-1]_27

ebv:57271-57311,numTags= 3.4e-05 29_[-1]_2

ebv:137991-138031,numTag 3.4e-05 2_[-1]_29

ebv:81911-81951,numTags= 4.1e-05 18_[+1]_13

ebv:58511-58551,numTags= 4.1e-05 12_[-1]_19

ebv:143471-143511,numTag 4.1e-05 24_[-1]_7

ebv:137131-137171,numTag 5.9e-05 23_[+1]_8

ebv:110091-110131,numTag 5.9e-05 16_[+1]_15

ebv:76111-76151,numTags= 7e-05 12_[-1]_19

ebv:95091-95131,numTags= 8.5e-05 20_[+1]_11

ebv:162091-162131,numTag 8.5e-05 6_[+1]_25

ebv:145131-145171,numTag 8.5e-05 22_[+1]_9

ebv:101451-101491,numTag 8.5e-05 12_[-1]_19

ebv:71491-71531,numTags= 9.2e-05 27_[-1]_4

ebv:9571-9611,numTags=20 0.00011 26_[+1]_5

ebv:9151-9191,numTags=52 0.00011 6_[-1]_25

ebv:162131-162171,numTag 0.00011 24_[+1]_7

ebv:150951-150991,numTag 0.00011 19_[-1]_12

ebv:148171-148211,numTag 0.00011 25_[-1]_6

ebv:143051-143091,numTag 0.00011 22_[-1]_9

ebv:103291-103331,numTag 0.00011 24_[-1]_7

ebv:1051-1091,numTags=14 0.00011 12_[+1]_19

ebv:52831-52871,numTags= 0.00013 1_[+1]_30

ebv:3951-3991,numTags=35 0.00013 18_[-1]_13

ebv:101151-101191,numTag 0.00013 15_[-1]_16

ebv:98231-98271,numTags= 0.00016 8_[+1]_23

ebv:8091-8131,numTags=86 0.00016 22_[+1]_9

ebv:174631-174671,numTag 0.00016 23_[-1]_8

ebv:57211-57251,numTags= 0.00019 7_[-1]_24

ebv:139971-140011,numTag 0.00019 9_[-1]_22

ebv:133951-133991,numTag 0.00019 14_[+1]_17

ebv:131031-131071,numTag 0.00019 26_[+1]_5

ebv:131351-131391,numTag 0.00021 20_[-1]_11

ebv:122471-122511,numTag 0.00021 3_[-1]_28

ebv:125971-126011,numTag 0.00023 11_[+1]_20

ebv:5831-5871,numTags=84 0.00026 19_[+1]_12

ebv:79751-79791,numTags= 0.00033 7_[+1]_24

ebv:144291-144331,numTag 0.00033 23_[+1]_8

ebv:122791-122831,numTag 0.00033 16_[-1]_15

ebv:2491-2531,numTags=25 0.00036 4_[-1]_27

ebv:179291-179331,numTag 0.00036 27_[+1]_4

ebv:177471-177511,numTag 0.00036 12_[+1]_19

ebv:141371-141411,numTag 0.00036 11_[-1]_20

ebv:55271-55311,numTags= 0.00039 12_[+1]_19

ebv:1271-1311,numTags=16 0.00039 24_[-1]_7

ebv:144691-144731,numTag 0.00043 16_[+1]_15

ebv:92191-92231,numTags= 0.00048 24_[+1]_7

ebv:58191-58231,numTags= 0.00048 9_[+1]_22

ebv:129751-129791,numTag 0.00048 18_[-1]_13

ebv:88751-88791,numTags= 0.00052 25_[+1]_6

ebv:84811-84851,numTags= 0.00052 20_[+1]_11

ebv:56591-56631,numTags= 0.00057 7_[+1]_24

ebv:177991-178031,numTag 0.00057 1_[+1]_30

ebv:171551-171591,numTag 0.00057 2_[-1]_29

ebv:134531-134571,numTag 0.00057 13_[-1]_18

ebv:88571-88611,numTags= 0.00062 9_[+1]_22

ebv:76531-76571,numTags= 0.00062 7_[-1]_24

ebv:331-371,numTags=300 0.00062 29_[+1]_2

ebv:56751-56791,numTags= 0.00069 24_[+1]_7

ebv:166811-166851,numTag 0.00069 17_[+1]_14

ebv:86171-86211,numTags= 0.00077 11_[+1]_20

ebv:59411-59451,numTags= 0.00077 28_[+1]_3

ebv:122931-122971,numTag 0.00077 26_[+1]_5

ebv:98791-98831,numTags= 0.00084 8_[+1]_23

ebv:150171-150211,numTag 0.00092 27_[+1]_4

ebv:142431-142471,numTag 0.00092 28_[-1]_3

ebv:1891-1931,numTags=67 0.00092 12_[-1]_19

ebv:87411-87451,numTags= 0.00099 23_[-1]_8

ebv:8551-8591,numTags=53 0.00099 9_[-1]_22

ebv:137891-137931,numTag 0.00099 5_[-1]_26

ebv:126231-126271,numTag 0.00099 5_[-1]_26

ebv:9431-9471,numTags=20 0.0012 15_[-1]_16

ebv:87251-87291,numTags= 0.0012 1_[+1]_30

ebv:4671-4711,numTags=20 0.0012 21_[+1]_10

ebv:114471-114511,numTag 0.0012 22_[+1]_9

ebv:95271-95311,numTags= 0.0012 10_[+1]_21

ebv:95851-95891,numTags= 0.0013 5_[+1]_26

ebv:8651-8691,numTags=48 0.0014 26_[-1]_5

ebv:125111-125151,numTag 0.0014 1_[-1]_30

ebv:90991-91031,numTags= 0.0015 8_[-1]_23

ebv:172691-172731,numTag 0.0015 2_[-1]_29

ebv:98291-98331,numTags= 0.0018 24_[+1]_7

ebv:125151-125191,numTag 0.002 24_[+1]_7

ebv:91311-91351,numTags= 0.0023 11_[-1]_20

ebv:133231-133271,numTag 0.0023 14_[+1]_17

ebv:119091-119131,numTag 0.0023 19_[-1]_12

ebv:6371-6411,numTags=28 0.0025 21_[+1]_10

ebv:169611-169651,numTag 0.0025 8_[+1]_23

ebv:84451-84491,numTags= 0.0027 8_[+1]_23

ebv:115651-115691,numTag 0.0028 4_[-1]_27

ebv:124471-124511,numTag 0.003 24_[+1]_7

ebv:142331-142371,numTag 0.0032 2_[-1]_29

ebv:142951-142991,numTag 0.0038 19_[-1]_12

--------------------------------------------------------------------------------

--------------------------------------------------------------------------------

Motif 1 in BLOCKS format

--------------------------------------------------------------------------------

BL MOTIF 1 width=9 seqs=101

ebv:127491-127531,numTag ( 14) ATGAGCAAT 1

ebv:130271-130311,numTag ( 27) ATGAGCCAT 1

ebv:176691-176731,numTag ( 17) ATGAGTCAT 1

ebv:150491-150531,numTag ( 26) ATGAGTCAT 1

ebv:78731-78771,numTags= ( 10) ATGAGCAAG 1

ebv:53711-53751,numTags= ( 23) ATGAGCAAG 1

ebv:72411-72451,numTags= ( 14) ATGTGTAAT 1

ebv:49271-49311,numTags= ( 16) ATGAGTAAG 1

ebv:179031-179071,numTag ( 5) ATGAGTAAG 1

ebv:57271-57311,numTags= ( 30) ATGTGCCAT 1

ebv:137991-138031,numTag ( 3) ATGAGCCAG 1

ebv:81911-81951,numTags= ( 19) ATGTGTCAT 1

ebv:58511-58551,numTags= ( 13) ATGTGTCAT 1

ebv:143471-143511,numTag ( 25) ATGAGTCAG 1

ebv:137131-137171,numTag ( 24) GTGAGCAAT 1

ebv:110091-110131,numTag ( 17) GTGAGCAAT 1

ebv:76111-76151,numTags= ( 13) GTGAGTAAT 1

ebv:95091-95131,numTags= ( 21) ATGTGTCAG 1

ebv:162091-162131,numTag ( 7) ATGAGCAAC 1

ebv:145131-145171,numTag ( 23) ATGAGCAAC 1

ebv:101451-101491,numTag ( 13) ATGTGTCAG 1

ebv:71491-71531,numTags= ( 28) ATGAGTAAC 1

ebv:9571-9611,numTags=20 ( 27) CTGTGCAAT 1

ebv:9151-9191,numTags=52 ( 7) ATTAGCAAT 1

ebv:162131-162171,numTag ( 25) CTGTGCAAT 1

ebv:150951-150991,numTag ( 20) ATTAGCAAT 1

ebv:148171-148211,numTag ( 26) CTGTGCAAT 1

ebv:143051-143091,numTag ( 23) CTGAGCAAG 1

ebv:103291-103331,numTag ( 25) ATGAGCCAC 1

ebv:1051-1091,numTags=14 ( 13) CTGAGCAAG 1

ebv:52831-52871,numTags= ( 2) CTGTGTAAT 1

ebv:3951-3991,numTags=35 ( 19) GTGAGCAAG 1

ebv:101151-101191,numTag ( 16) ATGAGTCAC 1

ebv:98231-98271,numTags= ( 9) ATGAGTGAT 1

ebv:8091-8131,numTags=86 ( 23) GTGTGTAAT 1

ebv:174631-174671,numTag ( 24) ATGAGTAAA 1

ebv:57211-57251,numTags= ( 8) GTGTGCCAT 1

ebv:139971-140011,numTag ( 10) CTGTGTCAT 1

ebv:133951-133991,numTag ( 15) ATTAGTCAT 1

ebv:131031-131071,numTag ( 27) GTGTGCCAT 1

ebv:131351-131391,numTag ( 21) ATGAGACAT 1

ebv:122471-122511,numTag ( 4) ATGAGTCAA 1

ebv:125971-126011,numTag ( 12) ATTAGCAAG 1

ebv:5831-5871,numTags=84 ( 20) ATGTGCAAA 1

ebv:79751-79791,numTags= ( 8) TTGAGCAAG 1

ebv:144291-144331,numTag ( 24) ATTTGTCAT 1

ebv:122791-122831,numTag ( 17) TTGAGCAAG 1

ebv:2491-2531,numTags=25 ( 5) ATGTGTCAA 1

ebv:179291-179331,numTag ( 28) GTGTGTCAG 1

ebv:177471-177511,numTag ( 13) ATGTGTCAA 1

ebv:141371-141411,numTag ( 12) ATGTGACAT 1

ebv:55271-55311,numTags= ( 13) ATTTGCAAG 1

ebv:1271-1311,numTags=16 ( 25) ATCAGTAAT 1

ebv:144691-144731,numTag ( 17) CTGAGTCAC 1

ebv:92191-92231,numTags= ( 25) GTGAGTCAC 1

ebv:58191-58231,numTags= ( 10) CTGAGTGAT 1

ebv:129751-129791,numTag ( 19) GTGAGTCAC 1

ebv:88751-88791,numTags= ( 26) CTTAGTCAT 1

ebv:84811-84851,numTags= ( 21) GTGAGTGAT 1

ebv:56591-56631,numTags= ( 8) CTGAGACAT 1

ebv:177991-178031,numTag ( 2) GTTAGTCAT 1

ebv:171551-171591,numTag ( 3) GTGTGCAAC 1

ebv:134531-134571,numTag ( 14) ATGCGCAAT 1

ebv:88571-88611,numTags= ( 10) CTGTGCCAC 1

ebv:76531-76571,numTags= ( 8) GTGTGTAAC 1

ebv:331-371,numTags=300 ( 30) CTTTGCAAT 1

ebv:56751-56791,numTags= ( 25) CTGAGCGAG 1

ebv:166811-166851,numTag ( 18) CTGAGCGAG 1

ebv:86171-86211,numTags= ( 12) GTGTGCAAA 1

ebv:59411-59451,numTags= ( 29) ATTAGTGAT 1

ebv:122931-122971,numTag ( 27) ATCAGTCAG 1

ebv:98791-98831,numTags= ( 9) TTTAGCAAT 1

ebv:150171-150211,numTag ( 28) ATGCGCAAG 1

ebv:142431-142471,numTag ( 29) ATGAGCATG 1

ebv:1891-1931,numTags=67 ( 13) CTCAGCAAT 1

ebv:87411-87451,numTags= ( 24) ATGAGACAA 1

ebv:8551-8591,numTags=53 ( 10) ATGGGCAAC 1

ebv:137891-137931,numTag ( 6) ATGAGACAA 1

ebv:126231-126271,numTag ( 6) GTGTGACAT 1

ebv:9431-9471,numTags=20 ( 16) ATGTGACAC 1

ebv:87251-87291,numTags= ( 2) CTCAGTCAT 1

ebv:4671-4711,numTags=20 ( 22) CTTTGCCAG 1

ebv:114471-114511,numTag ( 23) GTGTGCGAG 1

ebv:95271-95311,numTags= ( 11) ATGAGAGAG 1

ebv:95851-95891,numTags= ( 6) ATGTGCATG 1

ebv:8651-8691,numTags=48 ( 27) TTGGGCAAT 1

ebv:125111-125151,numTag ( 2) TTGGGCAAT 1

ebv:90991-91031,numTags= ( 9) ATTTGCGAG 1

ebv:172691-172731,numTag ( 3) ATGTGCTAT 1

ebv:98291-98331,numTags= ( 25) ATAAGTCAT 1

ebv:125151-125191,numTag ( 25) ATGAATCAT 1

ebv:91311-91351,numTags= ( 12) CTTGGCAAT 1

ebv:133231-133271,numTag ( 15) CTTGGCAAT 1

ebv:119091-119131,numTag ( 20) TTGAGCATT 1

ebv:6371-6411,numTags=28 ( 22) ATGACCAAG 1

ebv:169611-169651,numTag ( 9) GTGCGTCAG 1

ebv:84451-84491,numTags= ( 9) ATGTGTAGT 1

ebv:115651-115691,numTag ( 5) TTCAGTCAG 1

ebv:124471-124511,numTag ( 25) GTGGGCCAA 1

ebv:142331-142371,numTag ( 3) TTGTGCATT 1

ebv:142951-142991,numTag ( 20) CTCAGCGAG 1

//

--------------------------------------------------------------------------------

--------------------------------------------------------------------------------

Motif 1 position-specific scoring matrix

--------------------------------------------------------------------------------

log-odds matrix: alength= 4 w= 9 n= 4032 bayes= 5.79365 E= 8.4e-043

114 -33 -48 -159

-1330 -1330 -1330 207

-459 -214 158 -68

124 -314 -214 54

-459 -472 191 -1330

-159 98 -1330 77

99 67 -140 -459

199 -1330 -472 -259

-142 -102 18 102

--------------------------------------------------------------------------------

--------------------------------------------------------------------------------

Motif 1 position-specific probability matrix

--------------------------------------------------------------------------------

letter-probability matrix: alength= 4 w= 9 nsites= 101 E= 8.4e-043

0.524752 0.207921 0.188119 0.079208

0.000000 0.000000 0.000000 1.000000

0.009901 0.059406 0.782178 0.148515

0.564356 0.029703 0.059406 0.346535

0.009901 0.009901 0.980198 0.000000

0.079208 0.514851 0.000000 0.405941

0.475248 0.415842 0.099010 0.009901

0.950495 0.000000 0.009901 0.039604

0.089109 0.128713 0.297030 0.485149

--------------------------------------------------------------------------------

--------------------------------------------------------------------------------

Motif 1 regular expression

--------------------------------------------------------------------------------

[AC]TG[AT]G[CT][AC]A[TG]

--------------------------------------------------------------------------------

********************************************************************************

MEME - Motif discovery tool

********************************************************************************

MEME version 4.4.0 (Release date: Tue Apr 27 10:09:30 EST 2010)

For further information on how to interpret these results or to get

a copy of the MEME software please access http://meme.nbcr.net.

This file may be used as input to the MAST algorithm for searching

sequence databases for matches to groups of motifs. MAST is available

for interactive use and downloading at http://meme.nbcr.net.

********************************************************************************

********************************************************************************

REFERENCE

********************************************************************************

If you use this program in your research, please cite:

Timothy L. Bailey and Charles Elkan,

"Fitting a mixture model by expectation maximization to discover

motifs in biopolymers", Proceedings of the Second International

Conference on Intelligent Systems for Molecular Biology, pp. 28-36,

AAAI Press, Menlo Park, California, 1994.

********************************************************************************

********************************************************************************

TRAINING SET

********************************************************************************

DATAFILE= **meme-meth_w:o_F-factor.txt**

ALPHABET= ACGT

Sequence name Weight Length Sequence name Weight Length

------------- ------ ------ ------------- ------ ------

ebv:1111-1151,numTags=64 1.0000 40 ebv:1531-1571,numTags=44 1.0000 40

ebv:1711-1751,numTags=53 1.0000 40 ebv:1871-1911,numTags=39 1.0000 40

ebv:1991-2031,numTags=20 1.0000 40 ebv:11091-11131,numTags= 1.0000 40

ebv:11211-11251,numTags= 1.0000 40 ebv:100471-100511,numTag 1.0000 40

ebv:100571-100611,numTag 1.0000 40 ebv:101151-101191,numTag 1.0000 40

ebv:101451-101491,numTag 1.0000 40 ebv:102531-102571,numTag 1.0000 40

ebv:102651-102691,numTag 1.0000 40 ebv:103271-103311,numTag 1.0000 40

ebv:105471-105511,numTag 1.0000 40 ebv:106351-106391,numTag 1.0000 40

ebv:106891-106931,numTag 1.0000 40 ebv:109551-109591,numTag 1.0000 40

ebv:110071-110111,numTag 1.0000 40 ebv:110811-110851,numTag 1.0000 40

ebv:110891-110931,numTag 1.0000 40 ebv:112711-112751,numTag 1.0000 40

ebv:112831-112871,numTag 1.0000 40 ebv:113951-113991,numTag 1.0000 40

ebv:114471-114511,numTag 1.0000 40 ebv:114591-114631,numTag 1.0000 40

ebv:115331-115371,numTag 1.0000 40 ebv:116411-116451,numTag 1.0000 40

ebv:117871-117911,numTag 1.0000 40 ebv:118791-118831,numTag 1.0000 40

ebv:118931-118971,numTag 1.0000 40 ebv:119091-119131,numTag 1.0000 40

ebv:119771-119811,numTag 1.0000 40 ebv:120171-120211,numTag 1.0000 40

ebv:120911-120951,numTag 1.0000 40 ebv:121991-122031,numTag 1.0000 40

ebv:122471-122511,numTag 1.0000 40 ebv:122791-122831,numTag 1.0000 40

ebv:123291-123331,numTag 1.0000 40 ebv:123931-123971,numTag 1.0000 40

ebv:124111-124151,numTag 1.0000 40 ebv:124471-124511,numTag 1.0000 40

ebv:124971-125011,numTag 1.0000 40 ebv:125091-125131,numTag 1.0000 40

ebv:125671-125711,numTag 1.0000 40 ebv:125851-125891,numTag 1.0000 40

ebv:125971-126011,numTag 1.0000 40 ebv:126091-126131,numTag 1.0000 40

ebv:126231-126271,numTag 1.0000 40 ebv:126771-126811,numTag 1.0000 40

ebv:127491-127531,numTag 1.0000 40 ebv:128451-128491,numTag 1.0000 40

ebv:128591-128631,numTag 1.0000 40 ebv:129431-129471,numTag 1.0000 40

ebv:129571-129611,numTag 1.0000 40 ebv:129731-129771,numTag 1.0000 40

ebv:130291-130331,numTag 1.0000 40 ebv:131151-131191,numTag 1.0000 40

ebv:131391-131431,numTag 1.0000 40 ebv:133951-133991,numTag 1.0000 40

ebv:134451-134491,numTag 1.0000 40 ebv:134871-134911,numTag 1.0000 40

ebv:136711-136751,numTag 1.0000 40 ebv:137131-137171,numTag 1.0000 40

ebv:137911-137951,numTag 1.0000 40 ebv:137971-138011,numTag 1.0000 40

ebv:138631-138671,numTag 1.0000 40 ebv:138751-138791,numTag 1.0000 40

ebv:139451-139491,numTag 1.0000 40 ebv:139951-139991,numTag 1.0000 40

ebv:140391-140431,numTag 1.0000 40 ebv:141371-141411,numTag 1.0000 40

ebv:142331-142371,numTag 1.0000 40 ebv:142951-142991,numTag 1.0000 40

ebv:143471-143511,numTag 1.0000 40 ebv:144291-144331,numTag 1.0000 40

ebv:144591-144631,numTag 1.0000 40 ebv:145211-145251,numTag 1.0000 40

ebv:146211-146251,numTag 1.0000 40 ebv:147151-147191,numTag 1.0000 40

ebv:147591-147631,numTag 1.0000 40 ebv:148171-148211,numTag 1.0000 40

ebv:148971-149011,numTag 1.0000 40 ebv:150171-150211,numTag 1.0000 40

ebv:150491-150531,numTag 1.0000 40 ebv:150951-150991,numTag 1.0000 40

ebv:162131-162171,numTag 1.0000 40 ebv:163051-163091,numTag 1.0000 40

ebv:163311-163351,numTag 1.0000 40 ebv:164451-164491,numTag 1.0000 40

ebv:165011-165051,numTag 1.0000 40 ebv:165911-165951,numTag 1.0000 40

ebv:166431-166471,numTag 1.0000 40 ebv:166811-166851,numTag 1.0000 40

ebv:168311-168351,numTag 1.0000 40 ebv:168391-168431,numTag 1.0000 40

ebv:169131-169171,numTag 1.0000 40 ebv:169511-169551,numTag 1.0000 40

ebv:169711-169751,numTag 1.0000 40 ebv:169831-169871,numTag 1.0000 40

ebv:170651-170691,numTag 1.0000 40 ebv:171431-171471,numTag 1.0000 40

ebv:171491-171531,numTag 1.0000 40 ebv:172111-172151,numTag 1.0000 40

ebv:172731-172771,numTag 1.0000 40 ebv:173351-173391,numTag 1.0000 40

ebv:173491-173531,numTag 1.0000 40 ebv:173691-173731,numTag 1.0000 40

ebv:174771-174811,numTag 1.0000 40 ebv:175331-175371,numTag 1.0000 40

ebv:175671-175711,numTag 1.0000 40 ebv:176691-176731,numTag 1.0000 40

ebv:177391-177431,numTag 1.0000 40 ebv:177991-178031,numTag 1.0000 40

ebv:178131-178171,numTag 1.0000 40 ebv:179031-179071,numTag 1.0000 40

ebv:179851-179891,numTag 1.0000 40 ebv:2511-2551,numTags=56 1.0000 40

ebv:3971-4011,numTags=11 1.0000 40 ebv:4051-4091,numTags=10 1.0000 40

ebv:4091-4131,numTags=11 1.0000 40 ebv:4371-4411,numTags=67 1.0000 40

ebv:4671-4711,numTags=56 1.0000 40 ebv:48111-48151,numTags= 1.0000 40

ebv:49271-49311,numTags= 1.0000 40 ebv:5831-5871,numTags=14 1.0000 40

ebv:52831-52871,numTags= 1.0000 40 ebv:53611-53651,numTags= 1.0000 40

ebv:53711-53751,numTags= 1.0000 40 ebv:55271-55311,numTags= 1.0000 40

ebv:56751-56791,numTags= 1.0000 40 ebv:57211-57251,numTags= 1.0000 40

ebv:57271-57311,numTags= 1.0000 40 ebv:57351-57391,numTags= 1.0000 40

ebv:58171-58211,numTags= 1.0000 40 ebv:58511-58551,numTags= 1.0000 40

ebv:59431-59471,numTags= 1.0000 40 ebv:6931-6971,numTags=10 1.0000 40

ebv:6971-7011,numTags=10 1.0000 40 ebv:61451-61491,numTags= 1.0000 40

ebv:62431-62471,numTags= 1.0000 40 ebv:64091-64131,numTags= 1.0000 40

ebv:64131-64171,numTags= 1.0000 40 ebv:64591-64631,numTags= 1.0000 40

ebv:65071-65111,numTags= 1.0000 40 ebv:66611-66651,numTags= 1.0000 40

ebv:67491-67531,numTags= 1.0000 40 ebv:67971-68011,numTags= 1.0000 40

ebv:68271-68311,numTags= 1.0000 40 ebv:69011-69051,numTags= 1.0000 40

ebv:711-751,numTags=6439 1.0000 40 ebv:70051-70091,numTags= 1.0000 40

ebv:70631-70671,numTags= 1.0000 40 ebv:71071-71111,numTags= 1.0000 40

ebv:71131-71171,numTags= 1.0000 40 ebv:71491-71531,numTags= 1.0000 40

ebv:72071-72111,numTags= 1.0000 40 ebv:72411-72451,numTags= 1.0000 40

ebv:74971-75011,numTags= 1.0000 40 ebv:75351-75391,numTags= 1.0000 40

ebv:76111-76151,numTags= 1.0000 40 ebv:76531-76571,numTags= 1.0000 40

ebv:77911-77951,numTags= 1.0000 40 ebv:78731-78771,numTags= 1.0000 40

ebv:78851-78891,numTags= 1.0000 40 ebv:79611-79651,numTags= 1.0000 40

ebv:8111-8151,numTags=29 1.0000 40 ebv:81911-81951,numTags= 1.0000 40

ebv:83851-83891,numTags= 1.0000 40 ebv:84631-84671,numTags= 1.0000 40

ebv:85431-85471,numTags= 1.0000 40 ebv:85891-85931,numTags= 1.0000 40

ebv:86031-86071,numTags= 1.0000 40 ebv:86171-86211,numTags= 1.0000 40

ebv:86531-86571,numTags= 1.0000 40 ebv:87131-87171,numTags= 1.0000 40

ebv:87251-87291,numTags= 1.0000 40 ebv:87411-87451,numTags= 1.0000 40

ebv:87671-87711,numTags= 1.0000 40 ebv:971-1011,numTags=324 1.0000 40

ebv:9131-9171,numTags=92 1.0000 40 ebv:9271-9311,numTags=57 1.0000 40

ebv:9431-9471,numTags=30 1.0000 40 ebv:9591-9631,numTags=37 1.0000 40

ebv:90071-90111,numTags= 1.0000 40 ebv:90991-91031,numTags= 1.0000 40

ebv:92191-92231,numTags= 1.0000 40 ebv:92671-92711,numTags= 1.0000 40

ebv:92831-92871,numTags= 1.0000 40 ebv:93211-93251,numTags= 1.0000 40

ebv:93951-93991,numTags= 1.0000 40 ebv:94271-94311,numTags= 1.0000 40

ebv:95871-95911,numTags= 1.0000 40 ebv:95951-95991,numTags= 1.0000 40

ebv:98031-98071,numTags= 1.0000 40 ebv:98231-98271,numTags= 1.0000 40

ebv:98311-98351,numTags= 1.0000 40 ebv:98391-98431,numTags= 1.0000 40

ebv:98791-98831,numTags= 1.0000 40 ebv:98931-98971,numTags= 1.0000 40

********************************************************************************

********************************************************************************

COMMAND LINE SUMMARY

********************************************************************************

This information can also be useful in the event you wish to report a

problem with the MEME software.

command: meme sequences -sf meme-meth_w:o_F-factor.txt -dna -mod zoops -nmotifs 3 -minw 6 -maxw 50 -time 7200 -maxsize 60000 -revcomp -oc . -nostatus

model: mod= zoops nmotifs= 3 evt= inf

object function= E-value of product of p-values

width: minw= 6 maxw= 40 minic= 0.00

width: wg= 11 ws= 1 endgaps= yes

nsites: minsites= 2 maxsites= 200 wnsites= 0.8

theta: prob= 1 spmap= uni spfuzz= 0.5

global: substring= yes branching= no wbranch= no

em: prior= dirichlet b= 0.01 maxiter= 50

distance= 1e-05

data: n= 8000 N= 200

strands: + -

sample: seed= 0 seqfrac= 1

Letter frequencies in dataset:

A 0.222 C 0.278 G 0.278 T 0.222

Background letter frequencies (from dataset with add-one prior applied):

A 0.222 C 0.278 G 0.278 T 0.222

********************************************************************************

********************************************************************************

MOTIF 1 width = 9 sites = 167 llr = 937 E-value = 1.7e-039

********************************************************************************

--------------------------------------------------------------------------------

Motif 1 Description

--------------------------------------------------------------------------------

Simplified A 4::6::381

pos.-specific C 221::72:1

probability G 3:81a:514

matrix T 1813:3:14

bits 2.2

2.0 *

1.7 *

1.5 *

Relative 1.3 * * *

Entropy 1.1 * ** *

(8.1 bits) 0.9 ***** *

0.7 ***** *

0.4 *******

0.2 *********

0.0 ---------

Multilevel ATGAGCGAT

consensus G T TA G

sequence C

--------------------------------------------------------------------------------

--------------------------------------------------------------------------------

Motif 1 sites sorted by position p-value

--------------------------------------------------------------------------------

Sequence name Strand Start P-value Site

------------- ------ ----- --------- ---------

ebv:106351-106391,numTag + 20 3.22e-06 AACTAAGCTT ATGAGCGAT TTTATCACAG

ebv:84631-84671,numTags= + 14 9.83e-06 CAGGGTGCTC ATGAGCGAG GGCCAGATGC

ebv:177391-177431,numTag + 9 9.83e-06 GTGGAAAG ATGAGCGAG GACAGGTGTG

ebv:127491-127531,numTag + 14 9.83e-06 GGCTAAAAAT ATGAGCAAT TCCCCTGACC

ebv:83851-83891,numTags= + 16 1.71e-05 CTGCACAGGG GTGAGCGAT GCAATGTGAC

ebv:165011-165051,numTag + 4 1.71e-05 GAT GTGAGCGAT CTTGGCAATC

ebv:130291-130331,numTag - 7 2.03e-05 TTGCGAGAAA ATGAGCCAT GACTAG

ebv:98231-98271,numTags= + 9 2.61e-05 ATCGAACG ATGAGTGAT TTCGCCCATG

ebv:78731-78771,numTags= + 10 2.61e-05 GTTGACATC ATGAGCAAG GGCCCAGGGG

ebv:53711-53751,numTags= + 23 2.61e-05 GGGTGGAAAT ATGAGCAAG AATAAGGAC

ebv:79611-79651,numTags= + 7 3.67e-05 TGGTGG ATGTGCGAG CCATAAAGCA

ebv:175331-175371,numTag - 19 3.67e-05 ACAGGAGGAG CTGAGCGAT GAACCTGGCC

ebv:126771-126811,numTag - 25 3.67e-05 CTGGGTG CTGAGCGAT GCCTCTACGC

ebv:86531-86571,numTags= + 16 4.50e-05 CACGAGGGGC GTGAGCGAG CGCAGGGCCC

ebv:66611-66651,numTags= - 6 4.50e-05 GACGGCCATC GTGAGCGAG TACGT

ebv:137131-137171,numTag + 24 4.50e-05 TTCCGTCCCC GTGAGCAAT GGTGGGGA

ebv:137971-138011,numTag - 23 5.31e-05 CTGATTCAG ATGAGCCAG CATCTTTGCA

ebv:57271-57311,numTags= - 30 6.41e-05 GG ATGTGCCAT GCCCCAGGCT

ebv:87671-87711,numTags= - 15 8.22e-05 TGATAGTGGG CTGAGCGAG GCGCTCGAGG

ebv:85431-85471,numTags= - 8 8.22e-05 ACAGAACAGC CTGAGCGAG CAGCTCT

ebv:58511-58551,numTags= + 6 8.22e-05 ACTTT GTGAGCCAT GACACATCTG

ebv:56751-56791,numTags= + 25 8.22e-05 CTTCCCAGCT CTGAGCGAG ATTTTCC

ebv:166811-166851,numTag + 18 8.22e-05 AGGCATGTTG CTGAGCGAG CTCGTCGATC

ebv:119771-119811,numTag - 25 8.22e-05 GAACACG CTGAGCGAG AAGACCAGCG

ebv:176691-176731,numTag - 17 1.16e-04 ATGTGTTGAG ATGAGTCAT CCCGTGGAGA

ebv:150491-150531,numTag + 26 1.16e-04 GAAGCACTAA ATGAGTCAT TCCTAA

ebv:114471-114511,numTag + 23 1.16e-04 GCGATGACTC GTGTGCGAG CCGGTTTCG

ebv:9131-9171,numTags=92 - 27 1.35e-04 AGGGC ATTAGCAAT AGTGTTTATA

ebv:49271-49311,numTags= - 16 1.35e-04 GGGTGCTTTG ATGAGTAAG AGGGTGCATT

ebv:179031-179071,numTag - 5 1.35e-04 ACTCCTACTG ATGAGTAAG TATT

ebv:150951-150991,numTag - 20 1.35e-04 CCCAGGGCAC ATTAGCAAT GTTCTAGGCA

ebv:72411-72451,numTags= + 14 1.59e-04 AGGCGGGCGA ATGTGTAAT CCCGGAACCG

ebv:67971-68011,numTags= + 8 1.59e-04 ACCCTGC ATTTGCGAT GTACTTTTCT

ebv:58171-58211,numTags= + 30 1.59e-04 ACGGGGGCAT CTGAGTGAT TG

ebv:9591-9631,numTags=37 + 7 1.88e-04 GCCCCA CTGTGCAAT GCAGCTTTTA

ebv:76111-76151,numTags= - 13 1.88e-04 ATGATGGATG GTGAGTAAT AAATTCTTAA

ebv:57211-57251,numTags= - 8 1.88e-04 GCGACCCCCT GTGTGCCAT CAACCAC

ebv:179851-179891,numTag + 31 1.88e-04 AGGGTGTGGG CTGTGCGAG T

ebv:162131-162171,numTag + 25 1.88e-04 ACGAGAGACT CTGTGCAAT CAAACCC

ebv:148171-148211,numTag - 26 1.88e-04 CTGACT CTGTGCAAT ATCGCAATCG

ebv:77911-77951,numTags= - 30 2.23e-04 TA ACGAGCGAG AGGGAGGGTC

ebv:68271-68311,numTags= - 17 2.23e-04 CCTTAGTGAT ATGAGCGGT ATCCCCTGGC

ebv:2511-2551,numTags=56 - 20 2.23e-04 GCTCCAAACT ATGAGCGGT TCGTGTGTCC

ebv:143471-143511,numTag - 25 2.23e-04 AGGAAAC ATGAGTCAG GACTGGATGC

ebv:131151-131191,numTag + 20 2.23e-04 TATGCAGGGA ATGAGCGGT CCGTGAGCCG

ebv:115331-115371,numTag + 11 2.23e-04 GATCAGATAA ACGAGCAAT TTGACCAGAT

ebv:81911-81951,numTags= + 19 2.46e-04 GCATCATTGC ATGTGTCAT GGCCACCCTC

ebv:59431-59471,numTags= + 9 2.46e-04 TGGATATG ATTAGTGAT ATGTCTCAGC

ebv:129571-129611,numTag + 22 2.46e-04 CACCTCCTCG CTGAGCCAG GGTAGCCTGA

ebv:125971-126011,numTag + 12 2.46e-04 CCTCTTTCCC ATTAGCAAG AACCCCCTGC

ebv:90991-91031,numTags= - 9 2.95e-04 TTTCTGGTGC ATTTGCGAG CAATCGGA

ebv:134451-134491,numTag + 14 2.95e-04 GTATTTGCCC ATGGGCGAG CGGTGCCACT

ebv:100471-100511,numTag - 6 2.95e-04 GGGGCCCATG ATGGGCGAG GCGTT

ebv:92671-92711,numTags= + 10 3.39e-04 CATCGACAC ACGAGCCAT AGACCAGTTT

ebv:67491-67531,numTags= + 21 3.39e-04 CTTCCCTGGG ATGAGCGTT TGGGAGAGCT

ebv:122791-122831,numTag - 17 3.39e-04 CCTCTGCCTT TTGAGCAAG TGCTACATCT

ebv:114591-114631,numTag + 25 3.39e-04 GTGCTCTCCT ATGAGCGTT ATGTGGA

ebv:98391-98431,numTags= + 26 3.92e-04 TCACCCGACA ATGAGCGGG GAGATA

ebv:8111-8151,numTags=29 + 3 3.92e-04 GT GTGTGTAAT TTGTCCTCCA

ebv:71071-71111,numTags= + 13 3.92e-04 CAACTGCCGA GTTTGCGAT CTGGGCAGGG

ebv:168391-168431,numTag - 24 3.92e-04 AACCTTCC ATGAGCGGG CAGACTCCTT

ebv:141371-141411,numTag + 23 3.92e-04 TGTCACATTG ATGAGCGGG ATAATCATC

ebv:118931-118971,numTag - 16 3.92e-04 CAGGGCGGTG GTGGGCGAT TTGGGTCGTG

ebv:150171-150211,numTag - 7 4.41e-04 AGGTTACTAC ACGTGCAAT GTGACA

ebv:101451-101491,numTag - 13 4.41e-04 AACCATGTTT ATGTGTCAG TCAAAGATCA

ebv:1531-1571,numTags=44 - 8 4.41e-04 CATGTAAGTC ATGTGTCAG AACGGTG

ebv:93211-93251,numTags= + 2 5.09e-04 T GTCAGCGAT GCCCTGGGCA

ebv:61451-61491,numTags= + 17 5.09e-04 TTGCCACCTG GTGAGCGGT GTGCGGGCTG

ebv:55271-55311,numTags= + 13 5.09e-04 CACACACGTA ATTTGCAAG CGGTGCTTCA

ebv:5831-5871,numTags=14 + 20 5.09e-04 AATAAACCCA ATGTGCAAA TGTGGTTTGT

ebv:172731-172771,numTag + 10 5.09e-04 TGGTCAGCA GTGAGCGGT AAAACAGCTG

ebv:147591-147631,numTag + 7 5.09e-04 GAGACC GTGAGCGGT TACCGGGGCG

ebv:53611-53651,numTags= - 18 5.76e-04 CAGGGGCTTA GTGTGTCAT GGTGAGGCAG

ebv:52831-52871,numTags= + 2 5.76e-04 T CTGTGTAAT ACTTTAAGGT

ebv:71491-71531,numTags= - 28 6.48e-04 GAAG ATGAGTAAC GGCGACTGGG

ebv:69011-69051,numTags= + 18 6.48e-04 CTGATAGCAT GTTTGCGAG GTTTTGGATG

ebv:174771-174811,numTag - 6 6.48e-04 GCAGACTCTG GTTTGCGAG GCTGG

ebv:170651-170691,numTag - 13 6.48e-04 CTGGCGCTGT GTGGGCGAG CTGATGGTTC

ebv:133951-133991,numTag + 15 6.48e-04 CTCCATTAAC ATTAGTCAT ACCTGCCAGG

ebv:110071-110111,numTag + 19 6.48e-04 GTTGGAAAAC ATTAGCGAC ATTTACCTGG

ebv:48111-48151,numTags= + 6 7.36e-04 GATTA ACGTGCAAG ACGCTAAACT

ebv:166431-166471,numTag - 14 7.36e-04 GGGGTGGTGG ATGTGCGGG GGCCTCAGCC

ebv:126231-126271,numTag - 8 7.36e-04 ATAACGGGGA ATGTGTGAC ATGCTAT

ebv:125671-125711,numTag - 29 7.36e-04 CTG GTGAGCCAC GTAGCCTATT

ebv:1711-1751,numTags=53 + 15 7.36e-04 GTGTAACTTG ACGTGCAAG GATGGAAGAG

ebv:93951-93991,numTags= + 13 8.29e-04 ACAGTGGCAC TTGAGCGAC CAGTTTACCC

ebv:171491-171531,numTag + 25 8.29e-04 GTCGTCCCTA GTCAGCGAG GCGCATG

ebv:147151-147191,numTag - 28 8.29e-04 ACCC CCGTGCGAT GAGTTTATTT

ebv:139951-139991,numTag - 30 8.29e-04 TC CTGTGTCAT GCCCACCGTC

ebv:128591-128631,numTag - 26 8.29e-04 TCCGGG GTCAGCGAG GCCCTCTTTG

ebv:102531-102571,numTag + 2 8.29e-04 T GCGAGCAAG GGAATGCGTT

ebv:98791-98831,numTags= + 9 9.39e-04 CCATTAAT TTTAGCAAT CGCACCTGCA

ebv:64591-64631,numTags= + 10 9.39e-04 GAAGCACCG GCGTGCGAG GAGCAGCATG

ebv:110811-110851,numTag - 21 9.39e-04 GCCAATGTCT GCGTGCGAG CCGGGCTTGC

ebv:101151-101191,numTag - 16 9.39e-04 GCTGTACCTG ATGAGTCAC ACCCCACGGC

ebv:11091-11131,numTags= - 10 9.39e-04 GGCCACTATA CTTTGCGAG CCCTGCGTC

ebv:98031-98071,numTags= + 9 1.04e-03 CCGTCTAA ACGAGCGAA GATCGAGGCC

ebv:86171-86211,numTags= + 12 1.04e-03 GCAGTTTCTC GTGTGCAAA GTGGGCAAAA

ebv:6971-7011,numTags=10 - 23 1.04e-03 AGCACCGCA CTGAGCGTT GGCGGTGTGT

ebv:124111-124151,numTag + 7 1.04e-03 TTTATA CTGAGCGTT TAGGTTTTGT

ebv:123931-123971,numTag - 9 1.04e-03 ACTTATGAAG ATGAGTAGT TCTAAGCG

ebv:122471-122511,numTag - 4 1.04e-03 GGCGGAGCAT ATGAGTCAA TTC

ebv:118791-118831,numTag - 13 1.04e-03 GAACTCAACT TTTTGCGAT GGGAGCGGGA

ebv:9271-9311,numTags=57 - 28 1.15e-03 CTTT ATGTGTAAC TCTTGGCTGA

ebv:169511-169551,numTag + 28 1.15e-03 CGTCACAGCA GCGAGCCAG GGCC

ebv:144291-144331,numTag + 24 1.15e-03 TCACAGTCAG ATTTGTCAT CGAGCCCA

ebv:142951-142991,numTag - 20 1.15e-03 GGCTGCCAGC CTCAGCGAG GTTAAGCTGC

ebv:72071-72111,numTags= + 27 1.29e-03 TGGACCCGGG CCGTGCGAG CAAAG

ebv:177991-178031,numTag + 2 1.29e-03 G GTTAGTCAT AGTAGCTTAG

ebv:74971-75011,numTags= - 26 1.43e-03 ACCCTC ATGAGCGGC CACGCTTACC

ebv:4371-4411,numTags=67 + 14 1.43e-03 AGGGCATGAC ATCAGCGAC GGGGGCCTGG

ebv:128451-128491,numTag + 6 1.43e-03 CTTTT GTGAGTAAA TAGAGATGAT

ebv:100571-100611,numTag - 15 1.43e-03 GGGTTGTTCT TCGTGCGAT ATCGGCTGTG

ebv:1111-1151,numTags=64 - 22 1.43e-03 AAATACTGCC ACCAGCGAT TAGCGCGGAG

ebv:175671-175711,numTag + 21 1.56e-03 GCAGGAACAC CTGAGCGTG GTGAAGCCTC

ebv:173351-173391,numTag + 10 1.56e-03 GCAAGAAGG TTGGGCGAG AAGGAGGCCG

ebv:140391-140431,numTag - 22 1.56e-03 TGTGGGAGCG GTTGGCGAT GAGCACGTTG

ebv:125091-125131,numTag - 22 1.56e-03 GCTCGAAGCA TTGGGCAAT ATAAATTACT

ebv:92191-92231,numTags= + 25 1.73e-03 TGCGGTGTTG GTGAGTCAC ACTTTTG

ebv:145211-145251,numTag - 31 1.73e-03 G ACGTGCGAA GAAAATTGGC

ebv:136711-136751,numTag - 20 1.73e-03 TTTTCTCAGT ACGTGCGAA GCCGTCTCTC

ebv:78851-78891,numTags= - 26 1.91e-03 ACGTTT ATTGGCAAG ATTCACAGGC

ebv:64131-64171,numTags= + 24 1.91e-03 CTGCAGAAAA TTCAGCGAG ATGGTCTC

ebv:4671-4711,numTags=56 + 22 1.91e-03 CCTGGTCATC CTTTGCCAG CGAGCAGTAC

ebv:163311-163351,numTag + 2 1.91e-03 T CTTTGCCAG CCTTCATACT

ebv:94271-94311,numTags= - 7 2.10e-03 CCCGGACCAC ATCAGCAAC CTGGCT

ebv:76531-76571,numTags= - 8 2.10e-03 CGGCTACTGT GTGTGTAAC TGACTCG

ebv:173491-173531,numTag + 16 2.10e-03 TCCCCAGGCC GTGGGTCAT GTAGAAACTG

ebv:112711-112751,numTag + 17 2.10e-03 GCCTCCGTAG ATGAGTCGG GCCAGGAAGC

ebv:85891-85931,numTags= + 10 2.26e-03 ACGCACATG CTCAGCCAG GTAAGTCTCC

ebv:11211-11251,numTags= - 24 2.26e-03 TTCAAGTC GTGGGCGAA TTAACTGAGC

ebv:86031-86071,numTags= + 29 2.49e-03 CGTTGAAGCA CTGTGCGTG GGC

ebv:70051-70091,numTags= + 7 2.49e-03 GACTGA AGGAGCGAT AGTTGAGACT

ebv:64091-64131,numTags= - 20 2.49e-03 TCCGAGTCTT TCGAGCCAG AGATGTCCAT

ebv:163051-163091,numTag + 25 2.49e-03 CATTGTTGCA AGGAGCGAT TTGGAGA

ebv:119091-119131,numTag - 20 2.49e-03 ATGGTCTCAG TTGAGCATT CACCTTTTAA

ebv:87131-87171,numTags= - 2 2.71e-03 GGCTTGAACA GCGAGCAAA T

ebv:169131-169171,numTag - 24 2.71e-03 ACCTCCAA ATGAGAGAT GACTCGGCGT

ebv:165911-165951,numTag - 25 2.71e-03 CAACAGT GTGTGTCAC GTGGCCACGT

ebv:146211-146251,numTag - 21 2.95e-03 TGGCCTTGAG GTCGGCGAT GATTTTCTCT

ebv:971-1011,numTags=324 + 16 3.18e-03 TTACTTGTTC TTTTGTAAT CGCAGCTCTA

ebv:87251-87291,numTags= + 2 3.18e-03 G CTCAGTCAT CTGGAATACC

ebv:129431-129471,numTag + 4 3.44e-03 AGC ATCGGCAAG ATTTTGACAT

ebv:95951-95991,numTags= - 18 3.75e-03 TTGAGGTTAT GCGTGCGGT AACCCATACG

ebv:4091-4131,numTags=11 - 16 3.75e-03 TTGGAGGCGG ACGAGCCGG TCTCGGGGCA

ebv:164451-164491,numTag + 15 3.75e-03 GCGTCACCGT CTCGGCGAT GGAGAGGCAG

ebv:142331-142371,numTag - 3 3.75e-03 GTTACAACCT TTGTGCATT AT

ebv:105471-105511,numTag - 14 3.75e-03 GGTCAAGAAC CTCGGCGAT GTCACGCTGT

ebv:90071-90111,numTags= + 8 4.03e-03 AGCGGAG GTTAGTAAA GGCATATGTG

ebv:57351-57391,numTags= + 25 4.03e-03 TTGTTGCCGA ACGAGTCAA GAATCAG

ebv:124471-124511,numTag + 25 4.03e-03 AGGTTTGGGA GTGGGCCAA TATTTGC

ebv:120911-120951,numTag - 26 4.03e-03 CCGACG ACGAGTCAA CTCCATAGGC

ebv:117871-117911,numTag + 18 4.03e-03 CCGCCTTCCC GCGTGCAAA CGTGGCGAGG

ebv:95871-95911,numTags= - 2 4.32e-03 ATGGCCTGAC ATCTGTGAC G

ebv:113951-113991,numTag + 3 4.65e-03 TT CTGTGCGGC CACGGTTGTG

ebv:109551-109591,numTag + 4 4.65e-03 CCA TTGAGTCGT CTCCCCTTTG

ebv:102651-102691,numTag + 27 4.65e-03 TGTGGTTTCC GTGTGCGTC GTGCC

ebv:1991-2031,numTags=20 + 25 4.65e-03 CTAGGAAACG GCGAGCAGG GTGAACA

ebv:173691-173731,numTag + 4 5.03e-03 GCC ACGAGTCGT AGTTGAGGCT

ebv:98931-98971,numTags= + 13 5.37e-03 GGCGGTGTGG GTTGGCGAC ATTGGCTTCT

ebv:62431-62471,numTags= + 24 5.37e-03 CTACCGGATG GCGGGTAAT ACATGCTA

ebv:4051-4091,numTags=10 + 13 6.11e-03 CGCGCGTGGC CTGGGCGTG AAGCTGACCT

ebv:138631-138671,numTag - 22 6.11e-03 GAAGCTGGAA TCGAGCGGG GGTGGAGGGG

ebv:137911-137951,numTag + 14 6.11e-03 GAGCGCTGCT TCGAGCAGT TCTGCCGCGT

ebv:110891-110931,numTag + 11 6.11e-03 GCGGTTAAAA GCGTGCGTG TTTATGCTGT

ebv:168311-168351,numTag - 19 6.52e-03 AAGGCGAACG CCGAGCCTT CCTGGACAAG

ebv:139451-139491,numTag - 22 7.78e-03 TTACCACCAG CTTTGTCAC GTTCGCCGAA

--------------------------------------------------------------------------------

--------------------------------------------------------------------------------

Motif 1 block diagrams

--------------------------------------------------------------------------------

SEQUENCE NAME POSITION P-VALUE MOTIF DIAGRAM

------------- ---------------- -------------

ebv:106351-106391,numTag 3.2e-06 19_[+1]_12

ebv:84631-84671,numTags= 9.8e-06 13_[+1]_18

ebv:177391-177431,numTag 9.8e-06 8_[+1]_23

ebv:127491-127531,numTag 9.8e-06 13_[+1]_18

ebv:83851-83891,numTags= 1.7e-05 15_[+1]_16

ebv:165011-165051,numTag 1.7e-05 3_[+1]_28

ebv:130291-130331,numTag 2e-05 6_[-1]_25

ebv:98231-98271,numTags= 2.6e-05 8_[+1]_23

ebv:78731-78771,numTags= 2.6e-05 9_[+1]_22

ebv:53711-53751,numTags= 2.6e-05 22_[+1]_9

ebv:79611-79651,numTags= 3.7e-05 6_[+1]_25

ebv:175331-175371,numTag 3.7e-05 18_[-1]_13

ebv:126771-126811,numTag 3.7e-05 24_[-1]_7

ebv:86531-86571,numTags= 4.5e-05 15_[+1]_16

ebv:66611-66651,numTags= 4.5e-05 5_[-1]_26

ebv:137131-137171,numTag 4.5e-05 23_[+1]_8

ebv:137971-138011,numTag 5.3e-05 22_[-1]_9

ebv:57271-57311,numTags= 6.4e-05 29_[-1]_2

ebv:87671-87711,numTags= 8.2e-05 14_[-1]_17

ebv:85431-85471,numTags= 8.2e-05 7_[-1]_24

ebv:58511-58551,numTags= 8.2e-05 5_[+1]_26

ebv:56751-56791,numTags= 8.2e-05 24_[+1]_7

ebv:166811-166851,numTag 8.2e-05 17_[+1]_14

ebv:119771-119811,numTag 8.2e-05 24_[-1]_7

ebv:176691-176731,numTag 0.00012 16_[-1]_15

ebv:150491-150531,numTag 0.00012 25_[+1]_6

ebv:114471-114511,numTag 0.00012 22_[+1]_9

ebv:9131-9171,numTags=92 0.00013 26_[-1]_5

ebv:49271-49311,numTags= 0.00013 15_[-1]_16

ebv:179031-179071,numTag 0.00013 4_[-1]_27

ebv:150951-150991,numTag 0.00013 19_[-1]_12

ebv:72411-72451,numTags= 0.00016 13_[+1]_18

ebv:67971-68011,numTags= 0.00016 7_[+1]_24

ebv:58171-58211,numTags= 0.00016 29_[+1]_2

ebv:9591-9631,numTags=37 0.00019 6_[+1]_25

ebv:76111-76151,numTags= 0.00019 12_[-1]_19

ebv:57211-57251,numTags= 0.00019 7_[-1]_24

ebv:179851-179891,numTag 0.00019 30_[+1]_1

ebv:162131-162171,numTag 0.00019 24_[+1]_7

ebv:148171-148211,numTag 0.00019 25_[-1]_6

ebv:77911-77951,numTags= 0.00022 29_[-1]_2

ebv:68271-68311,numTags= 0.00022 16_[-1]_15

ebv:2511-2551,numTags=56 0.00022 19_[-1]_12

ebv:143471-143511,numTag 0.00022 24_[-1]_7

ebv:131151-131191,numTag 0.00022 19_[+1]_12

ebv:115331-115371,numTag 0.00022 10_[+1]_21

ebv:81911-81951,numTags= 0.00025 18_[+1]_13

ebv:59431-59471,numTags= 0.00025 8_[+1]_23

ebv:129571-129611,numTag 0.00025 21_[+1]_10

ebv:125971-126011,numTag 0.00025 11_[+1]_20

ebv:90991-91031,numTags= 0.0003 8_[-1]_23

ebv:134451-134491,numTag 0.0003 13_[+1]_18

ebv:100471-100511,numTag 0.0003 5_[-1]_26

ebv:92671-92711,numTags= 0.00034 9_[+1]_22

ebv:67491-67531,numTags= 0.00034 20_[+1]_11

ebv:122791-122831,numTag 0.00034 16_[-1]_15

ebv:114591-114631,numTag 0.00034 24_[+1]_7

ebv:98391-98431,numTags= 0.00039 25_[+1]_6

ebv:8111-8151,numTags=29 0.00039 2_[+1]_29

ebv:71071-71111,numTags= 0.00039 12_[+1]_19

ebv:168391-168431,numTag 0.00039 23_[-1]_8

ebv:141371-141411,numTag 0.00039 22_[+1]_9

ebv:118931-118971,numTag 0.00039 15_[-1]_16

ebv:150171-150211,numTag 0.00044 6_[-1]_25

ebv:101451-101491,numTag 0.00044 12_[-1]_19

ebv:1531-1571,numTags=44 0.00044 7_[-1]_24

ebv:93211-93251,numTags= 0.00051 1_[+1]_30

ebv:61451-61491,numTags= 0.00051 16_[+1]_15

ebv:55271-55311,numTags= 0.00051 12_[+1]_19

ebv:5831-5871,numTags=14 0.00051 19_[+1]_12

ebv:172731-172771,numTag 0.00051 9_[+1]_22

ebv:147591-147631,numTag 0.00051 6_[+1]_25

ebv:53611-53651,numTags= 0.00058 17_[-1]_14

ebv:52831-52871,numTags= 0.00058 1_[+1]_30

ebv:71491-71531,numTags= 0.00065 27_[-1]_4

ebv:69011-69051,numTags= 0.00065 17_[+1]_14

ebv:174771-174811,numTag 0.00065 5_[-1]_26

ebv:170651-170691,numTag 0.00065 12_[-1]_19

ebv:133951-133991,numTag 0.00065 14_[+1]_17

ebv:110071-110111,numTag 0.00065 18_[+1]_13

ebv:48111-48151,numTags= 0.00074 5_[+1]_26

ebv:166431-166471,numTag 0.00074 13_[-1]_18

ebv:126231-126271,numTag 0.00074 7_[-1]_24

ebv:125671-125711,numTag 0.00074 28_[-1]_3

ebv:1711-1751,numTags=53 0.00074 14_[+1]_17

ebv:93951-93991,numTags= 0.00083 12_[+1]_19

ebv:171491-171531,numTag 0.00083 24_[+1]_7

ebv:147151-147191,numTag 0.00083 27_[-1]_4

ebv:139951-139991,numTag 0.00083 29_[-1]_2

ebv:128591-128631,numTag 0.00083 25_[-1]_6

ebv:102531-102571,numTag 0.00083 1_[+1]_30

ebv:98791-98831,numTags= 0.00094 8_[+1]_23

ebv:64591-64631,numTags= 0.00094 9_[+1]_22

ebv:110811-110851,numTag 0.00094 20_[-1]_11

ebv:101151-101191,numTag 0.00094 15_[-1]_16

ebv:11091-11131,numTags= 0.00094 9_[-1]_22

ebv:98031-98071,numTags= 0.001 8_[+1]_23

ebv:86171-86211,numTags= 0.001 11_[+1]_20

ebv:6971-7011,numTags=10 0.001 22_[-1]_9

ebv:124111-124151,numTag 0.001 6_[+1]_25

ebv:123931-123971,numTag 0.001 8_[-1]_23

ebv:122471-122511,numTag 0.001 3_[-1]_28

ebv:118791-118831,numTag 0.001 12_[-1]_19

ebv:9271-9311,numTags=57 0.0011 27_[-1]_4

ebv:169511-169551,numTag 0.0011 27_[+1]_4

ebv:144291-144331,numTag 0.0011 23_[+1]_8

ebv:142951-142991,numTag 0.0011 19_[-1]_12

ebv:72071-72111,numTags= 0.0013 26_[+1]_5

ebv:177991-178031,numTag 0.0013 1_[+1]_30

ebv:74971-75011,numTags= 0.0014 25_[-1]_6

ebv:4371-4411,numTags=67 0.0014 13_[+1]_18

ebv:128451-128491,numTag 0.0014 5_[+1]_26

ebv:100571-100611,numTag 0.0014 14_[-1]_17

ebv:1111-1151,numTags=64 0.0014 21_[-1]_10

ebv:175671-175711,numTag 0.0016 20_[+1]_11

ebv:173351-173391,numTag 0.0016 9_[+1]_22

ebv:140391-140431,numTag 0.0016 21_[-1]_10

ebv:125091-125131,numTag 0.0016 21_[-1]_10

ebv:92191-92231,numTags= 0.0017 24_[+1]_7

ebv:145211-145251,numTag 0.0017 30_[-1]_1

ebv:136711-136751,numTag 0.0017 19_[-1]_12

ebv:78851-78891,numTags= 0.0019 25_[-1]_6

ebv:64131-64171,numTags= 0.0019 23_[+1]_8

ebv:4671-4711,numTags=56 0.0019 21_[+1]_10

ebv:163311-163351,numTag 0.0019 1_[+1]_30

ebv:94271-94311,numTags= 0.0021 6_[-1]_25

ebv:76531-76571,numTags= 0.0021 7_[-1]_24

ebv:173491-173531,numTag 0.0021 15_[+1]_16

ebv:112711-112751,numTag 0.0021 16_[+1]_15

ebv:85891-85931,numTags= 0.0023 9_[+1]_22

ebv:11211-11251,numTags= 0.0023 23_[-1]_8

ebv:86031-86071,numTags= 0.0025 28_[+1]_3

ebv:70051-70091,numTags= 0.0025 6_[+1]_25

ebv:64091-64131,numTags= 0.0025 19_[-1]_12

ebv:163051-163091,numTag 0.0025 24_[+1]_7

ebv:119091-119131,numTag 0.0025 19_[-1]_12

ebv:87131-87171,numTags= 0.0027 1_[-1]_30

ebv:169131-169171,numTag 0.0027 23_[-1]_8

ebv:165911-165951,numTag 0.0027 24_[-1]_7

ebv:146211-146251,numTag 0.003 20_[-1]_11

ebv:971-1011,numTags=324 0.0032 15_[+1]_16

ebv:87251-87291,numTags= 0.0032 1_[+1]_30

ebv:129431-129471,numTag 0.0034 3_[+1]_28

ebv:95951-95991,numTags= 0.0037 17_[-1]_14

ebv:4091-4131,numTags=11 0.0037 15_[-1]_16

ebv:164451-164491,numTag 0.0037 14_[+1]_17

ebv:142331-142371,numTag 0.0037 2_[-1]_29

ebv:105471-105511,numTag 0.0037 13_[-1]_18

ebv:90071-90111,numTags= 0.004 7_[+1]_24

ebv:57351-57391,numTags= 0.004 24_[+1]_7

ebv:124471-124511,numTag 0.004 24_[+1]_7

ebv:120911-120951,numTag 0.004 25_[-1]_6

ebv:117871-117911,numTag 0.004 17_[+1]_14

ebv:95871-95911,numTags= 0.0043 1_[-1]_30

ebv:113951-113991,numTag 0.0047 2_[+1]_29

ebv:109551-109591,numTag 0.0047 3_[+1]_28

ebv:102651-102691,numTag 0.0047 26_[+1]_5

ebv:1991-2031,numTags=20 0.0047 24_[+1]_7

ebv:173691-173731,numTag 0.005 3_[+1]_28

ebv:98931-98971,numTags= 0.0054 12_[+1]_19

ebv:62431-62471,numTags= 0.0054 23_[+1]_8

ebv:4051-4091,numTags=10 0.0061 12_[+1]_19

ebv:138631-138671,numTag 0.0061 21_[-1]_10

ebv:137911-137951,numTag 0.0061 13_[+1]_18

ebv:110891-110931,numTag 0.0061 10_[+1]_21

ebv:168311-168351,numTag 0.0065 18_[-1]_13

ebv:139451-139491,numTag 0.0078 21_[-1]_10

--------------------------------------------------------------------------------

--------------------------------------------------------------------------------

Motif 1 in BLOCKS format

--------------------------------------------------------------------------------

BL MOTIF 1 width=9 seqs=167

ebv:106351-106391,numTag ( 20) ATGAGCGAT 1

ebv:84631-84671,numTags= ( 14) ATGAGCGAG 1

ebv:177391-177431,numTag ( 9) ATGAGCGAG 1

ebv:127491-127531,numTag ( 14) ATGAGCAAT 1

ebv:83851-83891,numTags= ( 16) GTGAGCGAT 1

ebv:165011-165051,numTag ( 4) GTGAGCGAT 1

ebv:130291-130331,numTag ( 7) ATGAGCCAT 1

ebv:98231-98271,numTags= ( 9) ATGAGTGAT 1

ebv:78731-78771,numTags= ( 10) ATGAGCAAG 1

ebv:53711-53751,numTags= ( 23) ATGAGCAAG 1

ebv:79611-79651,numTags= ( 7) ATGTGCGAG 1

ebv:175331-175371,numTag ( 19) CTGAGCGAT 1

ebv:126771-126811,numTag ( 25) CTGAGCGAT 1

ebv:86531-86571,numTags= ( 16) GTGAGCGAG 1

ebv:66611-66651,numTags= ( 6) GTGAGCGAG 1

ebv:137131-137171,numTag ( 24) GTGAGCAAT 1

ebv:137971-138011,numTag ( 23) ATGAGCCAG 1

ebv:57271-57311,numTags= ( 30) ATGTGCCAT 1

ebv:87671-87711,numTags= ( 15) CTGAGCGAG 1

ebv:85431-85471,numTags= ( 8) CTGAGCGAG 1

ebv:58511-58551,numTags= ( 6) GTGAGCCAT 1

ebv:56751-56791,numTags= ( 25) CTGAGCGAG 1

ebv:166811-166851,numTag ( 18) CTGAGCGAG 1

ebv:119771-119811,numTag ( 25) CTGAGCGAG 1

ebv:176691-176731,numTag ( 17) ATGAGTCAT 1

ebv:150491-150531,numTag ( 26) ATGAGTCAT 1

ebv:114471-114511,numTag ( 23) GTGTGCGAG 1

ebv:9131-9171,numTags=92 ( 27) ATTAGCAAT 1

ebv:49271-49311,numTags= ( 16) ATGAGTAAG 1

ebv:179031-179071,numTag ( 5) ATGAGTAAG 1

ebv:150951-150991,numTag ( 20) ATTAGCAAT 1

ebv:72411-72451,numTags= ( 14) ATGTGTAAT 1

ebv:67971-68011,numTags= ( 8) ATTTGCGAT 1

ebv:58171-58211,numTags= ( 30) CTGAGTGAT 1

ebv:9591-9631,numTags=37 ( 7) CTGTGCAAT 1

ebv:76111-76151,numTags= ( 13) GTGAGTAAT 1

ebv:57211-57251,numTags= ( 8) GTGTGCCAT 1

ebv:179851-179891,numTag ( 31) CTGTGCGAG 1

ebv:162131-162171,numTag ( 25) CTGTGCAAT 1

ebv:148171-148211,numTag ( 26) CTGTGCAAT 1

ebv:77911-77951,numTags= ( 30) ACGAGCGAG 1

ebv:68271-68311,numTags= ( 17) ATGAGCGGT 1

ebv:2511-2551,numTags=56 ( 20) ATGAGCGGT 1

ebv:143471-143511,numTag ( 25) ATGAGTCAG 1

ebv:131151-131191,numTag ( 20) ATGAGCGGT 1

ebv:115331-115371,numTag ( 11) ACGAGCAAT 1

ebv:81911-81951,numTags= ( 19) ATGTGTCAT 1

ebv:59431-59471,numTags= ( 9) ATTAGTGAT 1

ebv:129571-129611,numTag ( 22) CTGAGCCAG 1

ebv:125971-126011,numTag ( 12) ATTAGCAAG 1

ebv:90991-91031,numTags= ( 9) ATTTGCGAG 1

ebv:134451-134491,numTag ( 14) ATGGGCGAG 1

ebv:100471-100511,numTag ( 6) ATGGGCGAG 1

ebv:92671-92711,numTags= ( 10) ACGAGCCAT 1

ebv:67491-67531,numTags= ( 21) ATGAGCGTT 1

ebv:122791-122831,numTag ( 17) TTGAGCAAG 1

ebv:114591-114631,numTag ( 25) ATGAGCGTT 1

ebv:98391-98431,numTags= ( 26) ATGAGCGGG 1

ebv:8111-8151,numTags=29 ( 3) GTGTGTAAT 1

ebv:71071-71111,numTags= ( 13) GTTTGCGAT 1

ebv:168391-168431,numTag ( 24) ATGAGCGGG 1

ebv:141371-141411,numTag ( 23) ATGAGCGGG 1

ebv:118931-118971,numTag ( 16) GTGGGCGAT 1

ebv:150171-150211,numTag ( 7) ACGTGCAAT 1

ebv:101451-101491,numTag ( 13) ATGTGTCAG 1

ebv:1531-1571,numTags=44 ( 8) ATGTGTCAG 1

ebv:93211-93251,numTags= ( 2) GTCAGCGAT 1

ebv:61451-61491,numTags= ( 17) GTGAGCGGT 1

ebv:55271-55311,numTags= ( 13) ATTTGCAAG 1

ebv:5831-5871,numTags=14 ( 20) ATGTGCAAA 1

ebv:172731-172771,numTag ( 10) GTGAGCGGT 1

ebv:147591-147631,numTag ( 7) GTGAGCGGT 1

ebv:53611-53651,numTags= ( 18) GTGTGTCAT 1

ebv:52831-52871,numTags= ( 2) CTGTGTAAT 1

ebv:71491-71531,numTags= ( 28) ATGAGTAAC 1

ebv:69011-69051,numTags= ( 18) GTTTGCGAG 1

ebv:174771-174811,numTag ( 6) GTTTGCGAG 1

ebv:170651-170691,numTag ( 13) GTGGGCGAG 1

ebv:133951-133991,numTag ( 15) ATTAGTCAT 1

ebv:110071-110111,numTag ( 19) ATTAGCGAC 1

ebv:48111-48151,numTags= ( 6) ACGTGCAAG 1

ebv:166431-166471,numTag ( 14) ATGTGCGGG 1

ebv:126231-126271,numTag ( 8) ATGTGTGAC 1

ebv:125671-125711,numTag ( 29) GTGAGCCAC 1

ebv:1711-1751,numTags=53 ( 15) ACGTGCAAG 1

ebv:93951-93991,numTags= ( 13) TTGAGCGAC 1

ebv:171491-171531,numTag ( 25) GTCAGCGAG 1

ebv:147151-147191,numTag ( 28) CCGTGCGAT 1

ebv:139951-139991,numTag ( 30) CTGTGTCAT 1

ebv:128591-128631,numTag ( 26) GTCAGCGAG 1

ebv:102531-102571,numTag ( 2) GCGAGCAAG 1

ebv:98791-98831,numTags= ( 9) TTTAGCAAT 1

ebv:64591-64631,numTags= ( 10) GCGTGCGAG 1

ebv:110811-110851,numTag ( 21) GCGTGCGAG 1

ebv:101151-101191,numTag ( 16) ATGAGTCAC 1

ebv:11091-11131,numTags= ( 10) CTTTGCGAG 1

ebv:98031-98071,numTags= ( 9) ACGAGCGAA 1

ebv:86171-86211,numTags= ( 12) GTGTGCAAA 1

ebv:6971-7011,numTags=10 ( 23) CTGAGCGTT 1

ebv:124111-124151,numTag ( 7) CTGAGCGTT 1

ebv:123931-123971,numTag ( 9) ATGAGTAGT 1

ebv:122471-122511,numTag ( 4) ATGAGTCAA 1

ebv:118791-118831,numTag ( 13) TTTTGCGAT 1

ebv:9271-9311,numTags=57 ( 28) ATGTGTAAC 1

ebv:169511-169551,numTag ( 28) GCGAGCCAG 1

ebv:144291-144331,numTag ( 24) ATTTGTCAT 1

ebv:142951-142991,numTag ( 20) CTCAGCGAG 1

ebv:72071-72111,numTags= ( 27) CCGTGCGAG 1

ebv:177991-178031,numTag ( 2) GTTAGTCAT 1

ebv:74971-75011,numTags= ( 26) ATGAGCGGC 1

ebv:4371-4411,numTags=67 ( 14) ATCAGCGAC 1

ebv:128451-128491,numTag ( 6) GTGAGTAAA 1

ebv:100571-100611,numTag ( 15) TCGTGCGAT 1

ebv:1111-1151,numTags=64 ( 22) ACCAGCGAT 1

ebv:175671-175711,numTag ( 21) CTGAGCGTG 1

ebv:173351-173391,numTag ( 10) TTGGGCGAG 1

ebv:140391-140431,numTag ( 22) GTTGGCGAT 1

ebv:125091-125131,numTag ( 22) TTGGGCAAT 1

ebv:92191-92231,numTags= ( 25) GTGAGTCAC 1

ebv:145211-145251,numTag ( 31) ACGTGCGAA 1

ebv:136711-136751,numTag ( 20) ACGTGCGAA 1

ebv:78851-78891,numTags= ( 26) ATTGGCAAG 1

ebv:64131-64171,numTags= ( 24) TTCAGCGAG 1

ebv:4671-4711,numTags=56 ( 22) CTTTGCCAG 1

ebv:163311-163351,numTag ( 2) CTTTGCCAG 1

ebv:94271-94311,numTags= ( 7) ATCAGCAAC 1

ebv:76531-76571,numTags= ( 8) GTGTGTAAC 1

ebv:173491-173531,numTag ( 16) GTGGGTCAT 1

ebv:112711-112751,numTag ( 17) ATGAGTCGG 1

ebv:85891-85931,numTags= ( 10) CTCAGCCAG 1

ebv:11211-11251,numTags= ( 24) GTGGGCGAA 1

ebv:86031-86071,numTags= ( 29) CTGTGCGTG 1

ebv:70051-70091,numTags= ( 7) AGGAGCGAT 1

ebv:64091-64131,numTags= ( 20) TCGAGCCAG 1

ebv:163051-163091,numTag ( 25) AGGAGCGAT 1

ebv:119091-119131,numTag ( 20) TTGAGCATT 1

ebv:87131-87171,numTags= ( 2) GCGAGCAAA 1

ebv:169131-169171,numTag ( 24) ATGAGAGAT 1

ebv:165911-165951,numTag ( 25) GTGTGTCAC 1

ebv:146211-146251,numTag ( 21) GTCGGCGAT 1

ebv:971-1011,numTags=324 ( 16) TTTTGTAAT 1

ebv:87251-87291,numTags= ( 2) CTCAGTCAT 1

ebv:129431-129471,numTag ( 4) ATCGGCAAG 1

ebv:95951-95991,numTags= ( 18) GCGTGCGGT 1

ebv:4091-4131,numTags=11 ( 16) ACGAGCCGG 1

ebv:164451-164491,numTag ( 15) CTCGGCGAT 1

ebv:142331-142371,numTag ( 3) TTGTGCATT 1

ebv:105471-105511,numTag ( 14) CTCGGCGAT 1

ebv:90071-90111,numTags= ( 8) GTTAGTAAA 1

ebv:57351-57391,numTags= ( 25) ACGAGTCAA 1

ebv:124471-124511,numTag ( 25) GTGGGCCAA 1

ebv:120911-120951,numTag ( 26) ACGAGTCAA 1

ebv:117871-117911,numTag ( 18) GCGTGCAAA 1

ebv:95871-95911,numTags= ( 2) ATCTGTGAC 1

ebv:113951-113991,numTag ( 3) CTGTGCGGC 1

ebv:109551-109591,numTag ( 4) TTGAGTCGT 1

ebv:102651-102691,numTag ( 27) GTGTGCGTC 1

ebv:1991-2031,numTags=20 ( 25) GCGAGCAGG 1

ebv:173691-173731,numTag ( 4) ACGAGTCGT 1

ebv:98931-98971,numTags= ( 13) GTTGGCGAC 1

ebv:62431-62471,numTags= ( 24) GCGGGTAAT 1

ebv:4051-4091,numTags=10 ( 13) CTGGGCGTG 1

ebv:138631-138671,numTag ( 22) TCGAGCGGG 1

ebv:137911-137951,numTag ( 14) TCGAGCAGT 1

ebv:110891-110931,numTag ( 11) GCGTGCGTG 1

ebv:168311-168351,numTag ( 19) CCGAGCCTT 1

ebv:139451-139491,numTag ( 22) CTTTGTCAC 1

//

--------------------------------------------------------------------------------

--------------------------------------------------------------------------------

Motif 1 position-specific scoring matrix

--------------------------------------------------------------------------------

log-odds matrix: alength= 4 w= 9 n= 6400 bayes= 6.22207 E= 1.7e-039

96 -49 2 -131

-1403 -58 -453 185

-1403 -163 145 -57

136 -1403 -137 54

-1403 -1403 185 -1403

-521 142 -1403 18

25 -25 86 -1403

185 -1403 -114 -163

-141 -137 42 98

--------------------------------------------------------------------------------

--------------------------------------------------------------------------------

Motif 1 position-specific probability matrix

--------------------------------------------------------------------------------

letter-probability matrix: alength= 4 w= 9 nsites= 167 E= 1.7e-039

0.431138 0.197605 0.281437 0.089820

0.000000 0.185629 0.011976 0.802395

0.000000 0.089820 0.760479 0.149701

0.568862 0.000000 0.107784 0.323353

0.000000 0.000000 1.000000 0.000000

0.005988 0.742515 0.000000 0.251497

0.263473 0.233533 0.502994 0.000000

0.802395 0.000000 0.125749 0.071856

0.083832 0.107784 0.371257 0.437126

--------------------------------------------------------------------------------

--------------------------------------------------------------------------------

Motif 1 regular expression

--------------------------------------------------------------------------------

[AG]TG[AT]G[CT][GAC]A[TG]

--------------------------------------------------------------------------------

********************************************************************************

MEME - Motif discovery tool

********************************************************************************

MEME version 4.4.0 (Release date: Tue Apr 27 10:09:30 EST 2010)

For further information on how to interpret these results or to get

a copy of the MEME software please access http://meme.nbcr.net.

This file may be used as input to the MAST algorithm for searching

sequence databases for matches to groups of motifs. MAST is available

for interactive use and downloading at http://meme.nbcr.net.

********************************************************************************

********************************************************************************

REFERENCE

********************************************************************************

If you use this program in your research, please cite:

Timothy L. Bailey and Charles Elkan,

"Fitting a mixture model by expectation maximization to discover

motifs in biopolymers", Proceedings of the Second International

Conference on Intelligent Systems for Molecular Biology, pp. 28-36,

AAAI Press, Menlo Park, California, 1994.

********************************************************************************

********************************************************************************

TRAINING SET

********************************************************************************

DATAFILE= **46312.QuEST.params.old.peaks.meme**

ALPHABET= ACGT

Sequence name Weight Length Sequence name Weight Length

------------- ------ ------ ------------- ------ ------

ebv:114242-114900 1.0000 658 ebv:106126-106579 1.0000 453

ebv:79396-79867 1.0000 471 ebv:86005-86756 1.0000 751

ebv:177171-177615 1.0000 444 ebv:126570-126986 1.0000 416

ebv:164807-165214 1.0000 407 ebv:109902-110308 1.0000 406

ebv:161935-162298 1.0000 363 ebv:84350-84802 1.0000 452

ebv:83676-84041 1.0000 365 ebv:166640-166967 1.0000 327

ebv:110629-111123 1.0000 494 ebv:66442-66774 1.0000 332

ebv:147967-148363 1.0000 396 ebv:145053-145344 1.0000 291

ebv:67792-68122 1.0000 330 ebv:53437-53839 1.0000 402

ebv:176541-176815 1.0000 274 ebv:122344-122869 1.0000 525

ebv:49077-49424 1.0000 347 ebv:56613-56899 1.0000 286

ebv:119625-119934 1.0000 309 ebv:85292-85589 1.0000 297

ebv:52737-53008 1.0000 271 ebv:64473-64710 1.0000 237

ebv:9107-9270 1.0000 163 ebv:127346-127560 1.0000 214

ebv:150337-150695 1.0000 358 ebv:147010-147325 1.0000 315

ebv:150724-150976 1.0000 252 ebv:2440-2626 1.0000 186

ebv:118717-118899 1.0000 182 ebv:87610-87796 1.0000 186

ebv:129518-129739 1.0000 221 ebv:137062-137258 1.0000 196

ebv:4263-4402 1.0000 139 ebv:147487-147715 1.0000 228

ebv:77811-77973 1.0000 162 ebv:90892-91091 1.0000 199

ebv:172693-172866 1.0000 173 ebv:97959-98189 1.0000 230

ebv:136661-136793 1.0000 132 ebv:78693-78782 1.0000 89

ebv:61370-61478 1.0000 108 ebv:58438-58517 1.0000 79

********************************************************************************

********************************************************************************

COMMAND LINE SUMMARY

********************************************************************************

This information can also be useful in the event you wish to report a

problem with the MEME software.

command: meme sequences -sf 46312.QuEST.params.old.peaks.meme -dna -mod zoops -nmotifs 3 -minw 6 -maxw 50 -time 7200 -maxsize 60000 -revcomp -oc . -nostatus

model: mod= zoops nmotifs= 3 evt= inf

object function= E-value of product of p-values

width: minw= 6 maxw= 50 minic= 0.00

width: wg= 11 ws= 1 endgaps= yes

nsites: minsites= 2 maxsites= 46 wnsites= 0.8

theta: prob= 1 spmap= uni spfuzz= 0.5

global: substring= yes branching= no wbranch= no

em: prior= dirichlet b= 0.01 maxiter= 50

distance= 1e-05

data: n= 14116 N= 46

strands: + -

sample: seed= 0 seqfrac= 1

Letter frequencies in dataset:

A 0.213 C 0.287 G 0.287 T 0.213

Background letter frequencies (from dataset with add-one prior applied):

A 0.213 C 0.287 G 0.287 T 0.213

********************************************************************************

********************************************************************************

MOTIF 1 width = 8 sites = 46 llr = 343 E-value = 3.0e-006

********************************************************************************

--------------------------------------------------------------------------------

Motif 1 Description

--------------------------------------------------------------------------------

Simplified A 4::::129

pos.-specific C 515:a:8:

probability G ::1a:::1

matrix T :94::9::

bits 2.2

2.0

1.8 * *

1.6 * *** *

Relative 1.3 * *****

Entropy 1.1 * *****

(10.8 bits) 0.9 * *****

0.7 ********

0.4 ********

0.2 ********

0.0 --------

Multilevel CTCGCTCA

consensus A T

sequence

--------------------------------------------------------------------------------

--------------------------------------------------------------------------------

Motif 1 sites sorted by position p-value

--------------------------------------------------------------------------------

Sequence name Strand Start P-value Site

------------- ------ ----- --------- --------

ebv:137062-137258 - 94 1.04e-05 CGTCCCCACC ATTGCTCA CGGGGACGGA

ebv:150724-150976 + 180 1.04e-05 GCTATAAGGA ATTGCTCA GGCCAAAGTT

ebv:127346-127560 - 160 1.04e-05 GGTCAGGGGA ATTGCTCA TATTTTTAGC

ebv:109902-110308 - 207 1.04e-05 CGCATGTCTG ATTGCTCA CCAGGTAAAT

ebv:83676-84041 - 192 2.43e-05 GTCACATTGC ATCGCTCA CCCCTGTGCA

ebv:164807-165214 - 209 2.43e-05 GATTGCCAAG ATCGCTCA CATCCCCTGC

ebv:126570-126986 + 226 2.43e-05 GCGTAGAGGC ATCGCTCA GCACCCAGAG

ebv:106126-106579 - 246 2.43e-05 CTGTGATAAA ATCGCTCA TAAGCTTAGT

ebv:78693-78782 - 49 3.83e-05 CCCCTGGGCC CTTGCTCA TGATGTCAAC

ebv:122344-122869 + 464 3.83e-05 AGATGTAGCA CTTGCTCA AAAGGCAGAG

ebv:53437-53839 - 298 3.83e-05 CGTCCTTATT CTTGCTCA TATTTCCACC

ebv:79396-79867 - 364 3.83e-05 TCGGAACTCT CTTGCTCA AAGAAATTAG

ebv:87610-87796 + 76 5.71e-05 CCTCGAGCGC CTCGCTCA GCCCACTATC

ebv:85292-85589 + 147 5.71e-05 CAAAGAGCTG CTCGCTCA GGCTGTTCTG

ebv:119625-119934 + 171 5.71e-05 CGCTGGTCTT CTCGCTCA GCGTGTTCCT

ebv:56613-56899 - 164 5.71e-05 TGTGGAAAAT CTCGCTCA GAGCTGGGAA

ebv:66442-66774 + 175 5.71e-05 GATCCACGTA CTCGCTCA CGATGGCCGT

ebv:166640-166967 - 190 5.71e-05 GATCGACGAG CTCGCTCA GCAACATGCC

ebv:84350-84802 - 296 5.71e-05 GCATCTGGCC CTCGCTCA TGAGCACCCT

ebv:177171-177615 - 230 5.71e-05 CACACCTGTC CTCGCTCA TCTTTCCACA

ebv:86005-86756 - 543 5.71e-05 GGGCCCTGCG CTCGCTCA CGCCCCTCGT

ebv:9107-9270 + 51 6.48e-05 TATAAACACT ATTGCTAA TGCCCTCTTG

ebv:110629-111123 + 435 1.13e-04 CCACTTTGTC CTCGCTAA CAACTTTTTG

ebv:147487-147715 + 8 1.32e-04 GGGTGAC CTGGCTCA GGTCCGGCGC

ebv:129518-129739 - 76 1.32e-04 TCAGGCTACC CTGGCTCA GCGAGGAGGT

ebv:147010-147325 - 116 1.32e-04 GTGCGAGGAA CTGGCTCA GCTTGGGGCC

ebv:147967-148363 + 230 1.43e-04 CGATTGCGAT ATTGCACA GAGTCAGCGC

ebv:161935-162298 - 222 1.43e-04 CTGGGGTTTG ATTGCACA GAGTCTCTCG

ebv:114242-114900 - 253 1.89e-04 ACGAAACCGG CTCGCACA CGAGTCATCG

ebv:90892-91091 + 104 2.14e-04 CAAATGTCCG ATTGCTCG CAAATGCACC

ebv:52737-53008 + 114 2.14e-04 TACTTTAAGG TTTGCTCA GGAGTGGGGG

ebv:77811-77973 + 130 3.19e-04 GACCCTCCCT CTCGCTCG TTAAACAAGG

ebv:145053-145344 - 102 3.19e-04 GGTTGGCCGT GTTGCTCA TGGCCGTCTT

ebv:61370-61478 - 99 3.56e-04 AC ACCGCTCA CCAGGTGGCA

ebv:172693-172866 - 49 3.56e-04 CAGCTGTTTT ACCGCTCA CTGCTGACCA

ebv:2440-2626 + 91 3.56e-04 GGACACACGA ACCGCTCA TAGTTTGGAG

ebv:49077-49424 + 210 4.39e-04 AATGCACCCT CTTACTCA TCAAAGCACC

ebv:118717-118899 + 87 4.85e-04 TCCCGCTCCC ATCGCAAA AAGTTGAGTT

ebv:67792-68122 - 188 4.85e-04 AGAAAAGTAC ATCGCAAA TGCAGGGTCG

ebv:150337-150695 + 54 5.60e-04 AGACCGACTC ATTGCTAG GAACGCTGCA

ebv:97959-98189 + 202 6.85e-04 GTGCGGAGGA ACTGCTAA ACAGGAAAGG

ebv:4263-4402 - 12 7.57e-04 TCGTGGCCAC GTCGCTAA AGAGCTGCTC

ebv:136661-136793 - 22 8.33e-04 CCGGGGAGAG CGTGCTCA AGGGGGTCAG

ebv:176541-176815 + 167 9.86e-04 TCTCCACGGG ATGACTCA TCTCAACACA

ebv:64473-64710 - 129 1.08e-03 CATGCTGCTC CTCGCACG CCGGTGCTTC

ebv:58438-58517 + 54 2.24e-03 GTCAGGGGGT TTGGCTCG TTTGCGCTCG

--------------------------------------------------------------------------------

--------------------------------------------------------------------------------

Motif 1 block diagrams

--------------------------------------------------------------------------------

SEQUENCE NAME POSITION P-VALUE MOTIF DIAGRAM

------------- ---------------- -------------

ebv:137062-137258 1e-05 93_[-1]_95

ebv:150724-150976 1e-05 179_[+1]_65

ebv:127346-127560 1e-05 159_[-1]_47

ebv:109902-110308 1e-05 206_[-1]_192

ebv:83676-84041 2.4e-05 191_[-1]_166

ebv:164807-165214 2.4e-05 208_[-1]_191

ebv:126570-126986 2.4e-05 225_[+1]_183

ebv:106126-106579 2.4e-05 245_[-1]_200

ebv:78693-78782 3.8e-05 48_[-1]_33

ebv:122344-122869 3.8e-05 463_[+1]_54

ebv:53437-53839 3.8e-05 297_[-1]_97

ebv:79396-79867 3.8e-05 363_[-1]_100

ebv:87610-87796 5.7e-05 75_[+1]_103

ebv:85292-85589 5.7e-05 146_[+1]_143

ebv:119625-119934 5.7e-05 170_[+1]_131

ebv:56613-56899 5.7e-05 163_[-1]_115

ebv:66442-66774 5.7e-05 174_[+1]_150

ebv:166640-166967 5.7e-05 189_[-1]_130

ebv:84350-84802 5.7e-05 295_[-1]_149

ebv:177171-177615 5.7e-05 229_[-1]_207

ebv:86005-86756 5.7e-05 542_[-1]_201

ebv:9107-9270 6.5e-05 50_[+1]_105

ebv:110629-111123 0.00011 434_[+1]_52

ebv:147487-147715 0.00013 7_[+1]_213

ebv:129518-129739 0.00013 75_[-1]_138

ebv:147010-147325 0.00013 115_[-1]_192

ebv:147967-148363 0.00014 229_[+1]_159

ebv:161935-162298 0.00014 221_[-1]_134

ebv:114242-114900 0.00019 252_[-1]_398

ebv:90892-91091 0.00021 103_[+1]_88

ebv:52737-53008 0.00021 113_[+1]_150

ebv:77811-77973 0.00032 129_[+1]_25

ebv:145053-145344 0.00032 101_[-1]_182

ebv:61370-61478 0.00036 98_[-1]_2

ebv:172693-172866 0.00036 48_[-1]_117

ebv:2440-2626 0.00036 90_[+1]_88

ebv:49077-49424 0.00044 209_[+1]_130

ebv:118717-118899 0.00049 86_[+1]_88

ebv:67792-68122 0.00049 187_[-1]_135

ebv:150337-150695 0.00056 53_[+1]_297

ebv:97959-98189 0.00068 201_[+1]_21

ebv:4263-4402 0.00076 11_[-1]_120

ebv:136661-136793 0.00083 21_[-1]_103

ebv:176541-176815 0.00099 166_[+1]_100

ebv:64473-64710 0.0011 128_[-1]_101

ebv:58438-58517 0.0022 53_[+1]_18

--------------------------------------------------------------------------------

--------------------------------------------------------------------------------

Motif 1 in BLOCKS format

--------------------------------------------------------------------------------

BL MOTIF 1 width=8 seqs=46

ebv:137062-137258 ( 94) ATTGCTCA 1

ebv:150724-150976 ( 180) ATTGCTCA 1

ebv:127346-127560 ( 160) ATTGCTCA 1

ebv:109902-110308 ( 207) ATTGCTCA 1

ebv:83676-84041 ( 192) ATCGCTCA 1

ebv:164807-165214 ( 209) ATCGCTCA 1

ebv:126570-126986 ( 226) ATCGCTCA 1

ebv:106126-106579 ( 246) ATCGCTCA 1

ebv:78693-78782 ( 49) CTTGCTCA 1

ebv:122344-122869 ( 464) CTTGCTCA 1

ebv:53437-53839 ( 298) CTTGCTCA 1

ebv:79396-79867 ( 364) CTTGCTCA 1

ebv:87610-87796 ( 76) CTCGCTCA 1

ebv:85292-85589 ( 147) CTCGCTCA 1

ebv:119625-119934 ( 171) CTCGCTCA 1

ebv:56613-56899 ( 164) CTCGCTCA 1

ebv:66442-66774 ( 175) CTCGCTCA 1

ebv:166640-166967 ( 190) CTCGCTCA 1

ebv:84350-84802 ( 296) CTCGCTCA 1

ebv:177171-177615 ( 230) CTCGCTCA 1

ebv:86005-86756 ( 543) CTCGCTCA 1

ebv:9107-9270 ( 51) ATTGCTAA 1

ebv:110629-111123 ( 435) CTCGCTAA 1

ebv:147487-147715 ( 8) CTGGCTCA 1

ebv:129518-129739 ( 76) CTGGCTCA 1

ebv:147010-147325 ( 116) CTGGCTCA 1

ebv:147967-148363 ( 230) ATTGCACA 1

ebv:161935-162298 ( 222) ATTGCACA 1

ebv:114242-114900 ( 253) CTCGCACA 1

ebv:90892-91091 ( 104) ATTGCTCG 1

ebv:52737-53008 ( 114) TTTGCTCA 1

ebv:77811-77973 ( 130) CTCGCTCG 1

ebv:145053-145344 ( 102) GTTGCTCA 1

ebv:61370-61478 ( 99) ACCGCTCA 1

ebv:172693-172866 ( 49) ACCGCTCA 1

ebv:2440-2626 ( 91) ACCGCTCA 1

ebv:49077-49424 ( 210) CTTACTCA 1

ebv:118717-118899 ( 87) ATCGCAAA 1

ebv:67792-68122 ( 188) ATCGCAAA 1

ebv:150337-150695 ( 54) ATTGCTAG 1

ebv:97959-98189 ( 202) ACTGCTAA 1

ebv:4263-4402 ( 12) GTCGCTAA 1

ebv:136661-136793 ( 22) CGTGCTCA 1

ebv:176541-176815 ( 167) ATGACTCA 1

ebv:64473-64710 ( 129) CTCGCACG 1

ebv:58438-58517 ( 54) TTGGCTCG 1

//

--------------------------------------------------------------------------------

--------------------------------------------------------------------------------

Motif 1 position-specific scoring matrix

--------------------------------------------------------------------------------

log-odds matrix: alength= 4 w= 8 n= 13794 bayes= 8.22337 E= 3.0e-006

103 74 -272 -229

-1217 -172 -372 207

-1217 80 -140 88

-229 -1217 174 -1217

-1217 180 -1217 -1217

-71 -1217 -1217 203

-48 156 -1217 -1217

207 -1217 -140 -1217

--------------------------------------------------------------------------------

--------------------------------------------------------------------------------

Motif 1 position-specific probability matrix

--------------------------------------------------------------------------------

letter-probability matrix: alength= 4 w= 8 nsites= 46 E= 3.0e-006

0.434783 0.478261 0.043478 0.043478

0.000000 0.086957 0.021739 0.891304

0.000000 0.500000 0.108696 0.391304

0.043478 0.000000 0.956522 0.000000

0.000000 1.000000 0.000000 0.000000

0.130435 0.000000 0.000000 0.869565

0.152174 0.847826 0.000000 0.000000

0.891304 0.000000 0.108696 0.000000

--------------------------------------------------------------------------------

--------------------------------------------------------------------------------

Motif 1 regular expression

--------------------------------------------------------------------------------

[CA]T[CT]GCTCA

--------------------------------------------------------------------------------

********************************************************************************

MEME - Motif discovery tool

********************************************************************************

MEME version 4.4.0 (Release date: Tue Apr 27 10:09:30 EST 2010)

For further information on how to interpret these results or to get

a copy of the MEME software please access http://meme.nbcr.net.

This file may be used as input to the MAST algorithm for searching

sequence databases for matches to groups of motifs. MAST is available

for interactive use and downloading at http://meme.nbcr.net.

********************************************************************************

********************************************************************************

REFERENCE

********************************************************************************

If you use this program in your research, please cite:

Timothy L. Bailey and Charles Elkan,

"Fitting a mixture model by expectation maximization to discover

motifs in biopolymers", Proceedings of the Second International

Conference on Intelligent Systems for Molecular Biology, pp. 28-36,

AAAI Press, Menlo Park, California, 1994.

********************************************************************************

********************************************************************************

TRAINING SET

********************************************************************************

DATAFILE= **GFP95.8.SISSRs.out.meme**

ALPHABET= ACGT

Sequence name Weight Length Sequence name Weight Length

------------- ------ ------ ------------- ------ ------

ebv:106351-106391,numTag 1.0000 40 ebv:110091-110131,numTag 1.0000 40

ebv:110831-110871,numTag 1.0000 40 ebv:114491-114531,numTag 1.0000 40

ebv:119791-119831,numTag 1.0000 40 ebv:122471-122511,numTag 1.0000 40

ebv:126791-126831,numTag 1.0000 40 ebv:127491-127531,numTag 1.0000 40

ebv:129591-129631,numTag 1.0000 40 ebv:129771-129811,numTag 1.0000 40

ebv:131351-131391,numTag 1.0000 40 ebv:137151-137191,numTag 1.0000 40

ebv:145211-145251,numTag 1.0000 40 ebv:148191-148231,numTag 1.0000 40

ebv:150511-150551,numTag 1.0000 40 ebv:150631-150671,numTag 1.0000 40

ebv:150951-150991,numTag 1.0000 40 ebv:162131-162171,numTag 1.0000 40

ebv:165011-165051,numTag 1.0000 40 ebv:166811-166851,numTag 1.0000 40

ebv:174751-174791,numTag 1.0000 40 ebv:175331-175371,numTag 1.0000 40

ebv:176691-176731,numTag 1.0000 40 ebv:177391-177431,numTag 1.0000 40

ebv:49251-49291,numTags= 1.0000 40 ebv:52831-52871,numTags= 1.0000 40

ebv:53611-53651,numTags= 1.0000 40 ebv:53711-53751,numTags= 1.0000 40

ebv:56771-56811,numTags= 1.0000 40 ebv:58491-58531,numTags= 1.0000 40

ebv:64591-64631,numTags= 1.0000 40 ebv:66611-66651,numTags= 1.0000 40

ebv:67951-67991,numTags= 1.0000 40 ebv:71511-71551,numTags= 1.0000 40

ebv:72411-72451,numTags= 1.0000 40 ebv:76111-76151,numTags= 1.0000 40

ebv:78731-78771,numTags= 1.0000 40 ebv:79631-79671,numTags= 1.0000 40

ebv:83851-83891,numTags= 1.0000 40 ebv:84631-84671,numTags= 1.0000 40

ebv:86531-86571,numTags= 1.0000 40 ebv:9151-9191,numTags=11 1.0000 40

********************************************************************************

********************************************************************************

COMMAND LINE SUMMARY

********************************************************************************

This information can also be useful in the event you wish to report a

problem with the MEME software.

command: meme sequences -sf GFP95.8.SISSRs.out.meme -dna -mod zoops -nmotifs 3 -minw 6 -maxw 50 -time 7200 -maxsize 60000 -revcomp -oc . -nostatus

model: mod= zoops nmotifs= 3 evt= inf

object function= E-value of product of p-values

width: minw= 6 maxw= 40 minic= 0.00

width: wg= 11 ws= 1 endgaps= yes

nsites: minsites= 2 maxsites= 42 wnsites= 0.8

theta: prob= 1 spmap= uni spfuzz= 0.5

global: substring= yes branching= no wbranch= no

em: prior= dirichlet b= 0.01 maxiter= 50

distance= 1e-05

data: n= 1680 N= 42

strands: + -

sample: seed= 0 seqfrac= 1

Letter frequencies in dataset:

A 0.220 C 0.280 G 0.280 T 0.220

Background letter frequencies (from dataset with add-one prior applied):

A 0.220 C 0.280 G 0.280 T 0.220

********************************************************************************

********************************************************************************

MOTIF 1 width = 9 sites = 39 llr = 296 E-value = 2.9e-034

********************************************************************************

--------------------------------------------------------------------------------

Motif 1 Description

--------------------------------------------------------------------------------

Simplified A 4::7::3a1

pos.-specific C 31:::82::

probability G 3:9:a:5:4

matrix T :913:2::5

bits 2.2

2.0 * *

1.7 * * *

1.5 * * *

Relative 1.3 **** *

Entropy 1.1 ***** *

(10.9 bits) 0.9 ***** *

0.7 ***** **

0.4 *********

0.2 *********

0.0 ---------

Multilevel ATGAGCGAT

consensus G T A G

sequence C C

--------------------------------------------------------------------------------

--------------------------------------------------------------------------------

Motif 1 sites sorted by position p-value

--------------------------------------------------------------------------------

Sequence name Strand Start P-value Site

------------- ------ ----- --------- ---------

ebv:106351-106391,numTag + 20 3.17e-06 AACTAAGCTT ATGAGCGAT TTTATCACAG

ebv:127491-127531,numTag + 14 5.66e-06 GGCTAAAAAT ATGAGCAAT TCCCCTGACC

ebv:83851-83891,numTags= + 16 9.69e-06 CTGCACAGGG GTGAGCGAT GCAATGTGAC

ebv:165011-165051,numTag + 4 9.69e-06 GAT GTGAGCGAT CTTGGCAATC

ebv:84631-84671,numTags= + 14 1.69e-05 CAGGGTGCTC ATGAGCGAG GGCCAGATGC

ebv:177391-177431,numTag + 9 1.69e-05 GTGGAAAG ATGAGCGAG GACAGGTGTG

ebv:137151-137191,numTag + 4 1.69e-05 CCC GTGAGCAAT GGTGGGGACG

ebv:110091-110131,numTag + 17 1.69e-05 CATTTACCTG GTGAGCAAT CAGACATGCG

ebv:78731-78771,numTags= + 10 2.01e-05 GTTGACATC ATGAGCAAG GGCCCAGGGG

ebv:53711-53751,numTags= + 23 2.01e-05 GGGTGGAAAT ATGAGCAAG AATAAGGAC

ebv:175331-175371,numTag - 19 2.41e-05 ACAGGAGGAG CTGAGCGAT GAACCTGGCC

ebv:126791-126831,numTag - 5 2.41e-05 CCTCTGGGTG CTGAGCGAT GCCT

ebv:86531-86571,numTags= + 16 3.87e-05 CACGAGGGGC GTGAGCGAG CGCAGGGCCC

ebv:66611-66651,numTags= - 6 3.87e-05 GACGGCCATC GTGAGCGAG TACGT

ebv:58491-58531,numTags= + 26 5.44e-05 TCGTGACTTT GTGAGCCAT GACACA

ebv:56771-56811,numTags= + 5 5.44e-05 AGCT CTGAGCGAG ATTTTCCACA

ebv:166811-166851,numTag + 18 5.44e-05 AGGCATGTTG CTGAGCGAG CTCGTCGATC

ebv:119791-119831,numTag - 5 5.44e-05 CAGGAACACG CTGAGCGAG AAGA

ebv:162131-162171,numTag + 25 1.04e-04 ACGAGAGACT CTGTGCAAT CAAACCC

ebv:148191-148231,numTag - 6 1.04e-04 GGCGCTGACT CTGTGCAAT ATCGC

ebv:76111-76151,numTags= - 13 1.15e-04 ATGATGGATG GTGAGTAAT AAATTCTTAA

ebv:114491-114531,numTag + 3 1.15e-04 TC GTGTGCGAG CCGGTTTCGT

ebv:9151-9191,numTags=11 - 7 1.54e-04 TCAAGAGGGC ATTAGCAAT AGTGTT

ebv:176691-176731,numTag - 17 1.54e-04 ATGTGTTGAG ATGAGTCAT CCCGTGGAGA

ebv:150951-150991,numTag - 20 1.54e-04 CCCAGGGCAC ATTAGCAAT GTTCTAGGCA

ebv:150511-150551,numTag + 6 1.54e-04 ACTAA ATGAGTCAT TCCTAAACTG

ebv:49251-49291,numTags= + 10 1.68e-04 CTGTCTTGC ATGTGCCAG ACCAATCAAT

ebv:72411-72451,numTags= + 14 1.78e-04 AGGCGGGCGA ATGTGTAAT CCCGGAACCG

ebv:52831-52871,numTags= - 20 2.37e-04 GCCCCCACTC CTGAGCAAA CCTTAAAGTA

ebv:67951-67991,numTags= + 28 2.68e-04 TCGACCCTGC ATTTGCGAT GTAC

ebv:53611-53651,numTags= - 18 3.65e-04 CAGGGGCTTA GTGTGTCAT GGTGAGGCAG

ebv:174751-174791,numTag - 26 4.57e-04 ACTCTG GTTTGCGAG GCTGGGCGGC

ebv:122471-122511,numTag - 4 5.10e-04 GGCGGAGCAT ATGAGTCAA TTC

ebv:131351-131391,numTag - 21 5.48e-04 CACTGCCACT ATGAGACAT ATTATGTCTT

ebv:145211-145251,numTag + 17 6.25e-04 AGTTGTGTGA CTGTGCCAA TTTTCTTCGC

ebv:71511-71551,numTags= - 8 7.82e-04 ACCGCCGAAG ATGAGTAAC GGCGACT

ebv:64591-64631,numTags= + 10 9.46e-04 GAAGCACCG GCGTGCGAG GAGCAGCATG

ebv:110831-110871,numTag - 1 9.46e-04 GCCAATGTCT GCGTGCGAG

ebv:129591-129631,numTag + 27 1.27e-03 CTGAGCCGCA CTGTGCGTG ACGAT

--------------------------------------------------------------------------------

--------------------------------------------------------------------------------

Motif 1 block diagrams

--------------------------------------------------------------------------------

SEQUENCE NAME POSITION P-VALUE MOTIF DIAGRAM

------------- ---------------- -------------

ebv:106351-106391,numTag 3.2e-06 19_[+1]_12

ebv:127491-127531,numTag 5.7e-06 13_[+1]_18

ebv:83851-83891,numTags= 9.7e-06 15_[+1]_16

ebv:165011-165051,numTag 9.7e-06 3_[+1]_28

ebv:84631-84671,numTags= 1.7e-05 13_[+1]_18

ebv:177391-177431,numTag 1.7e-05 8_[+1]_23

ebv:137151-137191,numTag 1.7e-05 3_[+1]_28

ebv:110091-110131,numTag 1.7e-05 16_[+1]_15

ebv:78731-78771,numTags= 2e-05 9_[+1]_22

ebv:53711-53751,numTags= 2e-05 22_[+1]_9

ebv:175331-175371,numTag 2.4e-05 18_[-1]_13

ebv:126791-126831,numTag 2.4e-05 4_[-1]_27

ebv:86531-86571,numTags= 3.9e-05 15_[+1]_16

ebv:66611-66651,numTags= 3.9e-05 5_[-1]_26

ebv:58491-58531,numTags= 5.4e-05 25_[+1]_6

ebv:56771-56811,numTags= 5.4e-05 4_[+1]_27

ebv:166811-166851,numTag 5.4e-05 17_[+1]_14

ebv:119791-119831,numTag 5.4e-05 4_[-1]_27

ebv:162131-162171,numTag 0.0001 24_[+1]_7

ebv:148191-148231,numTag 0.0001 5_[-1]_26

ebv:76111-76151,numTags= 0.00011 12_[-1]_19

ebv:114491-114531,numTag 0.00011 2_[+1]_29

ebv:9151-9191,numTags=11 0.00015 6_[-1]_25

ebv:176691-176731,numTag 0.00015 16_[-1]_15

ebv:150951-150991,numTag 0.00015 19_[-1]_12

ebv:150511-150551,numTag 0.00015 5_[+1]_26

ebv:49251-49291,numTags= 0.00017 9_[+1]_22

ebv:72411-72451,numTags= 0.00018 13_[+1]_18

ebv:52831-52871,numTags= 0.00024 19_[-1]_12

ebv:67951-67991,numTags= 0.00027 27_[+1]_4

ebv:53611-53651,numTags= 0.00037 17_[-1]_14

ebv:174751-174791,numTag 0.00046 25_[-1]_6

ebv:122471-122511,numTag 0.00051 3_[-1]_28

ebv:131351-131391,numTag 0.00055 20_[-1]_11

ebv:145211-145251,numTag 0.00063 16_[+1]_15

ebv:71511-71551,numTags= 0.00078 7_[-1]_24

ebv:64591-64631,numTags= 0.00095 9_[+1]_22

ebv:110831-110871,numTag 0.00095 [-1]_31

ebv:129591-129631,numTag 0.0013 26_[+1]_5

--------------------------------------------------------------------------------

--------------------------------------------------------------------------------

Motif 1 in BLOCKS format

--------------------------------------------------------------------------------

BL MOTIF 1 width=9 seqs=39

ebv:106351-106391,numTag ( 20) ATGAGCGAT 1

ebv:127491-127531,numTag ( 14) ATGAGCAAT 1

ebv:83851-83891,numTags= ( 16) GTGAGCGAT 1

ebv:165011-165051,numTag ( 4) GTGAGCGAT 1

ebv:84631-84671,numTags= ( 14) ATGAGCGAG 1

ebv:177391-177431,numTag ( 9) ATGAGCGAG 1

ebv:137151-137191,numTag ( 4) GTGAGCAAT 1

ebv:110091-110131,numTag ( 17) GTGAGCAAT 1

ebv:78731-78771,numTags= ( 10) ATGAGCAAG 1

ebv:53711-53751,numTags= ( 23) ATGAGCAAG 1

ebv:175331-175371,numTag ( 19) CTGAGCGAT 1

ebv:126791-126831,numTag ( 5) CTGAGCGAT 1

ebv:86531-86571,numTags= ( 16) GTGAGCGAG 1

ebv:66611-66651,numTags= ( 6) GTGAGCGAG 1

ebv:58491-58531,numTags= ( 26) GTGAGCCAT 1

ebv:56771-56811,numTags= ( 5) CTGAGCGAG 1

ebv:166811-166851,numTag ( 18) CTGAGCGAG 1

ebv:119791-119831,numTag ( 5) CTGAGCGAG 1

ebv:162131-162171,numTag ( 25) CTGTGCAAT 1

ebv:148191-148231,numTag ( 6) CTGTGCAAT 1

ebv:76111-76151,numTags= ( 13) GTGAGTAAT 1

ebv:114491-114531,numTag ( 3) GTGTGCGAG 1

ebv:9151-9191,numTags=11 ( 7) ATTAGCAAT 1

ebv:176691-176731,numTag ( 17) ATGAGTCAT 1

ebv:150951-150991,numTag ( 20) ATTAGCAAT 1

ebv:150511-150551,numTag ( 6) ATGAGTCAT 1

ebv:49251-49291,numTags= ( 10) ATGTGCCAG 1

ebv:72411-72451,numTags= ( 14) ATGTGTAAT 1

ebv:52831-52871,numTags= ( 20) CTGAGCAAA 1

ebv:67951-67991,numTags= ( 28) ATTTGCGAT 1

ebv:53611-53651,numTags= ( 18) GTGTGTCAT 1

ebv:174751-174791,numTag ( 26) GTTTGCGAG 1

ebv:122471-122511,numTag ( 4) ATGAGTCAA 1

ebv:131351-131391,numTag ( 21) ATGAGACAT 1

ebv:145211-145251,numTag ( 17) CTGTGCCAA 1

ebv:71511-71551,numTags= ( 8) ATGAGTAAC 1

ebv:64591-64631,numTags= ( 10) GCGTGCGAG 1

ebv:110831-110871,numTag ( 1) GCGTGCGAG 1

ebv:129591-129631,numTag ( 27) CTGTGCGTG 1

//

--------------------------------------------------------------------------------

--------------------------------------------------------------------------------

Motif 1 position-specific scoring matrix

--------------------------------------------------------------------------------

log-odds matrix: alength= 4 w= 9 n= 1344 bayes= 6.00546 E= 2.9e-034

90 -13 25 -1193

-1193 -245 -1193 211

-1193 -1193 168 -110

165 -1193 -1193 48

-1193 -1193 184 -1193

-310 151 -1193 -30

60 -45 72 -1193

214 -1193 -1193 -310

-152 -344 46 122

--------------------------------------------------------------------------------

--------------------------------------------------------------------------------

Motif 1 position-specific probability matrix

--------------------------------------------------------------------------------

letter-probability matrix: alength= 4 w= 9 nsites= 39 E= 2.9e-034

0.410256 0.256410 0.333333 0.000000

0.000000 0.051282 0.000000 0.948718

0.000000 0.000000 0.897436 0.102564

0.692308 0.000000 0.000000 0.307692

0.000000 0.000000 1.000000 0.000000

0.025641 0.794872 0.000000 0.179487

0.333333 0.205128 0.461538 0.000000

0.974359 0.000000 0.000000 0.025641

0.076923 0.025641 0.384615 0.512821

--------------------------------------------------------------------------------

--------------------------------------------------------------------------------

Motif 1 regular expression

--------------------------------------------------------------------------------

[AGC]TG[AT]GC[GAC]A[TG]

--------------------------------------------------------------------------------
